# Evolution combined with genomic study elucidates genetic bases of isobutanol tolerance in *Escherichia coli*

**DOI:** 10.1186/1475-2859-10-18

**Published:** 2011-03-25

**Authors:** Jeremy J Minty, Ann A Lesnefsky, Fengming Lin, Yu Chen, Ted A Zaroff, Artur B Veloso, Bin Xie, Catie A McConnell, Rebecca J Ward, Donald R Schwartz, Jean-Marie Rouillard, Yuan Gao, Erdogan Gulari, Xiaoxia Nina Lin

**Affiliations:** 1 Department of Chemical Engineering, University of Michigan, Ann Arbor, MI 48109, USA; 2 School of Chemical Engineering and Technology, Tianjin University, Tianjin 300072, PR China; 3 Bioinformatics Graduate Program, University of Michigan Medical School, Ann Arbor, MI 48109, USA; 4 Center for the Study of Biological Complexity, Virginia Commonwealth University, VA 23284, USA; 5 MYcroarray, 5692 Plymouth Road, Ann Arbor, MI 48105, USA; 6 Department of Biomedical Engineering, University of Michigan, Ann Arbor, MI 48109, USA; 7 Center for Computational Medicine and Bioinformatics, University of Michigan Medical School, Ann Arbor, MI 48109, USA

## Abstract

**Background:**

Isobutanol is a promising next-generation biofuel with demonstrated high yield microbial production, but the toxicity of this molecule reduces fermentation volumetric productivity and final titer. Organic solvent tolerance is a complex, multigenic phenotype that has been recalcitrant to rational engineering approaches. We apply experimental evolution followed by genome resequencing and a gene expression study to elucidate genetic bases of adaptation to exogenous isobutanol stress.

**Results:**

The adaptations acquired in our evolved lineages exhibit antagonistic pleiotropy between minimal and rich medium, and appear to be specific to the effects of longer chain alcohols. By examining genotypic adaptation in multiple independent lineages, we find evidence of parallel evolution in *marC*, *hfq*, *mdh*, *acrAB, gatYZABCD*, and *rph *genes. Many isobutanol tolerant lineages show reduced RpoS activity, perhaps related to mutations in *hfq *or *acrAB*. Consistent with the complex, multigenic nature of solvent tolerance, we observe adaptations in a diversity of cellular processes. Many adaptations appear to involve epistasis between different mutations, implying a rugged fitness landscape for isobutanol tolerance. We observe a trend of evolution targeting post-transcriptional regulation and high centrality nodes of biochemical networks. Collectively, the genotypic adaptations we observe suggest mechanisms of adaptation to isobutanol stress based on remodeling the cell envelope and surprisingly, stress response attenuation.

**Conclusions:**

We have discovered a set of genotypic adaptations that confer increased tolerance to exogenous isobutanol stress. Our results are immediately useful to further efforts to engineer more isobutanol tolerant host strains of *E. coli *for isobutanol production. We suggest that *rpoS *and post-transcriptional regulators, such as *hfq*, RNA helicases, and sRNAs may be interesting mutagenesis targets for future global phenotype engineering.

## Background

With shrinking fossil fuel supplies, accelerating climate change, and intensifying geopolitical concerns, the need for renewable energy sources and commodity chemicals is becoming evermore apparent. Microbial production of biofuels and commodity chemicals from lignocellulosic biomass or direct photosynthetic conversion from CO_2 _could sustainably replace traditional production platforms based on fossil fuel feedstocks, but tremendous research efforts are still needed in engineering robust and productive organisms [1]. Advances in metabolic engineering have dramatically expanded the portfolio of fuel and commodity chemicals that can be produced biologically. Efforts towards biological production of higher molecular weight alcohols as next-generation biofuels have been particularly successful; *Escherichia coli *has been successfully engineered to produce isobutanol in high yield (86% theoretical) from carbohydrates, and direct photosynthetic conversion of CO_2 _to isobutanol has been demonstrated with engineered strains of the cyanobacterium *Synechococcus elongates *[2,3]. Isobutanol is a promising biofuel molecule and has many advantages over ethanol, including high energy density, low hygroscopicity, desirable combustion properties, and demonstrated high yield production [4]. However, isobutanol is toxic to microbes; concentrations of 1.25% (w/v) completely inhibit growth of *E. coli *in minimal media at 30°C (unpublished data). Isobutanol toxicity limits final product titer and volumetric productivity in fermentation, thus motivating efforts to engineer bacterial hosts with improved tolerance [5].

Numerous investigations have elucidated mechanisms of toxicity and proximal cellular responses to alcohol stress. Alcohols intercalate into the membrane lipid bilayer, perturbing the physicochemical properties of membrane [6]. Membrane fluidity and permeability are altered, and membrane proteins may be displaced or denatured; these changes can lead to dissipation of membrane electrochemical potential and proton gradient, and disruption of membrane based processes such as substrate transport and energy generation [6]. Other chaotropic effects of alcohols also contribute to toxicity, for instance through denaturation of cytosolic proteins [6]. Various cellular responses to alcohol stress have been observed, including induction of general stress response (such as upregulation of chaperonins), active efflux of alcohols, synthesis of protective metabolites, alteration of membrane and cell surface properties, adaptations in energy metabolism, changes in cellular morphology, and metabolic degradation of alcohols; some or all of these responses may be present in a given organism [6]. Systems biology studies of *E. coli *response to isobutanol and the closely related compound n-butanol have revealed that multiple stress response systems are induced by these alcohols, leading to global changes in gene transcription and proteome composition. Network Component Analysis (NCA) was used to map the initial transcriptional response of *E. coli *to isobutanol, identifying ArcA as the most affected transcription factor; follow-up studies indicated that ArcA activation may proceed via isobutanol-induced quinone disruption [7]. The transcriptomic and proteomic response of *E. coli *to n-butanol stress has been characterized, with especially strong induction of oxidative and cell envelope stress responses observed; it was subsequently demonstrated that n-butanol exposure results in increased intracellular generation of reactive oxygen species, and oxidative stress gene knockouts led to decreased tolerance [8].

Due to the broad mechanisms of toxicity, tolerance to alcohols and other solvents is a complex trait that involves a diversity of cellular adaptations and responses that probably contribute synergistically to the overall phenotype [6]. While the cellular response to alcohols has been characterized, translating this understanding into rational methods for engineering alcohol tolerance has not come to full fruition; the inherent biological complexity hampers efforts to rationally engineer improved strains [6]. Most strategies for investigating and improving solvent tolerance are therefore combinatorial in nature, following a paradigm of generating phenotypic diversity in a population, then characterizing isolates with the desired properties [9]. For example, transposon mutagenesis libraries have been screened for alcohol tolerant isolates [6,10,11] and plasmid-based genomic library enrichment studies have been used to investigate genetic bases of alcohol tolerance [6,10,12]. Furthermore, targeted mutagenesis of master transcriptional regulators in *Escherichia coli *has been used to generate libraries of mutants with global perturbations in gene expression, from which highly ethanol and n-butanol tolerant clones have been isolated [6,13,14]. The above powerful approaches have generated significant insights about alcohol tolerance and/or created strains with elevated tolerance. However, these methods explore only relatively small subsets of the possible genotype space; new approaches are needed to further elucidate and improve alcohol tolerance by taking into account the multigenic nature of this phenotype and expanding the accessible genotype space.

Recent advances in DNA sequencing technology have led to dramatically increased throughput, enabling rapid and relatively inexpensive resequencing of microbial genomes [15]. An intriguing corollary to these technological advances is the prospect of using whole genome resequencing to characterize the genetic bases of adaptation in evolved strains. This approach was recently employed to investigate adaptation of *E. coli *in several experimental evolutions studies, including short-term evolution on glycerol, lactate, and L-1,2-propanediol carbon sources, adaptation to high radiation levels, and long-term genome evolution over 40,000 generations [16-20]. In this report, we comprehensively investigate adaptations to exogenous isobutanol stress in *E. coli *by experimental evolution followed by genome resequencing and a gene expression study. Using this approach, we have identified key loci involved in isobutanol tolerance. Consistent with the complex, multigenic nature of isobutanol tolerance, we find diverse and often surprising genetic adaptations to isobutanol stress that were not obvious from other approaches to investigating tolerance. The divergent growth phenotypes of the end populations and studies with single and multiple mutation reconstructions suggest a rugged fitness landscape with many epistatic interactions. When conducting our study, we became aware of a similar project concurrently underway in another laboratory [21]. Their study revealed another distinct set of genetic loci in *E. coli *related to isobutanol tolerance in yeast extract supplemented media, which shows both partial overlap with and significant difference from our discoveries under minimal media conditions. By elucidating candidate loci and cellular processes that are under selective pressure for isobutanol tolerance, these results are immediately useful to efforts towards engineering robust strains of *E. coli *for isobutanol production.

## Results

### Experimental evolution and phenotypic characterization of end populations

*E. coli *EcNR1, a derivative of *E. coli *K12 MG1655 harboring a λ Red prophage integrated at the *bio *locus, was evolved by serial passaging of six independent populations for approximately 500 generations on isobutanol spiked M9 minimal medium supplemented with 50 g/L carbon source and 0.25 mg/L biotin. An initial isobutanol concentration of 0.75% (w/v) (corresponding to approximately 75% growth inhibition) was used for all populations, providing strong selective pressure. Isobutanol concentration was gradually increased during evolution to maintain approximately constant selective pressure. Populations were evolved on two different carbon sources, with three populations evolved with 50 g/L glucose as the sole carbon source (designated glucose #1, glucose #2, and glucose #3, abbreviated G1, G2, G3) and another three populations evolved with 50 g/L xylose as the sole carbon source (designated xylose #1, xylose #2, and xylose #3, abbreviated X1, X2, X3). Glucose and xylose are important constituents of lignocellulosic feedstocks and are metabolized by different pathways; thus we explore adaptations in different metabolic contexts relevant to biofuel production [1]. Cultures from each evolving population were periodically archived by cryopreservation, and phenotyped by measuring maximum specific growth rate (μ_max_, h^-1^) at various isobutanol concentrations using a microplate spectrophotometer.

**Figure 1:**
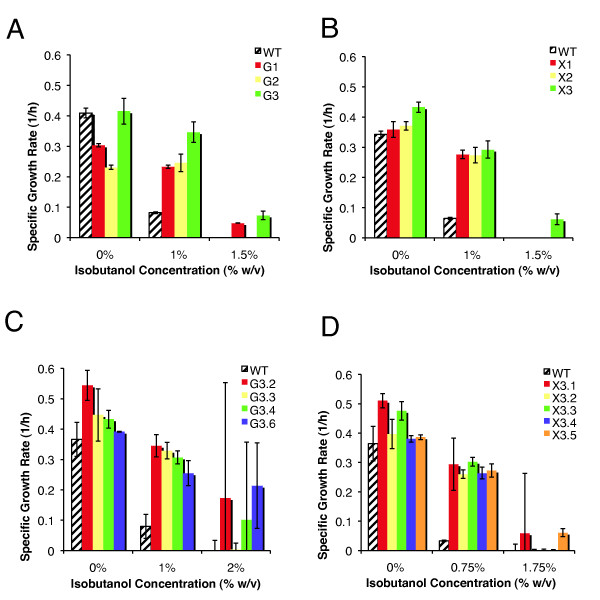
**Isobutanol tolerance phenotype of evolution end populations**. Isobutanol tolerance of evolution end populations was evaluated by measuring growth rate at various isobutanol concentrations, with the parent *E. coli *EcNR1 included as a reference. Populations were phenotyped with the same carbon source they were evolved in. **(A) **populations evolved on glucose as sole carbon sources (three lineages, G1, G2, G3), **(B) **populations evolved on xylose as sole carbon sources (three lineages, X1, X2, X3), **(C) **selected clonal isolates obtained from G3 population, **(D) **selected clonal isolates obtained from X3 population.

All of the evolved populations show significantly improved fitness at high isobutanol concentrations relative to the parent *E. coli *EcNR1 strain (WT) (Figure 1A and 1B). Interestingly, the populations show divergent growth phenotypes. Clonal isolates from two highly tolerant populations, G3 (glucose #3 population) and X3 (xylose #3 population), were further phenotyped, revealing significant heterogeneity within these populations (Figure 1C and 1D). Three clones from G3 were capable of growth at 2% isobutanol in glucose media and two clones from X3 grew at 1.75% isobutanol in xylose media, representing 60% and 40% improvements in tolerance respectively, compared to WT (Figure 1C and 1D). Two representative clones with high fitness, G3.2 and X3.5, were chosen for further characterization.

**Figure 2:**
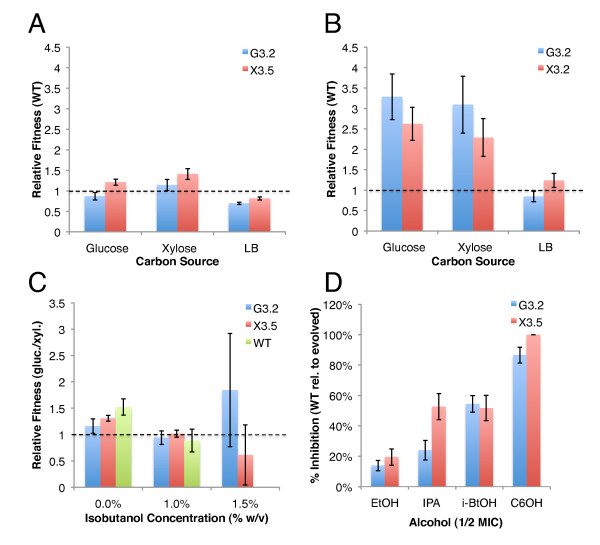
**Specificity of adaptation with different carbon sources and alcohols**. Specificity of adaptation of clones G3.2 (isolated from G3 evolution end population) and X3.5 (isolated from X3 evolution end population) was examined by assessing isobutanol tolerance of each clone and the parent *E. coli *EcNR1 on glucose minimal medium, xylose minimal medium, and LB medium. Tolerance to ethanol, isopropanol, and n-hexanol was also determined for these strains and the parent *E. coli *EcNR1. **(A) **Fitness (relative to parent *E. coli *EcNR1) of G3.2 and X3.5 on glucose, xylose, and LB medium with 0% (w/v) isobutanol, **(B) **Fitness (relative to parent *E. coli *EcNR1) of G3.2 and X3.5 on glucose, xylose, and LB medium with 1% (w/v) isobutanol, **(C) **Fitness of G3.2 and X3.5 (in glucose vs. xylose) at 0%, 1%, and 1.5% (w/v) isobutanol, **(D) **Percent relative inhibition of *E. coli *EcNR1 compared to G3.2 and X3.5 (defined as , where μ_WT _is the maximum specific growth rate of *E. coli *EcNR1, and μ_MUT _is the maximum specific growth rate of G3.2 or X3.5) on ethanol (3.5% v/v), isopropanol (2.5% v/v), isobutanol (0.5% w/v), and hexanol (0.25% v/v). The alcohol concentrations correspond to approximately 1/2 the minimum growth inhibiting concentration (MIC) for the parent *E. coli *EcNR1 strain.

Evolution often produces adaptations that show tradeoffs in relative fitness across different environments [22]. To investigate specificity of adaptation, the fitness (relative to WT) of clones G3.2 and X3.5 at 0% and 1% (w/v) isobutanol was assessed on minimal glucose, minimal xylose, and rich LB media (Figure 2A and 2B). At 0% (w/v) isobutanol both G3.2 and X3.5 show improved fitness on xylose minimal medium and decreased fitness on LB medium, relative to WT (Figure 2A). At 1% (w/v) isobutanol, G3.2 and X3.5 show markedly improved relative fitness on both glucose and xylose minimal media, and decreased fitness in LB medium (Figure 2B). These results suggest that the two isolates characterized have accumulated adaptations to isobutanol stress specific to minimal media, and these adaptations appear to exhibit antagonistic pleiotropy in rich medium. This minimal-rich medium antagonistic pleiotropy we observed underscores the importance of carefully selecting evolution conditions. On the other hand, although G3.2 and X3.5 were evolved on glucose and xylose media respectively, neither of these strains appears to have developed carbon-source specific adaptations in 0% and 1% isobutanol environments. We further assayed fitness in glucose and xylose minimal media at higher isobutanol concentrations (Figure 2C). At 1.5% (w/v) isobutanol, we observed relative fitness trends suggesting greater specificity of adaptation for G3.2 and X3.5 to their respective carbon sources, but we could not substantiate that these differences were statistically significant due to the error bars in our measurements (Figure 2C). Interestingly, at 0% isobutanol X3.5 appears to have higher relative fitness than G3.2 in all media types tested (Figure 2A). ATP yield from xylose metabolism is lower compared to glucose metabolism, and we speculate that low ATP yield increases selective pressure for more energy efficient use of carbon sources [23]. This may explain how adaptations to a low ATP yield substrate such as xylose could also be beneficial to growth on other carbon sources.

In addition to investigating specificity of adaptation to different carbon sources, we also examined the tolerance of G3.2 and X3.5 to various alcohols with potential for microbial biofuel production, including ethanol, isopropanol, and n-hexanol (Figure 2D). While all alcohols share the same general mechanisms of toxicity via chaotropic effects and interactions with membrane lipid bilayers, specific biophysical effects are known to vary with alcohol chain length [24]. Molecular dynamics simulations and experiments with model lipid bilayers have demonstrated that long chain alcohols (≥ C8) tend to condense and stiffen lipid bilayers, while short chain alcohols (≤ C2) have opposite effects [24]; lipid bilayer interactions with intermediate length and branched alcohols (such as isobutanol) have not been well characterized. We examined the percent relative inhibition of WT compared to G3.2 and X3.5 (defined as , where μ_WT _is the maximum specific growth rate of *E. coli *EcNR1, and μ_MUT _is the maximum specific growth rate of G3.2 or X3.5) at 3.5% (v/v) ethanol, 2.5% (v/v) isopropanol, 0.5% (w/v) isobutanol, and 0.25% (v/v) n-hexanol; concentrations were chosen to correspond to approximately 1/2 of the minimum growth inhibiting concentration (MIC) on glucose minimal medium at 30°C. For all alcohols assayed, G3.2 and X3.5 displayed higher tolerance than WT; interestingly, the relative inhibition of WT increased with increasing chain length (hexanol > isobutanol ≥ isopropanol ≥ ethanol), indicating the adaptations to isobutanol stress may be selective to the effects of longer chain alcohols.

### Genome resequencing of isobutanol tolerant clones

To identify the genetic bases of adaptation to isobutanol stress, we resequenced the genomes of highly tolerant clones from our evolved populations with the Illumina Solexa platform, using 36 base pair single-end or paired-end read configurations. 612 to 756 million base pairs (MB) of raw sequence was generated for each sequenced genome with single-end read configuration, with approximately four times as much sequence generated with paired-end reads. Coverage averaged approximately 125× and 500× for the 4.65 MB *E. coli *EcNR1 genome, using single-end and paired-end reads, respectively. Reads were mapped to the *E. coli *EcNR1 reference genome sequence using Novoalign v2.04.02 and MAQ v0.7; single nucleotide polymorphisms (SNPs) and short insertion/deletions (indels) were called from the consensus sequence [25,26]. Larger indels were detected by examining coverage distribution. Unmapped reads were collected and *de novo *assembled using Velvet v0.7.51 to detect breakpoints near sites of structural variation (SV) [27].

**Figure 3:**
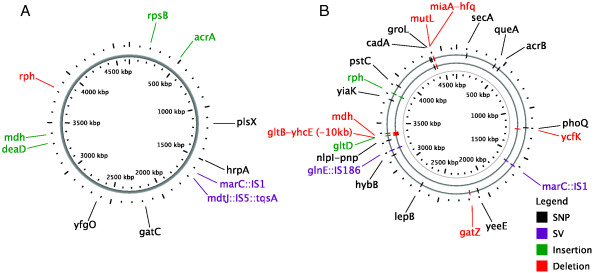
**Chromosome maps of mutations discovered through whole genome resequencing of isobutanol tolerant clones**. Genome resequencing of clones G3.2 and G3.6 (from G3 end population), X3.5 (from X3 end population), and G3.266.7 (from G3 lineage, generation 266) was done using the Illumina Solexa platform. SNPs (single nucleotide polymorphisms) and short indels (nucleotide insertions or deletions) were detected by mapping reads were onto the reference *E. coli *EcNR1 genome sequence. Structural Variation (SV) breakpoints were detected by *de novo *assembly of unmapped reads for single-end sequence data or with BreakDancer v0.0.1 for paired-end sequence data. **(A) **Chromosome map of mutations detected in X3.5; **(B) **Chromosome map of overlapping mutations in G3.2/G3.6 and G3.266.7. Mutations in G3.2/G3.6 are shown on the outer ring, while mutations in G3.266.7 are shown on the inner ring. All mutations in G3.266.7 except for the *gltB*-*yhcE *10 kb deletion and *ycfK *1688 bp deletion are on the same line of descent as G3.2 and G3.6 clones.

We resequenced the genomes of G3.2, G3.6, and X3.5, three highly isobutanol tolerant clones from the evolution end populations (discovered mutations summarized in Figure 3 and Table 1; full mutation lists available in Additional file 1 and the reference genome sequence in Additional file 2). It was discovered that the G3 lineage had acquired a 19 bp deletion in *mutL*, a component of the methyl-directed mismatch repair system (MMR). MMR loss-of-function mutations lead to an approximately 100-fold increase in mutation rate, giving rise to the so-called mutator phenotypes [22]. Subsequently G3.2 and G3.6 were highly mutated, having 48 and 64 mutations respectively, with 20 mutations in common between these two clones (Figure 3B and Table 1). To narrow down candidate mechanisms of genetic adaptation, we resequenced the genome of a non-mutator clonal isolate from generation 266 of the G3 lineage (G3.266.7) and identified 8 mutations in this clone (Figure 3B and Table 1). For X3.5, 11 mutations were revealed (Figure 3A and Table 1).

**Table 1:** Mutations discovered in genome resequencing of evolved isobutanol tolerant *E. coli *clones

Clone	Gene	Gene Description	Biological Process	Cellular Location	Genomic Coordinate	Nucleotide Change	Protein Change
**G3.2/G3.6**	*phoQ*	sensory histidine kinase in two-component regulatory system with PhoP	Signal transduction	Inner membrane	1197581	A→G	L209P
	***acrB***	***multidrug efflux system protein***	***Transport***	***Inner and outer membrane***	***480665***	***G→A***	***P988L***
	*queA*	S-adenosylmethionine:tRNA ribosyltransferase-isomerase (EC:5.-.-.-)	Translation	Cytoplasm	425270	A→G	N346D
	*secA*	preprotein translocase subunit, ATPase	Protein secretion	Inner membrane	108975	T→C	S233P
	*cadA*	lysine decarboxylase 1 (EC:4.1.1.18)	Amino acid metabolism	Cytoplasm	4363790	A→G	I686T
	*groL*	Cpn60 chaperonin GroEL, large subunit of GroESL	Protein folding	Cytoplasm	4378650	A→C	K132N
	*mutL*	methyl-directed mismatch repair protein	Mismatch repair	Cytoplasm	4405650	-19 bp	Frameshift
	*pstC*	phosphate transporter subunit	Transport	Inner membrane	3917582	T→C	D16G
	***rph***	***defective ribonuclease PH***	***RNA processing***	***Cytoplasm***	***3823229***	***+4:GGTC***	***Frameshift***
	*yiaK*	2,3-diketo-L-gulonate dehydrogenase, NADH-dependent (EC:1.1.1.-)	Carbohydrate metabolism	Cytoplasm	3750540	T→C	L193P
	*gltD*	glutamate synthase, 4Fe-4S protein, small subunit (EC:1.4.1.13)	Amino acid, Nitrogen metabolism	Cytoplasm	3367270	+1:G	Frameshift
	***mdh***	***malate dehydrogenase, NAD(P)-binding (EC:1.1.1.37)***	***Tricarboxylic acid cycle***	***Membrane peripheral***	***3390726***	***-1:C***	***Frameshift***
	*nlpI*	lipoprotein involved in cell division	Cell cycle	Inner membrane	3316213	T→C	Non-coding region; Possible effect on terminator before nlpI
	*glnE*	fused deadenylyltransferase/adenylyltransferase for glutamine	Nitrogen metabolism	Cytoplasm	3205272	IS186 insertion	Disruption
	*hybB*	predicted hydrogenase 2 cytochrome b type component	Electron transport chain	Inner membrane	3150318	A→G	V359A
	***gatZ***	***D-tagatose 1,6-bisphosphate aldolase 2, subunit (EC:4.1.2.40)***	***Carbohydrate metabolism***	***Cytoplasm***	***2182915***	***-1:C***	***Frameshift***
	*yeeE*	predicted inner membrane protein	-	Inner membrane	2092513	A→G	S333P
	*lepB*	leader peptidase (signal peptidase I) (EC:3.4.21.89)	Transport	Inner membrane	2711902	G→A	P213S
	*hfq*	HF-I, host factor for RNA phage Q β replication	Translation	Cytoplasm	4407505	-7:AGGAAAA	Non-coding region; Ribosome binding site deletion
	***marC***	***conserved protein; predicted transporter***	-	***Inner membrane***	***1625925***	***IS1 insertion***	***Disruption***
**G3.266.7**	*groL*	Cpn60 chaperonin GroEL, large subunit of GroESL	Protein folding	Cytoplasm	4378650	A→C	K132N
	***rph***	***defective ribonuclease PH***	***RNA processing***	***Cytoplasm***	***3823229***	***+4:GGTC***	***Frameshift***
	*gltB-yhcE*	-	-	-	-	-9.9 kb	ΔgltBDF, ΔyhcADE
	***mdh***	***malate dehydrogenase, NAD(P)-binding (EC:1.1.1.37)***	***Tricarboxylic acid cycle***	***Membrane peripheral***	***3390726***	***-1:C***	***Frameshift***
	*glnE*	fused deadenylyltransferase/adenylyltransferase for glutamine	Nitrogen metabolism	Cytoplasm	3205272	IS186 insertion	Disruption
	*hfq*	HF-I, host factor for RNA phage Q β replication	Translation	Cytoplasm	4407505	-7:AGGAAAA	Non-coding region; Ribosome binding site deletion
	*ycfK*	e14 prophage; predicted protein	-	-	1216432	-1688 bp	ΔycfK
	***marC***	***conserved protein; predicted transporter***	-	***Inner membrane***	***1625925***	***IS1 insertion***	***Disruption***
**X3.5**	***acrA***	***multidrug efflux system protein***	***Transport***	***Inner and outer membrane***	***483735***	***+1:A***	***Frameshift***
	*rpsB*	30S ribosomal subunit protein S2	Translation	Cytoplasm	190557	+1:A	Frameshift
	***rph***	***defective ribonuclease PH***	***RNA processing***	***Cytoplasm***		***-1:C***	***Frameshift***
	***mdh***	***malate dehydrogenase, NAD(P)-binding (EC:1.1.1.37)***	***Tricarboxylic acid cycle***	***Membrane peripheral***	***3390936***	***+5:AACCT***	***Frameshift***
	*deaD*	DEAD-box RNA helicase	Translation	Cytoplasm	3314027	+4:AGAC	Frameshift
	*yfgO*	predicted inner membrane protein	-	Inner membrane	2623022	C→T	G30D
	***gatC***	***galactitol-specific enzyme IIC component of PTS***	***Transport***	***Inner membrane***	***2180640***	***C→T***	***E290K***
	*plsX*	fatty acid/phospholipid synthesis protein	Fatty acid metabolism	Cytoplasm	1493514	A→G	E216G
	*hrpA*	ATP-dependent helicase	RNA processing	Cytoplasm	1493514	C→T	L1075L
	*mdtJ-tqsA*	MdtJ SMR protein; transporter of quorum signal AI-2	Transporter/Transporter	Inner membrane	1681114	IS5 insertion	Non-coding region; mdtJ and tqsA promoter region
	***marC***	***conserved protein; predicted transporter***	-	***Inner membrane***	***1626081***	***IS1 insertion***	***Disruption***

Clonal isolates from isobutanol tolerant *E. coli *EcNR1 populations were sequenced with the Illumina platform to identify mutations. Clones G3.2 and G3.6 were taken from the G3 end point population, which developed a mutator phenotype via a 19 bp deletion in *mutL*. These clones thus each have a large number of mutations, so for brevity we show here the subset of mutations shared between G3.2/G3.6 (full mutation lists available in Additional file 1). All mutations shown above were verified by Sanger sequencing. Mutation entries that are bold and italic denote loci that were mutated in all sequenced clones from end populations. The mutation positions are listed as absolute genomic coordinates in the *E. coli *EcNR1 reference sequence (available in Additional file 2). SNPs are indicated by base transition/transversion. Small insertions are indicated by a '+', with the size (number of bp) of the insertion and sequence of inserted bases. Small deletions are designated by '-' with a format similar to that for small insertions; for large deletions, the sequence of deleted bases is excluded. Transposons are indicated by the insertion sequence (IS) identity.

A total of 131 mutations were discovered across clones X3.5, G3.2, G3.6, and G3.266.7 (full list available in Additional file 1). 96 mutations were SNPs, 25 mutations were short indels, and 10 mutations were SVs. Most mutations occurred in the coding region of genes. The detected SVs consisted of transposon insertions (*marC::IS1 *in all sequenced isolates, *glnE::IS186 *in the G3 clones, and *mdtJ::IS5::tqsA *in X3.5), an approximately 10 kb deletion between *gltB *and *yhcE *in G3.266.7, and a 1688 bp deletion in the *ycfK *gene of the e14 prophage in G3.266.7. Mutations were found in diverse genetic loci representing many cellular processes. BiNGO (Biological Network Gene Ontology tool) was used to assess any overrepresented Gene Ontology (GO) terms in the full mutation set, but the only statistically significant finding was an enrichment of membrane proteins (corrected p-value = 7.23 × 10^-3^), with a borderline significant enrichment of RNA helicases (corrected p-value = 7.22 × 10^-2^) [28].

### Parallel evolution

Comparison of the genotypes of X3.5, G3.2, G3.6, and G3.266.7 reveals a number of parallel genotypic adaptations. In particular, mutations in *rph, acrAB, marC, mdh*, and the *gatYZABCD *operon were found in all of these clones (Table 1). *E. coli *K12 MG1655 (the parent strain of *E. coli *EcNR1) has a 1 bp deletion in the *rph-pyrE *operon, resulting in reduced levels of orotate phosphoribosyltransferase (the product of *pyrE*) and subsequently suboptimal pyrimidine biosynthesis levels [17]. Thus restorative mutations are commonly observed in *rph-pyrE *during experimental evolution studies with *E. coli *K12 MG1655, and are general adaptations to growth on minimal media. The AcrAB proteins are components of the AcrAB-TolC multidrug efflux pump, a membrane transporter which translocates a wide range of substrates out of the cytoplasmic membrane and periplasmic space; efflux via the AcrAB-TolC complex has been previously identified as an important mechanism of tolerance to organic solvents such as toluene, immediately suggesting a possible role for *acrAB-tolC *in isobutanol tolerance [6]. Possible links to isobutanol tolerance are not as obvious for *marC *(a predicted membrane protein of unknown function)*, mdh *(NADH dependent malate dehydrogenase), and the *gatYZABCD *operon, which encodes proteins involved in galactitol transport and catabolism.

To investigate possible parallel genotypic adaptations in our other evolved lineages, the *acrAB *operon, *tolC*, and *mdh *were sequenced in 8 clonal isolates from each of the evolved endpoint populations (Table 2). The *marC *locus was also sequenced in each endpoint population; examination of PCR products revealed indel mutations (discernable by product size) at near 100% allele frequency, allowing for whole population samples to be sequenced (Table 2). We also sequenced the post-transcriptional regulator *hfq *in our endpoint populations since an *hfq *mutation was found in G3, and modulation of *hfq *has been observed as a common mechanism of adaptation in other experimental evolution studies. *rph *and *gatYZABCD *were not investigated further since *rph-pyrE *adaptations have been characterized in previous works, while the relatively large size of the *gatYZABCD *operon was prohibitive for Sanger sequencing.

**Table 2:** Investigation of parallel genotypic adaptation in evolution endpoint populations

	Gene					
	
Population	*acrA*	*acrB*	*tolC*	*mdh*	*hfq*	*marC*
**X1**	-	481310 A→C (V773G)	-	-	4407590 T→G (I24M)	1625925 IS1 insertion (Disruption)

**X2**	484383 T→G (N154T)	-	-	3390659 +4:GATT (Frameshfit)	-	1626084 IS5 insertion (Disruption)

**X3**	483735 +1:A (Frameshift)	-	-	3390936 +5:AACCT (Frameshift)	-	1626081 IS1 insertion (Disruption)

**G1**	484669 G→T (R59S)	-	-	-	-	1625925 IS1 insertion (Disruption)

**G2**	-	-	-	-	-	1626100 -6:CCACCA (Deletion of V13 and V14)

**G3**	-	480665 G→A (P988L)	-	3390726 -1:C (Frameshift)	4407505 -7:AGGAAAA (RBS deletion)	1625925 IS1 insertion (Disruption)

Parallel genotypic adaptation in isobutanol tolerant *E. coli *EcNR1 endpoint populations was investigated by direct sequencing of loci of interest in sets of clonal isolates from each population. Each entry is formatted as follows: mutation position first line, nucleotide changes second line, and protein effect third line. Mutation positions are given as absolute genomic coordinates in the *E. coli *EcNR1 reference sequence (Additional file 2). SNPs are indicated by base transition/transversion. Small insertions are indicated by a '+', with the size (number of bp) of the insertion and sequence of inserted bases. Small deletions are designated by '-' with a format similar to that for small insertions. Ribosome binding site is abbreviated as RBS.

**Figure 4:**
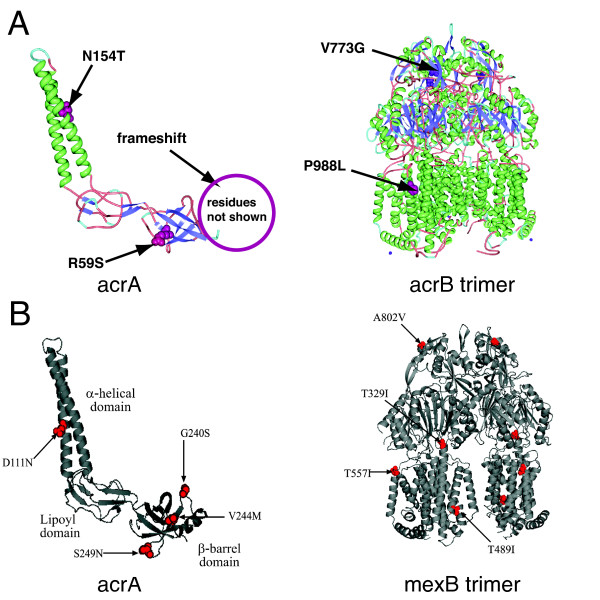
***acrAB *mutations detected in end populations**. The *acrAB *locus was sequenced in clonal isolates from each end population (G1, G2, G3, X1, X2, X3). **(A) **Discovered mutations mapped onto *acrA *and *acrB *protein structures, **(B) **Mutations in *acrA *and *mexB *(an *acrB *homolog in *Pseudomonas aerogenosa*) associated with changes in substrate specificity or *acrA-mexB *assembly.

*acrAB *mutations were discovered in X1, X2, X3, G1, and G3 populations (Table 2). Each population fixed only a single mutation in *acrA *or *acrB*, and allele frequency was near 100% (8/8 clones) except for G1, which had an allele frequency of approximately 25% (2/8 clones) and X3, with an approximate 50% allele frequency (4/8 clones). We did not detect *acrAB *mutations in the G2 population, which intriguingly had the lowest fitness out of the six endpoint populations. *tolC *mutations were not detected in any of the populations. The fixation of *acrAB *mutations in five out of six independent populations suggests strong selective pressure and parallel evolution at this locus. Mutations affected amino residues at a variety of positions in the protein structure (Figure 4A). The *acrAB *mutations acquired in the isobutanol tolerant lineages bear noteworthy similarities to mutations reported to affect substrate specificity in *acrA *and *mexB*, a *Pseudomonas aerogenosa *structural homolog to *acrB *(Figure 4B) [29]. Mutations N154T and R59S of AcrA are spatially proximal to D111N and V244M AcrA mutations reported to affect substrate specificity of AcrA-MexB (Figure 4). Mutation V773 of AcrB is in the vicinity of the TolC docking region of MexB/AcrB, where mutation A802V of MexB is known to affect substrate specificity (Figure 4). Mutation P988L of AcrB is located in a turn between transmembrane α-helices; several MexB mutations associated with changes in substrate specificity (T329I, T557I, and T489I) also occur in turns between transmembrane α-helices (Figure 4).

*marC *mutations were detected in all endpoint populations, providing strong evidence of parallel adaptation at this locus (Table 2). All detected *marC *mutations were transposon (IS1 or IS5) insertions, with the exception of an in-frame six bp deletion in G2 (Table 2). Transpositions occurred at positions 1625925 and 1626081/1626084, suggesting that these sites are insertion hotspots. Transpositions into *marC *likely cause loss-of-function from disruption, and could also affect expression of the divergently transcribed *marRAB *operon. Functional effects of the *marC *six bp deletion in G2 are not immediately obvious; this mutation results in deletion of two residues (V13 and V14) from a transmembrane helix.

Mutations in *mdh *were also common in the evolved populations, with mutations detected in X2, X3, and G3 at approximately 100% allele frequency (8/8 clones) (Table 2). All *mdh *mutations were insertions or deletions resulting in frameshifts. Since substantial numbers of amino acid residues are affected in each case, these mutations are likely to cause loss-of-function of *mdh*. *hfq *mutations were less common in the endpoint populations, with mutations detected in X1 and G3 only. The X1 Hfq mutation I24M is located in the 3'-proximal purine nucleotide selectivity pocket (R-site) [30]. The R-site is involved in binding polyA RNA, but possible functional effects of the I24M mutation are not immediately obvious [30]. In G3, the ribosome binding site of *hfq *is partially deleted, potentially leading to lower intracellular Hfq protein levels through reduced translation initiation rate of *hfq *mRNA.

### Genotypic evolutionary dynamics

**Figure 5:**
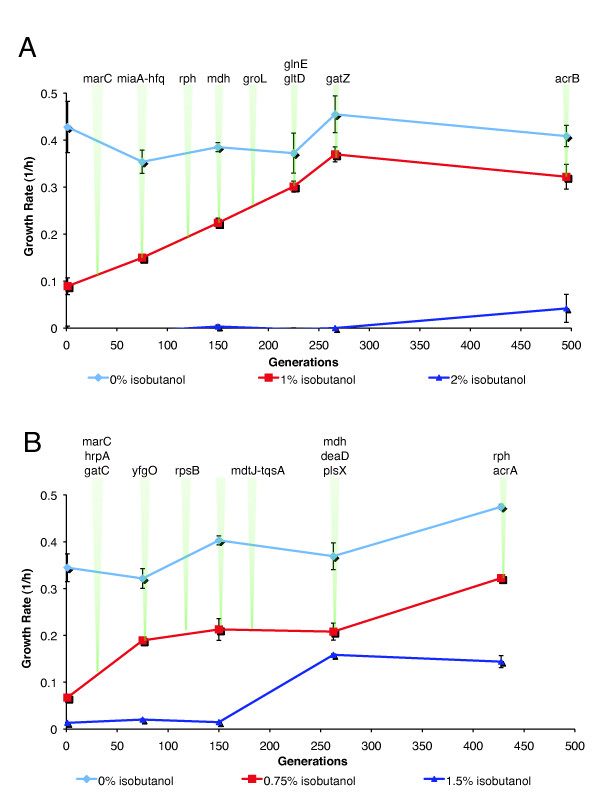
**Fitness trajectories and temporal order of mutations**. Intermediate generations of the G3 and X3 lineages were phenotyped and genotyped. Phenotyping was done by measuring growth rate at various isobutanol concentrations, while genotyping was done via allele specific PCR or direct Sanger sequencing of PCR products from whole population samples. Green arrows denote intermediate generations that were genotyped. Mutations are listed above the first generation in which they were detected. **(A) **Mutations in G3.266.7 and *gatZ *and *acrB *mutations (detected in G3.26/G3.6) were traced through various evolutionary time points. **(B) **All mutations detected in X3.5 were traced through various evolutionary time points.

We investigated the dynamics of genotypic adaptation in the G3 and X3 lineages by phenotyping and genotyping population samples from intermediate generations (Figure 5, Additional file 3). Phenotyping was done by assessing growth rate at various isobutanol concentrations, while intermediate generation genotyping was conducted by screening whole-population samples for mutations identified in sequenced clones, using Sanger sequencing of PCR amplified loci of interest or allele specific PCR. Due to the large number of mutations in the G3 end population clones, we screened only for those mutations identified in G3.266.7 and the *acrB *and *gatZ *loci (Figure 5A). All mutations detected in X3.5 were screened in the intermediate generations (Figure 5B).

The phenotype/genotype trajectories reveal that genotypic adaptations in each lineage had pleiotropic effects across different isobutanol concentrations. In both the X3 and G3 lineages, the first mutations acquired (*marC/miaA-hfq *in G3 and *marC/gatC/hrpA/yfgO *in X3) appear to drastically increase growth rates at intermediate isobutanol concentrations (1% and 0.75% w/v for G3 and X3, respectively), while having neutral or negative effects at 0% isobutanol (Figure 5). The initial *marC/miaA-hfq *mutations fixed in the G3 lineage appear to have a slightly negative effect on growth rate at 0% isobutanol (Figure 5A). Subsequent mutations in the G3 lineage (*rph, mdh, groL, glnE, gltD*, and *gatZ) *appear to monotonically increase the growth rate at 1% (w/v) isobutanol while gradually restoring growth rate at 0% isobutanol (Figure 5A). In the G3 lineage, the 0% and 1% (w/v) isobutanol growth rate trajectories appear to plateau after about 260 generations, while growth rate at 2% (w/v) isobutanol increases to the endpoint population (Figure 5A). In contrast, the growth rate trajectories at 0% and 0.75% (w/v) isobutanol in X3 increase to the end of the evolution, while the growth rate at 1.5% (w/v) isobutanol is relatively constant after generation 266 (Figure 5B). Interestingly, in X3 there was a period during the evolution between generations 150 and 266 where growth rate changes at 0% and 0.75% (w/v) isobutanol were flat, while there was a rapid increase in the growth rate at 1.5% (w/v) isobutanol. The growth rate increase in 1.5% (w/v) isobutanol is correlated with an *mdtJ::IS5::tqsA *mutation appearing at generation 180 and a *mdh/deaD/plsX *mutation cluster appearing in generation 266.

### DNA microarray study of gene expression changes in G3.2

**Figure 6:**
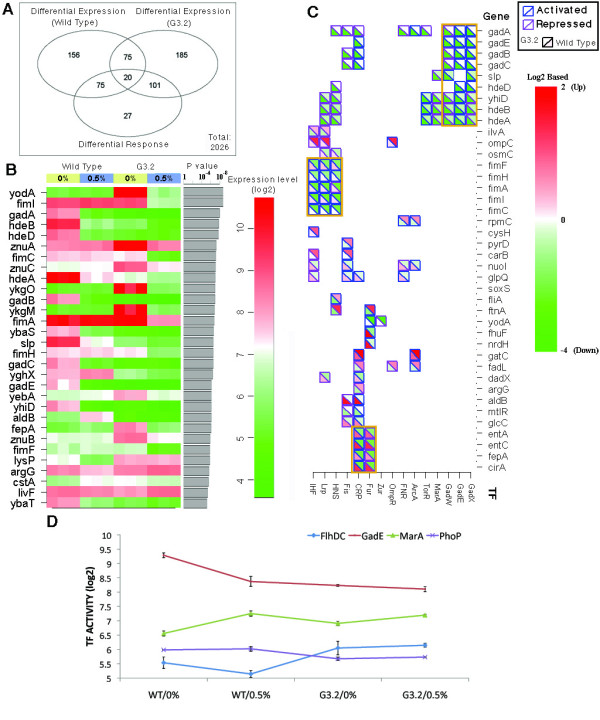
**Microarray study of gene expression changes in G3.2**. DNA microarray study was conducted to study changes in gene expression between isobutanol tolerant clone G3.2 and the parent *E. coli *EcNR1. G3.2 and *E. coli *EcNR1 (WT) were grown to mid-exponential phase in both 0% and 0.5% (w/v) isobutanol spiked minimal media. **(A) **Summary of genes expression changes. **(B) **Top 30 genes with the most significant differences in transcriptional response between G3.2 and WT. **(C) **Top 40 genes with the most significant differences in transcriptional response between G3.2 and WT, and controlling transcription factors. **(D) **Activities of transcription factors predicted by Network Component Analysis (NCA).

To gain insights into potential regulatory adaptations to isobutanol stress, we performed a gene expression study with G3.2, a highly isobutanol tolerant sequenced clone. We examined gene expression in G3.2 and the parent *E. coli *EcNR1 (WT) in 0% and 0.5% (w/v) isobutanol glucose minimal medium. For each strain/culture condition (G3.2/0% isobutanol, G3.2/0.5% isobutanol, WT/0% isobutanol, WT/0.5% isobutanol), three biological replicates were employed. Cultures were inoculated in media containing respective amounts of isobutanol, grown to mid log phase and harvested for transcriptome measurement. RNA samples were labelled and hybridized to a custom *E. coli *microarray as described in the Materials and methods section. A total of 4280 genes were included on the microarrays. After a pre-processing procedure that included background adjustment and normalization, 4235 genes with acceptable signals were subject to further analysis. Two filters were first employed to select genes with notable changes across the conditions, which resulted in a list of 2026 genes. Two-sample student's t-test was then conducted to determine statistically significant differences in gene expression. The full set of microarray results is included in Additional file 4. As illustrated in Figure 6A, 326 and 381 genes were differentially regulated by isobutanol stress in WT and G3.2 respectively. Differential transcriptional response between WT and G3.2 to isobutanol stress was observed for 223 genes, with the most significantly perturbed genes (ranked by p-value) shown in Figure 6B (see Additional file 4 for full results). Real time quantitative reverse transcription polymerase chain reaction (qRT-PCR) was used to validate two genes with large expression changes (*gadA *and *fimI*) and two genes with subtle expression changes (*fabA *and *rfaJ*) (Additional file 5). Target expression levels were determined by fitting a MAK2 model to qRT-PCR data, and expression was normalized to housekeeping gene *rpoD*, which was found to be invariant across all strains/conditions in our microarray data set and has been used in other studies to normalize gene expression data in gram negative bacteria [31,32]. Expression levels measured by qRT-PCR correlated well with microarray data (Additional file 5).

BiNGO was used to assess any overrepresented Gene Ontology (GO) in the full set of genes with differential transcriptional response, using p = 0.05 as a cutoff for significance. Overrepresented ontologies included transition metal ion transport, amine transport, amino acid metabolic processes, glutamine family amino acid metabolic processes, chemical homeostasis, and various cell envelope related components and processes (including flagella and fimbriae, polysaccharide biosynthesis, and lipid metabolism); a full list of overrepresented gene ontologies and related genes is available in Additional file 4. We further investigated changes in regulation by examining transcription factors known to control genes differentially regulated between WT and G3.2. Acid fitness island genes (*gadA, gadE, gadB, gadC, slp, hdeD, yhiD, hdeB*, and *hdeA*), regulated by GadE, GadX, and GadW, are strongly repressed at both 0% and 0.5% (w/v) isobutanol in G3.2 (Figure 6B and 6C, Additional file 4). Fimbrial biogenesis genes (*fimF, fimH, fimA, fimI, fimC*), regulated by IHF, Lrp, and HNS, are strongly repressed in G3.2 by isobutanol; genes associated with iron acquisition (*entA, entC*, *fepA*, and *cirA*), regulated by Fur and CRP, are found to be repressed by isobutanol in G3.2 as well (Figure 6B and 6C, Additional file 4).

To dissect the apparently complex regulatory changes evolved in G3.2, we applied Network Component Analysis (NCA) to the microarray data to identify transcription factors with significant activity changes in G3.2 compared to WT (Figure 6D, Additional file 4). Based on previous study of isobutanol response network in *E. coli *[7] and preliminary examination of our microarray data, we selected 16 transcription factors (TFs) that are potentially involved in isobutanol tolerance (ArcA, PdhR, Fnr, Fur, FlhDC, OmpR, CRP, GadE, MarA, Nac, LexA, PurR, Fis, IHF, PhoB and PhoP) for this analysis. Due to limited data (i.e. four strain/isobutanol conditions), we used a subset of four TFs in each NCA analysis and repeated the analysis for different combinations of TFs. Only TFs with consistent and significant predicted activity changes across different combinations of TFs and different replicates were retained for further analysis. GadE, PhoP, FlhDC, and MarA were subsequently found to be the most significantly perturbed TFs in G3.2 compared to WT (Figure 6D).

NCA reveals constitutively reduced activity in G3.2 of GadE, a regulator of the acid fitness island genes, and PhoP, a regulator of genes involved in Mg^2+ ^homeostasis, resistance to antimicrobial peptides, acid resistance (including acid fitness island genes), and LPS modification (Figure 6D). FlhDC, a master regulator of flagellum biosynthesis, has increased activity in G3.2 and is not repressed by isobutanol, as in WT (Figure 6D). MarA, which regulates genes associated with response to oxidative stress, organic solvents, and heavy metals, shows increased activity at 0% isobutanol in G3.2 relative to WT, and reduced upregulation in response to isobutanol. In a previous study of the isobutanol response network in *E. coli *[7], it was concluded that activities of ArcA, PhoB, and Fur were significantly increased by isobutanol stress due to isobutanol induced quinone/quinol malfunction. We performed NCA for various combinations of ArcA, PhoB, and Fur with FlhDC, GadE, MarA, and PhoP to determine whether these results are recapitulated in our study. We found that for most tested TF combinations, ArcA, PhoB, and Fur activities are increased by isobutanol in WT EcNR1, consistent with previous results (Additional file 4 and [7]). Responses of ArcA, PhoB, and Fur in G3.2 differ from WT, suggesting that these transcriptional responses to isobutanol stress may have changed during evolution (Additional file 4). Especially notable is the differential response of Fur to isobutanol, with upregulation in WT versus downregulation in G3.2 observed for many tested TF combinations (Additional file 4). Many of the top differentially expressed genes identified in our microarray study are regulated by IHF, HNS, Fis, and CRP (which were incidentally identified as being significantly perturbed by isobutanol in [7]), suggesting that these TFs may also be involved in the differential transcriptional response between WT and G3.2 (Figure 6C).

Integrated examination of genotype and microarray expression data yields insights into the genetic basis of gene expression and transcription factor activity patterns in G3.2. One of the first mutations fixed in the G3 lineage is *miaA-hfq *4407505 -7:AGGAAAA, a partial ribosome binding site deletion that is likely to reduce *hfq *mRNA translation. *hfq *is a global regulator that functions by mediating binding between a variety of sRNAs and their target mRNAs, which can alter target protein levels via effects on translation initiation or mRNA degradation [33]. *hfq *is required for translation of *rpoS *(σ38) mRNA, the master transcriptional regulator for general stress response; thus G3.2 is expected to have lower RpoS activity [33]. Previous work indicates that in minimal medium, *flhDC *is strongly repressed by RpoS, while *gadE *is strongly upregulated by RpoS; the activity changes observed for these transcription factors are consistent with reduced RpoS activity in G3.2 [34]. Many other gene expression changes in G3.2 are also consistent with reduced RpoS activity (see Additional file 4). In addition to *rpoS, hfq *regulates numerous other genes involved in a variety of cellular processes, however since *hfq *regulation is post-transcriptional, many of these effects cannot be captured in a DNA microarray study [33]. Besides possible changes in post-transcriptional regulation, microarray data indicates that *rpoS *is differentially regulated at the transcriptional level in G3.2 compared to WT. *rpoS *is upregulated in WT by isobutanol stress, consistent with previous gene expression studies [7] (Additional file 4). In contrast, in G3.2 *rpoS *expression appears to be slightly repressed by isobutanol; furthermore the basal expression level of *rpoS *in G3.2 is lower compared to WT, providing additional evidence of reduced RpoS activity in G3.2.

NCA analysis revealed constitutively reduced activity of the PhoP and GadE transcriptional regulators. PhoP is part of a Mg^2+ ^responsive two-component signal transduction system, with sensor kinase PhoQ phosphorlyating (and thus activating) PhoP in response to low Mg^2+ ^levels [35,36]. Interestingly, G3.2 has a *phoQ *1197581 A→G mutation, causing L209P in transmembrane region 2 in the PhoQ protein, which may lead to reduced activity of the PhoPQ system. Transcriptional changes caused by *phoPQ *perturbation are potentially adaptive, since PhoP is involved in stress response and regulates genes related to Mg^2+ ^homeostasis, resistance to antimicrobial peptides, acid resistance, and LPS modification [35,36]. In a previous NCA study [7], GadE activity was found to be strongly repressed by isobutanol. This finding was recapitulated in our NCA results for WT, while in G3.2 GadE is constitutively repressed (Figure 6D). The evolution of constitutive GadE repression in G3.2 hints that the GadE regulon (comprised of the major acid resistance genes) may be maladaptive to isobutanol stress. There is substantial overlap between the PhoP, GadE, Hfq, and RpoS regulons, pointing towards possible co-evolution between these different regulators.

### Investigating phenotypic and functional effects of mutations

**Figure 7:**
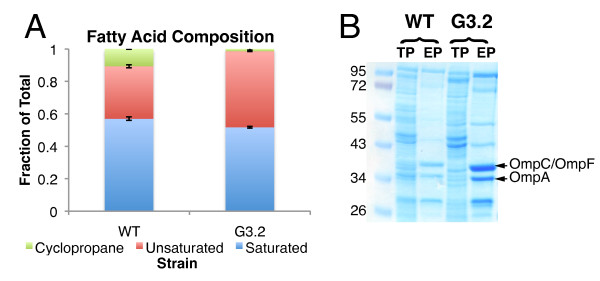
**Cell envelope composition of G3.2 compared to WT EcNR1**. Possible cell envelope adaptations in G3.2 were investigated by profiling cellular fatty acid composition and cell envelope proteins in G3.2 and the parent *E. coli *EcNR1 during growth at 0.5% (w/v) isobutanol. **(A) **Fatty acid composition of G3.2 and WT EcNR1. Relative proportions of cyclopropane, unsaturated, and saturated fatty acids were determined by GC-FID analysis. **(B) **SDS-PAGE profile of cell envelope proteins in G3.2 and WT EcNR1. Cell envelopes were isolated from 5 × 10^9 ^cells and analyzed by SDS-PAGE. For comparison, 20 μg total cellular proteins (TP) from each strain (WT and G3.2) were analyzed alongside the isolated cell envelopes (EP). As a reference, bands corresponding to outer membrane proteins OmpA and OmpC/OmpF are indicated with arrows. Experiment was repeated to verify results (not shown).

Previous investigations have identified the cell envelope as a primary target of solvent toxicity. G3.2 contains mutations in numerous genes and regulators associated with the cell envelope, including *secA *and *lepB *(components of the Sec apparatus, which translocates periplasmic and membrane targeted proteins from the cytosol), *hfq *(involved in sRNA mediated regulation of many membrane proteins), *fepE *and *yjgQ *(involved in LPS biosynthesis), and *phoPQ *(regulator of various LPS modification genes). Additionally, the DNA microarray study revealed that many genes related to cell envelope components and processes were differentially expressed in G3.2. We investigated possible cell envelope adaptations by profiling cellular fatty acid composition and cell envelope proteins in the parent *E. coli *EcNR1 strain and G3.2 during growth at 0.5% isobutanol (Figure 7A and 7B). Cellular fatty acid composition was determined using gas chromatography-flame ionization detector (GC-FID) quantification, and was found to differ considerably between G3.2 and WT EcNR1 (Figure 7A). The cyclopropane fatty acid fraction is significantly reduced in G3.2, probably as a result of downregulation of *cfa *(cyclopropane fatty acyl phospholipid synthase) in this strain (Figure 7A; see Additional file 4 for *cfa *expression data from the DNA microarray study). Additionally, the overall unsaturated:saturated fatty acid ratio is increased in G3.2 (Figure 7A), due mainly to an increase in the proportion of C16:1 and C18:1 fatty acids relative to C16:0 (data not shown).

To determine cell envelope protein profiles, cell envelopes were isolated from 5 × 10^9 ^cells by sonication and differential centrifugation and then analyzed with sodium dodecyl sulfate polyacrylamide gel electrophoresis (SDS-PAGE) (Figure 7B). SDS-PAGE analysis reveals an overall increase in cell envelope proteins (on a per cell basis) in G3.2 compared to WT. (Figure 7B). To examine changes in relative protein abundance between G3.2 and WT, protein bands were quantified by densitometry analysis (using ImageJ software) and normalized to the sum of intensities of the major protein bands. The 72 kDa, 55 kDa, and OmpC/OmpF bands were found to be notably upregulated in G3.2 relative to WT, with relative increases of 1.2, 2.2, and 1.3 fold, respectively (Figure 7B). Upregulation of OmpC/OmpF is consistent with DNA microarray results, which show upregulation of *ompF *in G3.2 (Additional file 4).

In addition to characterizing possible cell envelope adaptations in G3.2, we conducted detailed investigations of phenotypic and functional effects of key mutations identified in isobutanol tolerant clones. Selected mutations were reconstructed in *E. coli *EcHW24 (EcNR1 Δ*mutS*) singly and in various combinations using ssDNA mediated recombination [37]. We focused on characterizing parallel genotypic adaptations, including *marC*, *acrAB, mdh*, and *rph *mutations identified in G3.2 and X3.5, as well as the first four mutations to appear in the G3 lineage, *marC*, *miaA-hfq, rph, mdh*, and *groL*, which are associated with monotonically increasing isobutanol tolerance (Figure 4A). *marC*, *acrAB, mdh*, and *rph *single mutants were constructed to study the phenotypic and functional effects of these mutations in isolation, while *marC*, *miaA-hfq, rph, mdh*, and *groL *mutations were constructed singly and in various combinations to study fitness benefits and investigate possible epistatic interactions among these mutations. Phenotypic effects were investigated by measuring growth of mutants in isobutanol spiked minimal medium, and functional assays were performed for *acrAB *and *mdh*. The parent *E. coli *EcNR1 and *E. coli *EcNR1 single gene knockouts (Δ*acrA::kan*, Δ*acrB::kan*, Δ*mdh::kan*) were employed as controls in phenotype and functional assays.

*marC *mutations were detected in every evolution endpoint population. All detected *marC *mutations were transposon (IS1 or IS5) insertions, with the exception of an in-frame six bp deletion in G2. *marC *transposon insertions could not be produced with ssDNA mutagenesis, so we instead approximated the effect of transposon insertions by knocking out *marC*, reasoning that this could mimic effects of gene disruption caused by transposon insertion; additionally, deletion of *marC *was found to improve isobutanol tolerance in an independent study (James C. Liao, UCLA personal communications). Consistent with our expectations, Δ*marC::kan *was found to significantly improve maximum specific growth rates and final densities in 0.5% (w/v) isobutanol minimal medium relative to the parent *E. coli *EcNR1 (Table 3). Growth rate improvement of Δ*marC::kan *was higher in xylose medium (39 ± 2% above WT growth rate) compared to glucose medium (20 ± 5% above WT growth rate) at 0.5% (w/v) isobutanol (Table 3). In contrast, Δ*marC::kan *improved final cell densities more in glucose medium compared to xylose medium (40 ± 10% vs. 7.6 ± 0.5% improvement over WT; Table 3). Δ*marC::kan *had a slight negative effect on maximum specific growth rate and final cell densities at 0% (w/v) isobutanol in both xylose and glucose media (Table 3).

**Table 3:** Phenotypic and functional effects of selected *marC*, *acrAB*, *mdh*, and *rph *mutations

			0% i-BtOH		0.5% i-BtOH		
				
Locus	Gene/mutation	Clone	**Δμ**_**max**_	**ΔOD**_**max**_	**Δμ**_**max**_	**ΔOD**_**max**_	Functional Effect
***marC***	*ΔmarC::kan (xylose media)*	X1,X2,X3 (IS1/IS5 insertion)	-5.0 ± 0.5%	-2.4 ± 0.1%	39 ± 2%	7.6 ± 0.5%	-
	*ΔmarC::kan (glucose media)*	G1,G3 (IS1 insertion)	-3.2 ± 0.2%	-7.8 ± 0.3%	20 ± 5%	40 ± 10%	-

***acrAB***	*acrA *483735 +1:A	X3.5	14 ± 1%	3.3 ± 0.1%	49 ± 9%	72.4 ± 0.8%	Reduced EtBR efflux; 188 ± 7% increase in intracellular EtBr
	Δ*acrA::kan*	N/A (control)	6.5 ± 0.6%	7.6 ± 0.5%	32 ± 5%	103 ± 1%	Reduced EtBR efflux; 210 ± 10% increase in intracellular EtBr
	*acrB *480665 G→A	G3.2	11.2 ± 0.9%	2.1 ± 0.1%	22 ± 3%	31 ± 1%	Reduced EtBR efflux; 21 ± 8% increase in intracellular EtBr
	Δ*acrB::kan*	N/A (control)	5.7 ± 0.6%	-3.6 ± 0.1%	8.2 ± 1.1%	64 ± 2%	Reduced EtBR efflux; 340 ± 20% increase in intracellular EtBr

***mdh***	*mdh *3390936 +5:AACCT	X3.5	-3.7 ± 0.3%	-1 ± 0.1%	0.4 ± 0.1%	13 ± 2%	Loss of function; no detectable mdh activity
	*mdh *3390726 -1:C	G3.2	2.3 ± 0.1%	4.4 ± 0.1%	-8.1 ± 3%	-1.2 ± 0.1%	Loss of function; no detectable mdh activity
	Δ*mdh::kan*	N/A (control)	-1.6 ± 0.2%	4 ± 0.1%	10.8 ± 1.6%	10 ± 1%	Loss of function; no detectable mdh activity

***rph***	*rph *3823220 +4:GTCG	G3.2	39 ± 2%	11.9 ± 0.1%	49 ± 16%	-12.9 ± 0.1%	-

Selected point mutations in *acrAB*, *mdh*, and *rph *identified in isobutanol tolerant clones were reconstructed in the parent *E. coli *EcNR1 strain using ssDNA mediated mutagenesis. *marC *transposon insertions could not be produced with ssDNA mutagenesis; instead, we approximated the effect of transposon insertions by knocking out *marC*. Reconstructed mutants were phenotyped by measuring specific growth rate in minimal media with 0% and 0.5% (w/v) isobutanol; percent change in growth rate and maximum OD_600 _relative to the parent *E. coli *EcNR1 are reported. Functional effects of *acrAB *mutations were assessed with an *in vivo *ethidium bromide accumulation assay and *mdh *mutations were assessed by directly measuring malate dehydrogenase enzyme activity in cell lysates.

*acrAB *mutations were identified in five out of six independent evolved populations, suggesting that mutations at this locus are likely to have positive adaptive effects. Consistent with this expectation, *acrA *483735 +1:A (identified in X3.5) and *acrB *480665 G→A (identified in the G3 lineage) dramatically increased maximum specific growth rates and final cell densities in 0.5% (w/v) isobutanol minimal medium relative to the parent *E. coli *EcNR1, while having more subtle effects on growth in 0% isobutanol (Table 3). Δ*acrA::kan *and Δ*acrB::kan *produced fitness benefits of similar or greater magnitude, implying that loss-of-function of *acrAB *is associated with improved isobutanol tolerance (Table 3). This result is surprising given that the AcrAB-TolC efflux pump is an important mechanism of tolerance to other organic solvents and antibiotics. AcrAB-TolC efflux pump activity was measured via ethidium bromide (EtBr) accumulation in reconstructed single mutants and clonal isolates harbouring *acrAB *mutations from evolution end populations [38]. Since AcrAB-TolC is the primary efflux pump for EtBr, mutations altering AcrAB-TolC activity or substrate specificity would be expected to affect the accumulation of intracellular EtBr [38]. Increased EtBr accumulation (consistent with reduced AcrAB-TolC activity) was observed in all examined end population clonal isolates harbouring *acrAB *mutations (full data set in Additional file 6). The *acrA *483735 +1:A single mutant had an EtBr accumulation profile similar to Δ*acrA::kan *and X3.5. In contrast, the EtBr accumulation profile in G3.2 was similar to Δ*acrB::kan*, but *acrB *480665 G→A (identified in the G3.2) showed only modest changes in EtBR accumulation relative to the parent strain, implying that G3.2 may have additional mutations affecting efflux pump activity.

The AcrAB-TolC multidrug efflux pump has been well characterized in its role for antibiotic and solvent tolerance, but a recent study suggests that AcrAB-TolC may also function as an exporter for a hitherto unidentified quorum sensing signal (QSS) [39]. There is strong evidence that the QSS exported by AcrAB-TolC is associated with upregulation of *rpoS *transcription; Δ*acrAB *mutants have reduced *rpoS *expression and altered temporal patterns of expression [39]. Our gene expression study of G3.2 provides evidence of reduced RpoS activity in this strain. Interestingly, two evolved populations, X1 and G3, were found to have mutations in *hfq*, which is required for translation of *rpoS *mRNA, suggesting that RpoS modulation might be a common adaptive effect of these different mutations. We assayed RpoS activity via iodine staining in the parent *E. coli *EcNR1 strain, each evolution endpoint population (G1, G2, G3, X1, X2, X3), a Δ*acrA::kan *mutant, and a constructed single mutant containing the *miaA-hfq *mutation found in the G3 lineage (*miaA-hfq *4407505 -7:AGGAAAA). RpoS positively regulates glycogen biosynthesis, which can be measured by staining cells with iodine - cells with higher glycogen levels stain darker [40]. While this assay is an indirect measure of RpoS activity and is subject to many confounding factors (such as other regulation of glycogen biosynthesis), it is commonly used in literature and has been demonstrated to be well correlated with RpoS activity [40].

**Figure 8:**
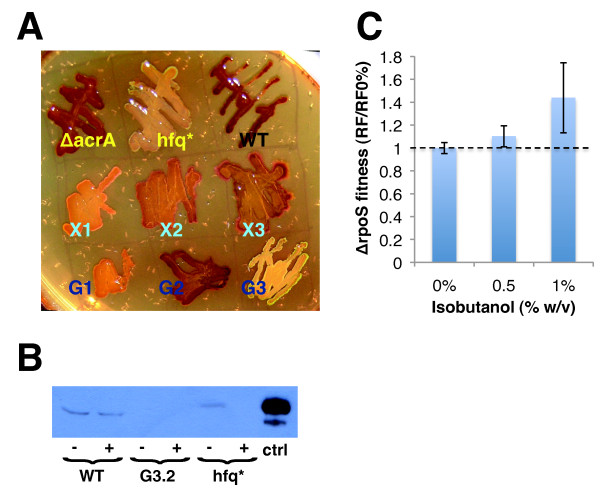
**Survey of RpoS activity in evolved populations and phenotype study of a Δ*rpoS *mutant**. RpoS activity was assayed in evolved populations and selected single mutants using an I_2 _staining assay and Western blot analysis. **(A) **I_2 _staining assay. Overnight cultures were streaked on glucose minimal medium agar spiked with 0.35% (w/v) isobutanol, incubated at 30°C for 48 hours, and then stained with USP tincture of iodine. Samples are as follows (from top left, to bottom right): *E. coli *EcNR1 Δ*acrA::kan *(ΔacrA), *E. coli *EcHW24 *miaA-hfq *4407505 -7:AGGAAAA (hfq*), *E. coli *EcNR1 (WT), Xylose #1 end population (X1), Xylose #2 end population (X2), Xylose #3 end population (X3), Glucose #1 end population (G1), Glucose #2 end population (G2), Glucose #3 end population (G3). **(B) **Western blot analysis of RpoS in total cellular protein extracted from cultures of *E. coli *EcNR1 (WT), G3.2, and *E. coli *EcHW24 *miaA-hfq *4407505 -7:AGGAAAA (hfq*) grown to early exponential phase either with (+) or without (-) 0.5% (w/v) isobutanol in NG50 medium. Experiment was repeated several times to verify results; representative Western blot shown. Purified *E. coli *RpoS (NeoClone) was used as a positive control (ctrl). **(C) **Phenotype study of a Δ*rpoS *mutant. *E. coli *BW25113 Δ*rpoS::kan *(obtained from the Keio collection [82]; strain # JW5437-1) and parent strain *E. coli *BW25113 were grown in 0%, 0.5%, and 1% (w/v) isobutanol glucose media. To facilitate comparison, we report normalized relative fitness (RF/RF0%), defined as relative fitness divided by relative fitness at 0% (w/v) isobutanol; relative fitness (RF) was calculated as μ_ΔrpoS_/μ_WT _where μ_ΔrpoS _is the maximum specific growth rate (h^-1^) of *E. coli *BW25113 Δ*rpoS::kan *and μ_WT _is the maximum specific growth rate (h^-1^) of *E. coli *BW25113.

Iodine staining results are show in Figure 8A. Single mutant *miaA-hfq *4407505 -7:AGGAAAA (hfq* in Figure 8A) stains lighter than the parent *E. coli *EcNR1 strain (WT in Figure 8A), consistent with the expected reduction of Hfq and RpoS activity in this mutant. Both of the end populations harbouring *hfq *mutations, X1 and G3, stain much lighter than WT suggesting reduced Hfq activity and subsequently RpoS levels in both end populations (Figure 8A). G1 and X2 also show significantly lighter staining than WT, suggesting reduced RpoS activity in these strains as well (Figure 8A). Staining in X3 is only slightly lighter than WT, while G2 and Δ*acrA::kan *(unexpectedly) stain very similarly to WT. Curiously, the association between Δ*acrAB *and reduced RpoS reported in the literature was not evidenced in our iodine staining assay. We suspect that this discrepancy may be due to differences in assay techniques. Previous studies of RpoS activity of Δ*acrAB *mutants were done with liquid cultures, with RpoS activity assayed by real-time PCR or Western blotting, while our assay was done on solid medium using iodine staining to measure intracellular glycogen levels, which are directly controlled by RpoS [39]. Concentrations of the QSS exported by AcrAB-TolC are likely to vary dramatically between liquid and solid cultures due to cell density differences, and could thus confound assay results.

As a follow up to the I_2 _staining assay, we directly checked RpoS expression by Western blot analysis of RpoS in the parent EcNR1 strain (WT), G3.2, and single mutant *miaA-hfq *4407505 -7:AGGAAAA (hfq*) grown with and without 0.5% (w/v) isobutanol (Figure 8B). RpoS Western blot analysis was repeated several times to verify results; Figure 8B shows a representative Western blot. RpoS expression is evident in the parent EcNR1 strain (WT) at both 0% and 0.5% (w/v) isobutanol, while RpoS expression was not detected in G3.2 under either condition (Figure 8B). Interestingly, in hfq* RpoS is detectable at 0% isobutanol, but not at 0.5% (w/v) isobutanol. These results directly demonstrate reduced RpoS expression at 0.5% (w/v) isobutanol in G3.2 and the *miaA-hfq *single mutant relative to the parent EcNR1 strain, consistent with I_2 _staining results (Figure 8A). We attempted Western blot analysis of RpoS in other strains (including evolution endpoint populations G1, G2, G3, X1, X2, and X3; sequenced isobutanol tolerant clone X3.5; and Δ*acrA::kan *single mutant); however, due to inconsistent outcomes between experiments, we are unable to draw conclusions about RpoS expression levels in these other strains (results not shown). To ascertain whether reduced RpoS activity is indeed adaptive to isobutanol stress, we examined the isobutanol tolerance phenotype of a Δ*rpoS::kan *mutant (Figure 8C). Δ*rpoS::kan *caused a growth defect at 0% and 0.5% (w/v) isobutanol relative to the WT strain, while at 1% (w/v) isobutanol the relative fitness of Δ*rpoS::kan *is slightly higher than WT; to facilitate comparison, we report normalized relative fitness (calculated by dividing relative fitness by relative fitness at 0% w/v isobutanol). These results suggest that attenuated RpoS activity may indeed be adaptive to isobutanol stress, since normalized relative fitness is increased at 0.5% and 1% (w/v) isobutanol (Figure 8C). However, complete loss-of-function of *rpoS *appears to incur significant costs that overshadow adaptive effects at isobutanol concentrations below 1% (w/v) (Figure 8C).

*mdh *mutations appear in three out of six evolution end populations, suggesting that these mutations may be adaptive. However, in 0% and 0.5% (w/v) isobutanol spiked minimal medium, we found relatively minor differences in growth between the parent *E. coli *EcNR1 strain, *mdh *3390726 -1:C (found in G3.2) single mutant, *mdh *3390936 +5:AACCT (found in X3.5) single mutant, and Δ*mdh::kan *(Table 3). Thus *mdh *mutations do not appear to improve isobutanol tolerance in isolation, hinting that fitness benefits may come via epistatic interactions with other mutations. All of the *mdh *mutations identified in evolution end populations were indels causing frameshifts, suggesting that these mutations lead to loss-of-function. To assess functional effects of *mdh *mutations, NADH dependent malate dehydrogenase activity was measured in crude cell lysates of G3.2, *mdh *3390726 -1:C single mutant, X3.5, *mdh *3390936 +5:AACCT single mutant, Δ*mdh::kan*, and the parent *E. coli *EcNR1. NADH dependent malate dehydrogenase activity was not detectable in G3.2, *mdh *3390726 -1:C single mutant, X3.5, *mdh *3390936 +5:AACCT single mutant, or Δ*mdh::kan*, while assay of the parent *E. coli *EcNR1 yielded enzyme activity of 3.8 ± 0.2 U/mg-wet-cells, consistent with our expectation that 3390726 -1:C and 3390936 +5:AACCT lead to loss-of-function of *mdh.*

Restorative mutations are commonly observed in *rph-pyrE *during experimental evolution studies with *E. coli *K12 MG1655 [17], and indeed all sequenced clonal isolates from our evolution end populations had *rph *mutations. We investigated the adaptive benefits of the *rph *3823220 +4:GTCG mutation acquired in the G3 lineage. This mutation was found to substantially improve maximum specific growth rate in both 0% and 0.5% (w/v) isobutanol spiked glucose minimal medium, consistent with the notion that *rph *mutations are a general adaptation to growth on minimal media (Table 3).

**Figure 9:**
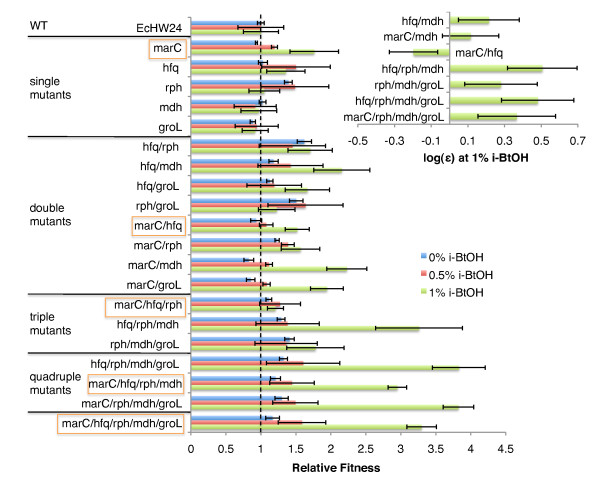
**Fitness effects of first five mutations fixed in G3 lineage**. The first five mutations fixed in the G3 lineage (*marC*, *miaA-hfq*, *rph*, *mdh*, and *groL*) were constructed singly and in various combinations in *E. coli *EcHW24 (EcNR1 Δ*mutS*) using ssDNA mediated recombination; the *marC::IS1 *mutation was approximated by knocking out *marC*. The constructed mutants and *E. coli *EcHW24 were phenotyped by measuring the maximum specific growth rate in 0%, 0.5%, and 1% (w/v) isobutanol spiked minimal glucose media; relative fitness was calculated as mutant maximum specific growth rate (h^-1^) divided by *E. coli *EcHW24 maximum specific growth rate (h^-1^). Mutation combinations corresponding to the order of appearance in G3 are highlighted. Epitasis is assumed to follow a simple multiplicative fitness model , where w = relative fitness of a particular mutation combination, ε = total epistatic interaction parameter, and w_i _= relative fitness of single mutants; log epistasis is calculated as . Inset shows log(ε) at 1% (w/v) isobutanol for selected mutation combinations with notable epistasis.

Genotypic adaptation to isobutanol stress is complex and involves diverse genetic loci, as revealed in our genome resequencing results. The apparent multigenic nature of isobutanol tolerance suggests that epistasis, interactions between different genes, is probably an important factor in many of the evolved genetic adaptations. To study fitness benefits and investigate possible epistasis, the first five mutations fixed in the G3 lineage (Figure 5A), *marC*, *miaA-hfq, rph, mdh*, and *groL*, were reconstructed singly and in various combinations in *E. coli *EcHW24, using multiplex recursive ssDNA mediated mutagenesis [37]. As explained above, the *marC::IS1 *mutation could not be created using ssDNA recombination, so instead we knocked out *marC *(*marC::kan*) to approximate gene disruption effects caused by IS1 insertion. The resulting mutant set was phenotyped by measuring the maximum specific growth rate in 0%, 0.5%, and 1% (w/v) isobutanol glucose minimal media; results are presented as relative fitness, defined as mutant maximum specific growth rate divided by maximum specific growth rate of the parent *E. coli *EcHW24. Epitasis is assumed to follow a simple multiplicative fitness model , where w = relative fitness of a particular mutation combination, ε = total epistatic interaction parameter, and w_i _= relative fitness of single mutants; log epistasis is calculated as  (Figure 9) [41].

As would be expected, there is a general trend of improved relative fitness with increasing numbers of mutations (Figure 9; *miaA-hfq *abbreviated *hfq *and *marC::kan *abbreviated *marC*). Relative fitness improvements at 1% (w/v) isobutanol are the most dramatic, with the *miaA-hfq/rph/mdh/groL *and *marC*/*rph/mdh/groL *quadruple mutants having a 3.8 fold increase in growth rate compared to *E. coli *EcHW24; fitness changes at 0% and 0.5% (w/v) isobutanol are much smaller, and appear to plateau with introduction of an *rph *mutation (Figure 9). Individually, the *marC*, *miaA-hfq, rph, mdh*, and *groL *mutations have relatively modest effects. *mdh *and *groL *single mutants have fitness essentially identical to the parent *E. coli *EcHW24 at all tested isobutanol concentrations (Figure 9). The *rph *single mutant has improved relative fitness at 0% and 0.5% isobutanol, while the *marC *and *miaA-hfq *single mutants have improved relative fitness at 0.5% and 1% isobutanol (Figure 9). Notable improvements in relative fitness in 1% (w/v) isobutanol were observed for some double mutants, in particular, *miaA-hfq/rph, miaA-hfq/mdh, marC/mdh*, and *miaA-hfq/groL *(Figure 9). We suspect that there may be positive epistasis between *miaA-hfq *and each of *mdh*, *rph*, and *groL*; however due to limited growth rate measurement precision, we can assert statistically significant epistasis only for *miaA-hfq *and *mdh *(Figure 9). Likewise, positive epistasis between *marC *and each of *mdh *and *groL *seems plausible (Figure 9), but cannot be ascertained due to limited measurement precision. Interestingly, *marC *and *miaA-hfq *demonstrate significant negative epistasis at 1% isobutanol (Figure 9).

Many higher order mutation combinations show substantial relative fitness improvements at 1% (w/v) isobutanol (Figure 9). The quadruple mutants *miaA-hfq/rph/mdh/groL *and *marC*/*rph/mdh/groL *have the greatest relative fitness, with a 3.8 fold improvement in growth rate, followed by the *marC*/*miaA-hfq/rph/mdh/groL *pentuple mutant and *hfq/rph/mdh *triple mutant, each having a 3.3 fold improvement in growth rate (Figure 9). Results for the reconstructed *marC*/*hfq/rph/mdh *quadruple mutant and full pentuple mutant are approximately consistent with the evolution trajectory (Figure 5A). Epistasis analysis reveals significant positive epistatic interactions for *miaA-hfq/rph/mdh, rph/mdh/groL, miaA-hfq/rph/mdh/groL*, and *marC/rph/mdh/groL *(Figure 9). Comparison of fitness effects of *rph/mdh/groL *vs. *miaA-hfq/rph/mdh/groL *and *marC/rph/mdh/groL *provides compelling evidence that an *miaA-hfq *or *marC *genetic background exhibits positive epistasis with *rph/mdh/groL *(Figure 9).

## Discussion

Due to broad mechanisms of toxicity, microbial solvent tolerance is a complex phenotype, involving adaptations in diverse cellular processes [6]. This inherent complexity suggests that genotypic adaptation to solvent stress will involve a rugged fitness landscape with many epistatic interactions between genes [42]. Fitness landscape topology and epistasis have important ramifications for efforts towards engineering complex phenotypes. Many of the approaches previously employed to investigate genetic bases of adaptation to solvent stress are inherently limited to exploring restricted regions of the fitness landscape and often fail to capture interactions between distal genes; thus these approaches may fail to uncover many important adaptations [6]. In our study, we used experimental evolution of multiple lineages of *E. coli *under isobutanol stress followed by genome resequencing and phenotypic characterization, allowing us to investigate the full bases of adaptation in our evolved lineages. Our results reveal many novel patterns of genotypic adaptation and suggest several important tolerance mechanisms, informing future efforts towards engineering more robust strains of *E. coli *for isobutanol production and also providing general insights into the evolution of complex stress tolerance phenotypes.

### Genotypic patterns of adaptation: epistasis, global effect mutations, and parallel evolution

Consistent with the complex nature of solvent tolerance, our genome resequencing results reveal genetic adaptations in a diversity of cellular processes (Table 3). The apparent multigenic nature of isobutanol tolerance suggests that epistatic interactions and coevolution between different genetic loci are probably important factors in many of the evolved adaptations. Examining the genotypes of X3.5 and G3.2 reveals several possible examples of epistasis and coevolution between genes that encode interacting proteins or that participate in functionally related cellular processes. G3.2 has mutations in *secA *and *lepB*, two components of the Sec protein translocation apparatus that exports periplasmic and membrane proteins from the cytosol (Table 1, Figure 3) [43]. The SecA S233P mutation is in the preprotein binding domain of SecA, which recognizes and binds nascent cytosolic peptides targeted for export, while *lepB *is a signal peptidase that cleaves the N-terminal leader peptide proteins after secretion; these mutations may collectively alter the peptide specificity of the Sec complex [43,44]. Both clones sequenced from the G3 lineage (G3.2 and G3.266.7) have a probable loss-of-function mutation (indel leading to frameshift) in *gltD*, a subunit of glutamate synthase, and *glnE*, a regulator of glutamine synthase activity; these mutations suggest a rewiring of nitrogen metabolism towards increased glutamine synthesis [45]. The X3 lineage acquired mutations in *rpsB*, the 30S ribosomal subunit S2, and *deaD *(*csdA*), an RNA helicase involved in ribosome biogenesis that is known to be a multicopy suppressor of temperature-sensitive *rpsB *mutants [46]. The *rpsB *mutation, which occurs before *deaD *in the X3 lineage, is associated with improved growth at 0% and 0.75% (w/v) isobutanol, while the appearance of a *mdh/deaD/plsX *mutation cluster is associated with improved growth at 1.5% (w/v) isobutanol (Figure 5B). Possible functional effects of the *rpsB/deaD *mutations are not clear. However, recent studies have identified alterations in rRNA processing that contribute to acid tolerance in *Clostridium acetobutylicum *and mutations in ribosomal machinery in *Pseudomonas putida *that contribute to chemical tolerance, setting a precedent for ribosomal mechanisms of complex stress tolerance [47,48].

Epistasis can also occur though more cryptic mechanisms. The evolution trajectory (Figure 5A) showed that fitness at 1% (w/v) isobutanol increases monotonically as *marC*, *miaA-hfq, rph, mdh*, and *groL *were acquired in the G3 lineage, yet *rph, mdh*, and *groL *single mutants did not have significant fitness effects at 1% (w/v) isobutanol (Figure 9). Our mutation reconstruction analysis demonstrates that significant positive epistasis is correlated with an *miaA-hfq *or *marC *genetic background (Figure 9). Curiously, we detected significant negative epistasis between *miaA-hfq *and *marC*. Negative epistasis often occurs between genes with overlapping functions [49], suggesting that *miaA-hfq *and *marC *could have a shared mechanism for improving isobutanol tolerance; this interpretation is further supported by the fact that *miaA-hfq *and *marC *each show positive epistasis with subsequent mutations in the G3 lineage (ie *rph/mdh/groL*). Since *marC *is a poorly characterized gene of unknown function, possible mechanistic links between *miaA-hfq *and *marC *or between *marC *and *rph/mdh/groL *are not apparent. One possibility is that *marC *mutations (such as deletion or transposon insertions) could affect expression of the divergently transcribed *marRAB *locus, which is involved in regulation of genes associated with response to oxidative stress, organic solvents, and heavy metals; some of the genes in the *marRAB *regulon are coregulated by *hfq*, including *acrAB *[50]. Indeed, our gene expression study of G3.2 reveals slightly reduced levels of *marA *and *marB *transcripts, and NCA identified transcription factor MarA as having significantly perturbed activity (Figure 6D and Additional file 4). An independent study of isobutanol tolerance in *E. coli *reported that fitness benefits of Δ*marRAB *and Δ*marC *were comparable, but deletion of the full *marCRAB *locus yielded the greatest improvement in isobutanol tolerance (James C. Liao, UCLA personal communications). More investigation and characterization of *marC *is needed to elucidate mechanisms underlying the observed negative epistasis between *miaA-hfq *and *marC *and positive epistasis between *marC *and *rph/mdh/groL*.

The functional basis of epistasis between *rph/mdh/groL *and *miaA-hfq *is also not immediately obvious, as *hfq, rph, mdh*, and *groL *participate in seemingly disparate cellular processes: *hfq *is a global regulator that mediates binding between sRNAs and their target mRNAs, *rph *has RNase PH activity, *mdh *is TCA cycle enzyme malate dehydrogenase, and *groL *is part of the groEL chaperone [45]. *miaA-hfq *4407505 -7:AGGAAAA is the second mutation acquired in the G3 lineage, and is associated with significantly improved fitness at 1% isobutanol both in the evolutionary trajectory (Figure 5A) and as a reconstructed single mutant (Figure 9). The mutation *miaA-hfq *4407505 -7:AGGAAAA is a partial ribosome binding site deletion that is likely to reduce translation initiation rate of *hfq *mRNA (evaluated with the Ribosome Binding Site Calculator, Beta version) and thus reduce Hfq protein levels (evidenced by iodine staining assay, Figure 8A) [51]. Since Hfq is a global regulator, a reduction in activity is likely to perturb expression of many proteins. The net effect of these perturbations is clearly beneficial at 1% (w/v) isobutanol, but some of the specific expression perturbations caused by reduced Hfq are likely to be maladaptive. This suggests that some of the subsequent mutations in G3 may be compensatory to the perturbations caused by reduced Hfq activity, and that the initial fixation of *hfq *in G3 may have been a crucial determinant of the evolutionary trajectory in this lineage. Interestingly, many of the mutations subsequently acquired in the G3 lineage are in genes known to be regulated (either directly or indirectly) by Hfq, including *acrB, phoPQ, gltD, mdh, groL*, and the *gat *operon [33,45]. For example, reduced Hfq activity is associated with increased *mdh, gltD*, and *phoPQ *expression; mutations in these genes could thus be compensatory by reducing expression or protein activity [33,45]. Indeed, the *mdh *mutation in G3 was verified to be a loss-of-function mutation, and our gene expression study indicates reduced PhoPQ activity in G3.2, probably due to the *phoQ *mutation. Reduced Hfq activity is also associated with reduced GroL levels and the acquired *groL *mutation could likewise be compensatory, perhaps through increasing GroL activity [33].

The role of the *miaA-hfq *mutation in the evolution of isobutanol tolerance in G3 suggests that global regulatory network perturbation is an important genetic mechanism of adaptation. Indeed, an accumulating body of research points towards regulatory network perturbation as a general and important mechanism of adaptive evolution under a variety of contexts and selective pressures [22,52,53]. Investigations of transcription factor network evolution suggest higher evolvability and rates of divergence for central transcription factors compared to peripheral regulators [54]. Studies of short-term experimental evolution of *E. coli *on glycerol and lactate media, as well as long-term evolution on glucose medium, have revealed that mutations affecting genes with global regulatory functions (including global transcription factors, *hfq*, and genes controlling DNA supercoiling) often provide large fitness benefits and constitute an important mode of adaptation [16,17,20,53]. A recent study involving evolution of ethanol tolerance in *E. coli *further supports this notion; a mutation in the global regulator *rho *was found to improve ethanol tolerance in an evolved strain [10]. Furthermore, the concept of regulatory network perturbation as a mode of adaptation has been utilized for engineering complex stress tolerance phenotypes; targeted mutagenesis of *rpoD *and *rpoA *in *E. coli *was used to generate mutant libraries with perturbed global gene expression patterns, from which variants with dramatically improved ethanol tolerance (*rpoD *mutagenesis) and n-butanol tolerance (*rpoA *mutagenesis) were iteratively isolated [13,14,55].

Many of the adaptive global effect perturbations identified in previous studies involved transcriptional regulatory changes, often through mutations in transcription factors or genes controlling DNA supercoiling. Indeed, our gene expression study of G3.2 revealed a number of transcriptomic adaptations, probably due in part to changes in RpoS and PhoP activity. However, our results also suggest that global changes in post-transcriptional regulation might constitute important modes of adaptation in our evolved lineages. Gene Ontology analysis of mutations accumulated in G3.2 and X3.5 indicates a significant overrepresentation of genes with RNA helicase activity, including *secA *(G3.2), *rhlB *(G3.2; see Additional file 1), *hrpA *(X3.5), and *deaD *(X3.5). RNA helicases can participate in various modes of post-transcriptional regulation, including mRNA processing, translation, or degradation [56]. Regulatory effects related to RNA helicase activity of *secA *(G3.2), *rhlB *(G3.2), *hrpA *(X3.5), and *deaD *(X3.5) are not well characterized, and thus it is difficult to speculate about specific mechanisms of adaptation related to these mutations; however acquisition of *hrpA *and a *mdh/deaD/plsX *mutation cluster in the X3 lineage is correlated with significant improvements in isobutanol tolerance (Figure 5B). These results suggest that RNA helicases may be interesting targets for targeted mutagenesis to improve isobutanol tolerance and possibly other complex stress tolerance phenotypes.

In addition to mutations in RNA helicase genes, we discovered other possible post-transcriptional regulatory adaptations in our evolved lineages. X3.5 acquired a mutation in *rpsB*, ribosomal subunit S2, which could potentially affect translation; however other effects are possible, as noted in our epistasis discussion. The X1 and G3 lineages acquired mutations in post-transcriptional regulator *hfq*, which probably result in reduced activity, as evidence by iodine staining assay (Figure 8A). Hfq is a global regulator that functions by mediating binding between a variety of sRNAs and their target mRNAs, which can alter target protein levels via effects on translation initiation or mRNA degradation [33]. Interestingly, many stress response regulons incorporate sRNA mediated regulation, including the RpoS regulated general stress response (*rpoS *translation is mediated through *rprA *and *dsrA *sRNAs), oxidative and antibiotic stress (*oxyS, gcvA*, and *micF *sRNAs), osmotic shock (*omrA *and *omrB *sRNAs), cell envelope stress (*micA *and *rybB *sRNAs), and iron limitation (*ryhB *sRNA) [57]. Stress response tuning via *hfq *mutations, possibly dominated by modulation of RpoS, may thus constitute a mechanism of adaptation to isobutanol stress, but due to the global regulatory role of Hfq, many other adaptive effects are possible. Beneficial *hfq *mutations have recently been discovered in *E. coli *lineages evolved on lactate minimal medium, under glucose limitation, and under phosphate limitation, underscoring that *hfq *evolution represents a flexible and general mechanism of adaptation, and *hfq *may be an interesting mutagenesis target for engineering improved stress tolerance phenotypes [17,58,59].

The role of centrality in the evolution of biochemical networks has been investigated in a number of studies. Investigations of protein-protein interaction and metabolic networks suggest that central proteins tend to evolve more slowly than peripheral proteins, in contrast to findings for transcriptional regulatory networks [54,60,61]. Intriguingly, we found numerous potentially adaptive mutations in genes known to have high centrality in protein-protein interaction and metabolic networks. Examples of adaptation at central protein-protein interaction nodes include adaptive mutations in *groL*, a chaperone involved in folding of many different proteins, and mutations in *secA*/*lepB*, components of the Sec complex which exports numerous proteins out of the cytosol. We also discovered potentially adaptive mutations in high centrality metabolic network nodes, such as *mdh *and *gltD/glnE*. Our results suggest that more investigation is warranted into the role of centrality in the evolution of biochemical networks.

Parallel evolution occurs when independent lineages evolve similar traits, and is considered strong evidence of selective pressure [22]. We have identified several instances of parallel genotypic adaptation in our evolved lineages. In particular, mutations in *rph, gatYZABCD *operon, *mdh, acrAB*, and *marC *were found in all of the resequenced evolution endpoint clones (Table 2). *marC *mutations (consisting mostly of transposon insertions) were discovered in all six evolution endpoint populations, while deletion of a genomic region containing *marC *(*hipA*-*flxA*) was reported in an independent study of evolution of isobutanol tolerance (Table 2) [21]. Loss-of-function of *marC *appears to be broadly adaptive to isobutanol stress under various growth conditions, including glucose and xylose minimal media (Table 3) and yeast extract supplemented glucose media [21]. In addition to *marC*, *acrAB *mutations were also prevalent in evolution endpoint populations (occurring in five out of six populations; Table 2), and adaptive *acrAB *mutations have also been reported in other investigations of isobutanol tolerance [11,21]. In both our work and independent studies, it was found that loss-of-function of *acrAB *was correlated with significantly improved isobutanol tolerance. Like *marC*, effects of *acrAB *mutations appear to be broadly adaptive since isobutanol tolerance is improved for a variety of growth conditions, including xylose and glucose minimal media (this study), yeast extract supplemented glucose media [21], and rich LB media [11].

In contrast to *marC *and *acrAB *mutations, in isolation *mdh *mutations did not improve isobutanol tolerance. Yet the appearance of *mdh *indel mutations in three out of six independent lineages strongly suggests adaptive effects, perhaps through epistatic interactions. In the case of the G3 lineage, we find significant positive epistasis between *hfq *and *mdh *(Figure 9). However, *mdh *mutations also appear in strains without *hfq *mutations or evidence of altered Hfq activity, suggesting that *mdh *mutations may be epistatic with other genetic backgrounds. Phenotype analysis of reconstructed mutants hints that there may be functional overlap between *hfq *and *marC*, and we observe possible epistasis in a constructed *marC*/*mdh *double mutant, although the measured epistasis is not statistically significant (Figure 9); given these results and the prevalence of *marC *mutations in the evolution endpoint populations, epistasis between *marC *and *mdh *seems plausible but more investigation is needed.

We did not investigate *rph *and *gatYZABCD *parallel evolution as thoroughly as the mutations discussed above. As described previously, restorative mutations in *rph *are general adaptations of *E. coli *K12 MG1655 to growth in minimal medium rather than specific adaptations to isobutanol stress. However, it should be noted that *rph *mutations have been reported to be epistatic with a variety of genetic backgrounds, and our mutation reconstruction analysis points towards such a possibility (Figure 9). The *gatYZABCD *operon is strongly upregulated in response to isobutanol stress (Figure 6 and Additional file 4). *gatYZABCD *genes are involved in galactitol transport and catabolism, and do not have an obvious role in isobutanol tolerance. An independent study found that deletion of *gatY *was correlated with improved isobutanol tolerance [21]. We suspect that *gatYZABCD *overexpression in response to isobutanol is spurious and provides no stress tolerance benefit, perhaps leading to selective pressure for mutations that reduce expression levels or lead to loss-of-function. We did not investigate *gatYZABCD *in our other evolved lineages due to the relatively large size of this locus.

During the course of our isobutanol tolerance evolution study, we became aware of a similar project concurrently underway in another laboratory, which was published while this manuscript was in revision [21]. It is informative to compare the findings reported in [21] with our own results. In this parallel study, *E. coli *JCL260 (an isobutanol production strain) was evolved on yeast extract - glucose media supplemented with isobutanol and then sequenced [21]. A total of 27 mutations were identified in SA481 (the sequenced evolved isolate), consisting of 25 transposon (IS10) insertions, one SNP, and one large genomic deletion [21]. Mutation repair analysis and subsequent gene deletion studies revealed five key genetic loci involved in isobutanol tolerance: *tnaA*, *gatY*, *acrA*, *yhbJ*, and the *hipA*-*flxA *genomic region [21]. It was demonstrated that deletion of these genetic loci and of *marCRAB *(contained within the *hipA*-*flxA *genomic region) conferred isobutanol tolerance [21]. In our study, we discovered parallel mutations in *acrAB*, *marC*, and the *gatYZABCD *operon, and demonstrated that deletion of *acrA*, *acrB*, and *marC *conferred isobutanol tolerance, consistent with the independently reported results [21]. Furthermore, in [21] single mutations reportedly had minor impacts on isobutanol tolerance (with the exception of Δ*acrA*), but mutation combinations (Δ*acrA*/Δ*gatY*, Δ*acrA*/Δ*tnaA*, Δ*tnaA*/Δ*gatY*/Δ*acrA*, Δ*tnaA*/Δ*gatY*/Δ*acrA/*Δ*marCRAB*, and Δ*tnaA*/Δ*gatY*/Δ*acrA/*Δ*marCRAB/*Δ*yhbJ*) showed synergistic effects on isobutanol tolerance; these findings are consistent with our suggestion that epistasis is an important factor in the evolution of isobutanol tolerance and possibly other complex stress tolerance phenotypes.

Beyond the five key mutations discussed in [21], there are other notable parallels and relationships between the mutations in SA481 and those identified in our study. We identified parallel loss-of-function mutations in malate dehydrogenase *mdh *(catalyzing NAD dependent oxidation of malate to oxaloacetate) in three out of six evolution endpoint populations (Table 3), and our results indicate that *mdh *mutations provide fitness benefits via epistasis with *hfq *and possibly *marC *mutations (Figure 9). SA481 contains an IS10 insertion in malate dehydrogenase *maeA *(catalyzing NAD dependent decarboxylation of malate to pyruvate) [21]; the preponderance of malate dehydrogenase mutations in isobutanol tolerant mutants suggests that rewiring of metabolic pathways around the malate node may be important in the evolution of isobutanol tolerance. Mutations affecting the *mdtJI-tqsA *locus were identified both in our study (*mdtJ::IS5::tqsA *in X3.5) and in SA481 (*tqsA::IS10*), suggesting that this locus may also be involved in isobutanol tolerance [21]. Finally, we demonstrated that RpoS is downregulated in G3.2 and identified mutations in RpoS regulators (including *hfq *and *acrAB*). Interestingly, in SA481 the RpoS regulator *rssB *(which regulates proteolytic degradation of RpoS) is mutated, as well as *acrA*, providing additional evidence that RpoS modulation may be adaptive to isobutanol stress.

While there are many overlaps between our results and those reported in [21], there are also significant differences between the genetic loci identified in these two studies. *tnaA *(L-cysteine desulfhydrase/tryptophanase) and *yhbJ *(which senses glucosamine-6-phosphate and regulates glucosamine-6-phosphate synthase *glmS*) were reported to contribute to isobutanol tolerance in [21], but were not identified in our study. Likewise, we discovered epistatic mutations in *miaA-hfq*, *mdh*, *rph*, and *groL *that confer high isobutanol tolerance in glucose minimal media, yet none of these genes were identified in [21]. Since evolved adaptations often show tradeoffs in relative fitness across different environments, differences in conditions between these two studies could account for different evolutionary trajectories. In particular, it should be noted that in our study we used a different parent strain (*E. coli *EcNR1 vs. JCL260 in [21]) and different media (glucose or xylose minimal media vs. yeast extract supplemented media in [21]); we also note that adaptations in our evolved strains exhibit antagonistic pleiotropy in rich media (Figure 2A and 2B).

### Remodeling the cell envelope: possible mechanism of adaptation to isobutanol stress

**Figure 10:**
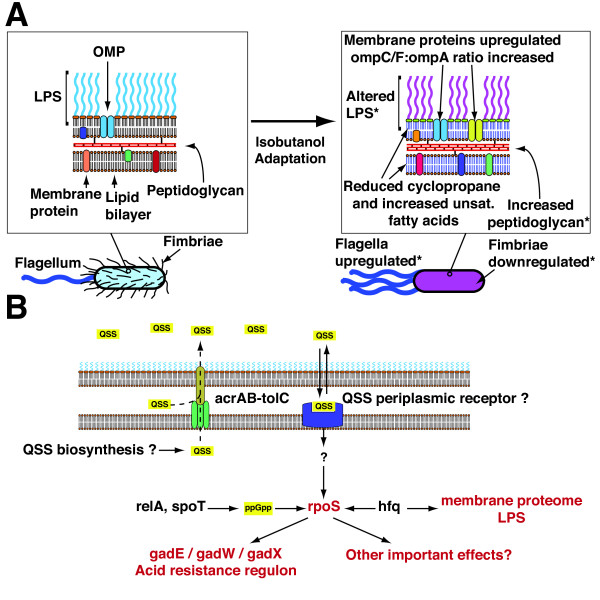
**Possible mechanisms of evolved isobutanol tolerance**. Examining genotypic adaptations to isobutanol stress in the context of known modes of solvent toxicity suggests that remodeling the cell envelope and stress response attenuation might be important mechanisms of adaptation in our evolved lineages. **(A) **Postulated mechanisms of adaptation involving the cell envelope. Asterisked (*) items were inferred from genoptyic data without direct experimental validation. We observe adaptations that may lead to alterations in cell envelope protein composition, downregulation of fimbriae biogenesis and upregulation of flagellar biogenesis, and alterations in peptidoglycan, membrane lipid composition, and lipopolysaccharide (LPS) composition. Collectively, these adaptations suggest that evolution may be remodeling the cell envelope to counteract the detrimental effects of isobutanol on the cell membrane. **(B) **Our results suggest that attenuation of RpoS activity may be a convergent adaptive effect associated with *hfq, acrAB-tolC, relA*, and *spoT *mutations. Hfq is required for translation of *rpoS *mRNA, RelA and SpoT synthesize signalling molecule ppGpp which upregulates RpoS, and one study suggests that AcrAB-TolC exports an unidentified quorum sensing signal (QSS) that upregulates RpoS, possibly via a periplasmic receptor [33,39,74]. All depicted regulatory interactions involve upregulation. Signalling molecules (ppGpp and QSS) are boxed in yellow. Effects/gene targets that might be ultimately linked to isobutanol tolerance are red. AcrAB-TolC quorum sensing model adapted from [39], using similar notation.

An accumulated body of evidence indicates that the cell membrane is a primary target of alcohol toxicity. Alcohols have been demonstrated to intercalate the membrane lipid bilayer, leading to detrimental changes in the physicochemical properties of membrane [6]. Examining the genotypic and phenotypic adaptations of our evolved lineages in the context of this known mechanism of toxicity reveals a trend of evolution targeting various features of the cell envelope through a diversity of processes. We observe adaptations that may lead to alterations in cell envelope protein composition, downregulation of fimbriae biogenesis and upregulation of flagellar biogenesis, and alterations in peptidoglycan, membrane lipid composition, and lipopolysaccharide (LPS) composition (Figure 10A). Collectively, these adaptations suggest that evolution may be remodeling the cell envelope to counteract the detrimental effects of isobutanol on the cell membrane.

Examination of the genotypes of G3.2, G3.6, and X3.5 yields evidence of significant selective pressure on membrane proteins; Gene Ontology analysis indicates a significant overrepresentation of membrane proteins among mutated genes (corrected p-value = 7.23 × 10^-3^) [28]. We have also identified various mutations that could cause global changes in cell envelope proteome composition. Such changes are potentially adaptive to isobutanol stress, perhaps countering effects of isobutanol on membrane integrity and mechanical properties. Two lineages, X1 and G3, acquired *hfq *mutations that appear to reduce activity, and in G3, the *miaA-hfq *mutation was shown to improve tolerance at 1% (w/v) isobutanol (Table 3 and Figure 9). Many important membrane proteins, including *ompA, ompC, ompW, ompF, ompT, lamB, cirA, fecA*, and *fepA *feature Hfq mediated sRNA regulation; indeed, almost half of the Hfq binding sRNAs with known targets regulate the expression of outer membrane proteins [57,62]. In most cases sRNAs are involved in downregulation, with sRNAs binding to target mRNA (mediated by Hfq) leading to translation inhibition and/or increase mRNA degradation [57]. Thus mutants with reduced Hfq activity would be expected to have altered membrane protein composition and a general increase in outer membrane proteins, which has been verified in other studies and found to cause pleiotropic phenotype effects [33,57,58,63]. Consistent with this expectation, 1-D SDS-PAGE characterization of cell envelope protein composition of G3.2 revealed an overall increase in membrane proteins in G3.2 compared to WT (Figure 7B). Additionally, several protein bands (corresponding to 72 kDa, 55 kDa, and the OmpF/OmpC bands) appear to be upregulated in G3.2 (Figure 7B). *ompF *is negatively regulated by *micF *sRNA, which both inhibits translation of *ompF *and reduces *ompF *mRNA levels. *ompF *mRNA was found to be upregulated in the DNA microarray study (Additional file 4) and SDS-PAGE analysis (Figure 7B) supports increased levels of OmpF protein, consistent with the notion that reduced Hfq activity would increase both *ompF *mRNA and OmpF protein levels by abolishing *micF *regulation. OmpC may also be upregulated in G3.2 (Figure 7B; due to the similar molecular weights of OmpF and OmpC, we were not able to resolve these proteins separately with SDS-PAGE). Interestingly, a recent study reported that a mutant deficient in *rybB *sRNA, which downregulates OmpC and OmpW, had improved SDS tolerance providing additional precedent for stress tolerance mechanisms involving modulation of sRNA mediated outer membrane protein regulation [64].

In G3.2 and G3.6, we identified mutations in *secA/lepB*, two components of the Sec protein translocation apparatus. The Sec apparatus is responsible for translocating many periplasmic and membrane targeted proteins from the cytosol; examples of known Sec substrates include MalE, LamB, OmpA, OmpF, OppA, PhoE, MBP, DegP, FhuA, FkpA, OmpT, OmpX, TolB, TolC, YbgF, YcgK, YgiW and YncE [65]. As discussed previously, the *secA/lepB *mutations may alter the peptide binding specificity of the Sec apparatus, and thus membrane and periplasmic protein composition could be altered by changes in Sec mediated translocation, perhaps by increasing export of some proteins while reducing export of others. However, we cannot predict how the S233P mutation in the preprotein binding domain of SecA will affect peptide specificity, and thus more investigation into this hypothesis is needed. Interestingly the signal recognition particle (SRP), which is involved in cotranslational targeting of peptides to the Sec translocase, has been implicated in mechanisms of acid tolerance in *Clostridium acetobutylicum *and *Steptococcus mutans *[6,47,66].

In addition to adaptations involving general modulation of membrane and periplasmic protein composition, we observe adaptations that appear to specifically target fimbriae and flagellar biogenesis (Figure 10A). G3.2 shows strong transcriptional upregulation of flagellar biosynthesis genes, and NCA identified increased activity of related transcription factor FlhDC (Figure 6D). In contrast, gene expression studies of an ethanol adapted strain showed downregulation of flagellar biosynthesis [67]. Whether upregulation of flagellar biosynthesis in G3.2 is indeed adaptive or simply a spurious consequence of reduced RpoS activity in this strain needs to be investigated. In contrast to flagellar genes, G3.2 shows strong transcriptional downregulation of the *fim *operon (*fimAICDFGH*), responsible for fimbriae biogenesis (Figure 6C). A study of ethanol tolerance with gene overexpression and transposon mutagenesis libraries demonstrated that loss-of-function of *fim *genes was correlated with improved ethanol tolerance, while *fim *overexpression was correlated with negative fitness effects [10]. These results strongly suggest that *fimAICDFGH *downregulation observed in G3.2 is indeed adaptive. Interestingly, the X3 lineage acquires a mutation in *hrpA*, a gene known to be involved in processing the *daa *fimbriae operon in pathogenic *E. coli *strains [68]. The *marC*/*gatC/hrpA *mutations occur early in the X3 lineage and are correlated with a significant fitness increase (Figure 5B). However, *E. coli *EcNR1 does not have the *daa *operon, so the role of *hrpA *in fimbrial biogenesis in this strain is not clear, and additional studies indicate that *hrpA *may have other important *in vivo *functions [69].

Modification of cell envelope peptidoglycan, lipoprotein, and LPS content is a commonly observed adaptation of bacteria to solvent stress [6]. LPS adaptations that reduce solvent accessibility to the cell membrane have been reported in literature [70]. For example, an *E. coli *strain tolerant to hydrophobic organic solvents was reported to show increased LPS content, which makes the cell surface more hydrophilic [70]. Solvent tolerant *E. coli *strains were also reported to have increased lipoprotein content, possibly strengthening the cell membrane against the fluidizing effects of solvent [70]. Enhanced peptidoglycan biosynthesis has been implicated in tolerance to ethanol and isobutanol, possibly by changing the rigidity or structural strength of the cell [10,21]. A study of ethanol tolerance with gene overexpression and transposon mutagenesis libraries demonstrated that loss-of-function of peptidoglycan biosynthesis *mur *genes was correlated with reduced fitness, while overexpression of *mur *genes was correlated with improved tolerance [10]. An independent study of isobutanol tolerance using experimental evolution and genome resequencing demonstrated that upregulation of *glmS*, which is responsible for the synthesis of peptidogylcan and LPS precursor glucosamine-6-phosphate (GlcN-6-P), resulted in improved isobutanol tolerance [21].

Our evolved lineages show several possible mechanisms of adaptation involving peptidoglycan, lipoprotein, and LPS biosynthesis. G3.2 was found to contain mutations in *fepE *and *yjgQ*, which are involved in LPS biosynthesis (Additional file 1). The G3 lineage also acquired probable loss-of-function mutations in *glnE *and *gltD*, correlated with improved fitness, that might alter nitrogen metabolism towards increased biosynthesis of glutamine, a precursor for GlcN-6-P (Figure 5A) [45]. Interestingly, our gene expression study of G3.2 also shows significant downregulation of glutamine degrading gene *ybaS*, further suggesting increased glutamine production in G3.2 (Additional file 4) [45]. These changes suggest that G3.2 may be increasing glutamine flux for peptidoglycan and LPS biosynthesis, but glutamine is a central metabolite so numerous other adaptive effects would also be possible [45]. Another adaptation potentially related to regulation of LPS biosynthesis and modification was observed in phosphoethanolamine transferase *eptB*. This gene is negatively regulated by sRNA *mgrR*, and thus *eptB *might be upregulated in X1 and G3, which harbour *hfq *mutations associated with reduced activity; additionally, *eptB *transcriptional upregulation was observed in the G3.2 gene expression study (Additional file 4) [36]. *eptB *modifies the LPS by adding a phosphoethanolamine (pEtN) moiety to the terminal 3-deoxy-d-*manno*-octulosonic acid (KDO) of LPS [36]. Possible adaptive effects of this modification to isobutanol stress are not clear, but interestingly *eptB *upregulation is associated with increased resistance to polymyxin B, a detergent-like antibiotic that targets the cell membrane [36].

Our gene expression study of G3.2 reveals differential transcriptional regulation for multiple genes involved in peptidoglycan, lipoprotein, and LPS biosynthesis. Many genes associated with LPS biosynthesis are differentially expressed in G3.2; Gene Ontology analysis revealed significant overrepresentation of cellular polysaccharide biosynthetic process genes (corrected p-value = 3 × 10^-2^). NCA identified significantly reduced activity of PhoP and GadE transcription factors, and incidentally many of the differentially regulated peptidoglycan, lipoprotein, and LPS genes are part of these regulons (Figure 6 and Additional file 4). We observed downregulation of *slp *(starvation lipoprotein, acid resistance regulon), *pagP *(palmitoyl transferase for lipid A, PhoP regulon), y*bjG *(undecaprenyl pyrophosphate phosphatase, PhoP regulon), and *slyB *(outer membrane lipoprotein, PhoP regulon) (see Additional file 4); downregulation of these genes may be adaptive to isobutanol tolerance, but the collective effect of these perturbations is unclear. Other potentially important lipoprotein and LPS genes differentially regulated in G3.2 included *nlpD *(putative outer membrane lipoprotein, downregulated in G3.2) and numerous members of the *rfa *gene cluster, which comprise the pathway for LPS core-oligosacchride assembly (*rfaL, rfaQ, rfaG, rfaS, rfaB, rfaI, rfaJ, rfaY*, and *rfaZ*, all upregulated) (Additional file 4).

Bacteria are known to adapt to solvent stress by altering membrane lipid composition, including cis-to-trans isomerisation of fatty acids, changing the proportions of saturated/unsaturated fatty acids, altering composition of phospholipid head groups, and altering fatty acid acyl chain length [6]. In our evolved lineages, we observe several possible adaptations to isobutanol stress involving membrane lipid composition. The X3 lineage acquires a mutation in *plsX *that may be adaptive to isobutanol stress (Figure 5B). The function of *plsX *has not been fully elucidated, but it is suspected to play a role in fatty acid metabolism, possibly regulating the intracellular concentration of acyl-[acyl carrier protein] (acyl-ACP) [71]. Many genes associated with lipid metabolism are differentially expressed in G3.2. Gene Ontology analysis revealed significant overrepresentation of lipid catabolic process genes (corrected p-value = 4.6 × 10^-2^); in particular many lipid catabolism genes were downregulated in G3.2, including *tesB *(acyl-CoA thioesterase II), *hdhA *(7-alpha-hydroxysteroid dehydrogenase), *gabT *(4-aminobutyrate aminotransferase), *pgpB *(phosphatidylglycerophosphatase B), *fadJ *(fused enoyl-CoA hydratase and epimerase and isomerase), *fadE *(acyl coenzyme A dehydrogenase), and *fadB *(3-hydroxyacyl-CoA dehydrogenase). Other notable expression changes in lipid metabolism genes include downregulation of *cfa *(cyclopropane fatty acyl phospholipid synthase), *ybhO (*cardiolipin synthase 2), and *aidB *(predicted acyl-CoA dehydrogenase), and upregulation of *fabA *(3-hydroxydecanoyl-ACP dehydrase) (Additional file 4). We profiled the fatty acid composition of G3.2, revealing a significant decrease in cyclopropane fatty acids and increased unsaturated fatty acids relative to WT (Figure 7A). The decrease in cyclopropane fatty acids is attributable to downregulation of *cfa*, while the increase in unsaturated fatty acids is probably a collective effect of multiple gene expression changes (with *fabA *upregulation possibly playing an important role) [72]. Previous studies have demonstrated that *E. coli *responds to short chain (C2-C4) n-alkanol exposure by increasing the unsaturated:saturated fatty acids ratio, suggesting that the increase in unsaturated fatty acids in G3.2 is indeed adaptive to isobutanol stress [6]. On the other hand, the decrease in cyclopropane fatty acids observed in G3.2 is somewhat counterintuitive, since cyclopropane fatty acids have been implicated in tolerance to n-butanol [6,73]; further investigation will be needed to determine whether decreased cyclopropane fatty acids are adaptive to isobutanol stress.

Collectively, we have observed many genotypic and gene expression changes that suggest evolution may be remodeling the cell envelope to counteract the detrimental effects of isobutanol on the cell membrane. However, further investigation is needed to profile cell envelope changes in isobutanol tolerant strains and to ascertain that the observed changes are indeed adaptive to isobutanol stress. Analysis of cell envelope protein and fatty acid composition (Figure 8) will need to be extended to other isobutanol tolerant lineages to investigate convergent adaptations; additionally, the cell envelope proteome could be resolved in greater detail by performing two-dimensional electrophoresis. Peptidoglycan and LPS composition will need to be profiled across different isobutanol tolerant lineages to validate inferences drawn from the gene expression and genotype data and to investigate convergent adaptations. In addition to profiling cell envelope composition in evolved isolates, mutations in genes related to cell envelope composition (e.g. *hfq, secA/lepB, glnE/gltD, phoQ, plsX*, etc.) could be reconstructed singly and in combinations with other mutations from their respective lineages, and the resulting mutant library could be profiled. These investigations will help to elucidate causal links between genotype, isobutanol tolerance, and cell envelope composition.

### Stress response attenuation: surprising adaptations

We observe evidence of RpoS downregulation in many of our evolved isobutanol tolerant lineages, implying that reduction of RpoS activity may be adaptive to isobutanol stress (Figure 8A and 8B). This is a surprising result, given that RpoS is a master regulator of the general stress response and many prior studies show that reduction of RpoS activity increases sensitivity to a variety of environmental stresses [40]. In the G3 evolution endpoint population, reduced activity of Hfq (which is required for translation of *rpoS *mRNA) is probably the primary mechanism of RpoS downregulation (Figure 8A and 8B). The X1 population also contains an *hfq *mutation and shows evidence of reduced RpoS activity as well (Figure 8A). *acrAB-tolC *mutations associated with reduction or loss-of-function were common in our evolution endpoint populations, being discovered in five out of six populations total (Table 2); additionally, adaptive *acrAB *mutations were reported in an independent genomic investigation of isobutanol tolerance [21]. A recent study provides evidence that AcrAB-TolC exports an unidentified quorum sensing signal (QSS) that is associated with transcriptional upregulation of *rpoS*, possibly via a periplasmic receptor (Figure 10B) [39]. This suggests that adaptive effects associated with reduced AcrAB-TolC activity (Table 3) may be linked to downregulation of RpoS. Interestingly, a transposon mutagenesis study demonstrated that loss of function of *relA *or *spoT *is correlated with enhanced tolerance to n-butanol and isobutanol [11]. *relA *and *spoT *both synthesize guanosine tetraphosphate (ppGpp), an alarmone involved in stringent response [74]. ppGpp upregulates *rpoS*, and many genes of the RpoS regulon require ppGpp for transcription; thus the correlation of reduced *relA *or *spoT *activity with improved n-butanol and isobutanol tolerance may also be related to downregulation of RpoS activity [74].

Our results suggest that attenuation of RpoS activity may be a convergent adaptive effect associated with *hfq, acrAB-tolC, relA*, and *spoT *mutations (summarized in Figure 10B). To determine whether RpoS attenuation is indeed adaptive, we examined the isobutanol tolerance phenotype of a Δ*rpoS::kan *single mutant (Figure 8C). Δ*rpoS::kan *was found to cause a growth defect at 0% and 0.5% (w/v) isobutanol relative to the WT strain, while at 1% (w/v) isobutanol the relative fitness of Δ*rpoS::kan *is slightly higher than WT; to facilitate comparison, we report normalized relative fitness (calculated by dividing relative fitness by relative fitness at 0% w/v isobutanol). These results suggest that RpoS attenuation may indeed be adaptive to isobutanol stress. However, complete loss-of-function of *rpoS *appears to incur significant costs that overshadow adaptive effects at isobutanol concentrations below 1% (w/v) (Figure 8C), indicating that RpoS activity may need to be finely tuned to achieve optimal isobutanol tolerance. The growth defect of Δ*rpoS::kan *at 0% (w/v) isobutanol was unexpected, since a previous study examining *E. coli *K12 MG1655 and *E. coli *K12 MG1655 Δ*rpoS *reported nearly identical growth phenotypes for the parent and mutant strain in glucose minimal medium [34]; reasons for the discrepancy between our results and those reported in [34] might include differences in media formulation and genetic background of the host strains (*E. coli *K12 BW25113 in our study vs. *E. coli *K12 MG1655 in [34]).

Since RpoS is a global regulator affecting the expression of many genes, it is difficult to speculate on the specific adaptive effects of reduced RpoS activity. It is likely that only a subset of the gene expression changes elicited by reduced RpoS activity are adaptive, and certain gene expression changes may in fact be maladaptive, as suggested by the fitness costs apparent in the Δ*rpoS::kan *single mutant. In minimal medium, RpoS has been demonstrated to be a dominant activator of acid resistance genes (GadE/GadX/GadW regulon) and repressor of flagellar genes (FlhDC regulon) [34]. In G3.2, we observe gene expression changes in these regulons consistent with reduced RpoS activity, and NCA identified GadE and FlhDC among the most significantly perturbed transcription factors (Figure 6 and Additional file 4). A recent genomic study of ethanol tolerance demonstrated that overexpression of GadE/GadX/GadW regulon and *cadAB *acid resistance genes decreased ethanol tolerance, while transposon mutagenesis leading to loss-of-function of these genes was associated with increased fitness [10]. This strongly suggests that downregulation of the GadE/GadX/GadW regulon observed in G3.2 is in fact beneficial for isobutanol stress, and indeed GadE/GadX/GadW downregulation might be an important adaptive effect provided by reduced RpoS activity (Figure 10B). Given the dominance of RpoS in regulating GadE/GadX/GadW, we would expect GadE/GadX/GadW downregulation to be recapitulated in other populations with reduced RpoS activity [34]. Interestingly, G3.2 also has a *cadA *4363790 A→G mutation, further suggesting that acid resistance genes are under selective pressure (Table 1). It is not clear why attenuation of acid stress response would be adaptive to isobutanol stress; more investigation into the relationship between the GadE/GadX/GadW regulon and alcohol stress will be needed.

### Caveats and limitations

Given that evolved adaptations can be highly specific to a particular environmental context or genetic background, microbial tolerance to biofuel products should be evolved under conditions that closely approximate those used for biofuel production. We want to point out that we used aerobic cultivation conditions in our isobutanol tolerance evolution study, while isobutanol production in engineered *E. coli *strains is optimal under microaerobic conditions [3]. Additionally, we evolved a WT *E. coli *strain for tolerance to exogenous isobutanol, whereas isobutanol will be formed endogenously during production with engineered *E. coli *strains. Nonetheless, we feel our results still provide a valuable advancement in understanding mechanisms of isobutanol tolerance, and also provide interesting insights into the evolution of complex stress tolerance phenotypes. We have identified several mutations that appear to provide broad fitness benefits in a variety of conditions and genetic contexts (such as *marC *and *acrAB*, which were also identified in [21]), and it seems plausible that some of the adaptations we identified will be beneficial under microaerobic conditions and/or for endogenous isobutanol production as well.

## Conclusions

We used experimental evolution of *E. coli *followed by genome resequencing and a gene expression study to elucidate genetic mechanisms of adaptation to isobutanol stress. Comparison between strains evolved in glucose and xylose minimal media revealed little carbon source specificity of adaptation, but we find that adaptations exhibit significant antagonistic pleiotropy between rich and minimal media. By examining genotypic adaptation in multiple independent lineages, we find evidence of parallel evolution in *marC*, *hfq*, *mdh*, *acrAB, gatYZABCD*, and *rph*. Many isobutanol tolerant lineages show reduced RpoS activity, perhaps related to mutations in *hfq *or *acrAB*. Consistent with the complex, multigenic nature of solvent tolerance, we observe adaptations in a diversity of cellular processes. Many of the adaptations appear to involve epistasis between different mutations, implying a rugged fitness landscape for isobutanol tolerance. We observe a common trend of evolution targeting post-transcriptional regulation and high centrality nodes of biochemical networks, and suggest that post-transcriptional regulators, such as *hfq*, RNA helicases, and sRNAs may be interesting mutagenesis targets for engineering complex stress tolerance phenotypes. Collectively, the genotypic adaptations we observe suggest mechanisms of adaptation to isobutanol stress based on remodeling the cell envelope and surprisingly, stress response attenuation.

## Materials and methods

### Base strains, media, and growth conditions

*E. coli *EcNR1 was used as the parent strain in our evolution studies. *E. coli *EcNR1 is a derivative of *E. coli *K12 MG1655 containing a modified λ prophage integrated at the *bioA*/*bioB *locus [37]. *E. coli *EcHW24 was used as a host strain for producing chromosomal mutations with ssDNA mediated recombination. *E. coli *EcHW24 is a MutS- derivative of *E. coli *EcNR1 containing 2864887 T→G and 2864892 G→T SNPs that produce premature stop codons in *mutS*. NG50 medium, consisting of M9 salts at 1× concentration, 50 g/L glucose, and 0.25 mg/L biotin, was used in the adaptive evolution of *E. coli *EcNR1 populations with glucose as a sole carbon source. NX50 medium, formulated similarly to NG50 with glucose replaced by 50 g/L xylose, was used in the adaptive evolution of *E. coli *EcNR1 populations on xylose. NG50 and NX50 agar media were prepared by supplementing NG50 and NX50 media with 15 g/L agar. LB Lennox broth (10 g/L tryptone, 5 g/L yeast extract, and 5 g/L NaCl) and LB agar (10 g/L tryptone, 5 g/L yeast extract, 10 g/L NaCl, and 15 g/L agar) were used for propagating strains during genetic manipulations and genomic DNA extraction. Clonal isolates from evolved populations were obtained by isolation streaking culture samples on LB agar plates or NG50/NX50 agar plates and propagating selected colonies. All *E. coli *strains used in this study were grown at 30°C with 150 to 200 rpm shaking.

### Adaptive evolution

Isobutanol tolerant lines of *E. coli *EcNR1 were evolved by serial passaging of three independent populations on isobutanol spiked NG50 medium (glucose as sole carbon source) and three independent populations on isobutanol spiked NX50 medium (xylose as sole carbon source). Cultures were grown in tightly capped 15 mL Falcon tubes containing 3 mL of respective medium. The initial isobutanol concentration was 0.75% (w/v) for all populations, and was gradually increased during the evolution to maintain an approximately constant selective pressure. Cultures were passaged when populations reached mid log phase, and the fresh cultures were inoculated to yield an initial optical density at 600 nm (OD_600_) = 0.002. Each lineage was periodically checked for contamination by isolation streaking culture samples on LB agar. Samples from each population were cryopreserved every 5 to 10 passages by centrifuging 2 × 1.4 mL samples from each culture at 14,000 rpm × 1 minute, washing the cell pellets with fresh medium, centrifuging again, and resuspending each cell pellet in 150 μL fresh medium + 150 μL cryopreservation solution (65% v/v glycerol and 0.1 M MgSO_4_). Cell suspensions were transferred to 96-well microplates, sealed with adhesive film, and stored at -80°C. The evolution proceeded for 180 days, corresponding to approximately 500 generations for the glucose lineages and 430 generations for the xylose lineages, assuming approximately 7.5 generations per passage.

### Phenotype evaluation

Isobutanol tolerance was quantified by measuring the maximum specific growth rate (μ_max_, h^-1^) and saturating OD_600 _at various isobutanol concentrations, using a microplate spectrophotometer. Inoculum was prepared by centrifuging 1 mL of overnight culture at 12,000 rpm × 2 minutes and resuspending cell pellets in a volume of fresh medium such that OD_600 _= 2. Standard 96-well microplates were filled with 200 μL medium per well (spiked with isobutanol or alcohols as appropriate) and seeded with 2 μL of prepared inoculum per well. Microplates were covered with adhesive film to prevent isobutanol evaporation and microplate lids were affixed with tape. OD_600 _was measured every 10 minutes for 48 hours using Molecular Devices Spectramax M5 or Molecular Devices Versamax plate readers, with 30°C incubation temperature and agitation between reads. μ_max _was calculated via linear regression of ln(OD_600_) vs. time (h) after subtracting blank values; regression was done over the time intervals corresponding to log growth phase.

### Genome resequencing

Genomic DNA (10 to 20 μg) was isolated from clonal isolates chosen for genome resequencing (G3.2, G3.6, G3.266.7, and X3.5) using a DNEasy spin column kit (Qiagen, Germantown, MD, USA) according to the manufacturer's protocol. Genomic DNA libraries were prepared using the Illumina genomic DNA library generation kit following the manufacturer's protocol (Illumina Inc., San Diego, CA, USA). Briefly, 5 to 10 μg genomic DNA was fragmented using a Covaris Acoustic System to ~ 200 bp DNA fragments. The ends of the fragmented DNA were then repaired with T4 DNA polymerase, Klenow DNA polymerase, and T4 PNK to convert the overhangs into phosphorylated blunt ends. Klenow fragment (3' to 5' exo minus) was used to add an 'A' base to the 3' end of the blunt phosphorylated DNA fragments. Following ligation of adapters to the ends of the DNA fragments, PCR was used to enrich the adapter-modified DNA fragments to obtain a DNA library suitable for high-throughput sequencing using Illumina Genome Analyzer. The concentration of DNA library was obtained by RT-PCR. Libraries for single-end sequencing were prepared for G3.2, G3.6, and X3.5 and a paired-end sequencing library was prepared for G3.266.7. Single-end libraries were sequenced with the Illumina Genome Analyzer 2 using a 36 cycle run, while the G3.266.7 paired-end library was sequenced on the same platform using 2 × 36 cycle runs.

### Sequence analysis

A custom perl script (available upon request) was written to automate the sequence analysis described below. Raw illumina reads were aligned to the *E. coli *EcNR1 reference sequence using Novoalign v2.04.02 [25]. Novoalign output was converted to MAQ (Mapping and Assembly with Qualities) map format, and MAQ v0.7.0 was used to build a consensus sequence and call SNPs and short indels [26]. Annotations and descriptions of mutated genes were downloaded from KEGG (Kyoto Encyclopedia of Genes and Genomes), and amino acid changes due to SNPs were automatically computed [75]. Large deletions were detected by tabulating coverage gaps in the alignments. To detect structural variation (SV) breakpoints with single-end reads, unmappable reads were filtered to eliminate long homopolymer runs (since erroneous homopolymer runs at tile edges are common with the Illumina platform) and *de novo *assembled with Velvet v0.7.51 [27]. For each set of reads, assemblies were done over a range of k-mer values. The assemblies were aligned to the *E. coli *EcNR1 reference sequence with BLAST, and were inspected manually to detect breakpoints [76]. For paired-end sequencing data, structural variations were detected using BreakDancer v0.0.1, using mean paired-end insert sizes calculated by MAQ [77]. Primer3 v2.0.0 was utilized to automatically design primers (with amplicon sizes of 800-1000 bp and melting temperatures of 60°C - 65°C) flanking detected mutations for Sanger sequencing verification [78]. For clones G3.2 and G3.6, only mutations shared between these two clones were verified by Sanger sequencing. In other sequenced clones, SNPs with consensus quality >200 (as computed by MAQ) or indels with frequency >0.4 were verified; we have found empirically that lower quality/frequency mutations are almost always false positives [26].

### Sanger sequencing

Mutations identified in the genome sequence analysis, as well as mutations reconstructed in *E. coli *EcHW24, were verified using Sanger sequencing. Sanger sequencing was also used to search for mutations in *marC*, *acrAB*, *tolC*, *mdh*, and *hfq *in evolved lineages that were not characterized by genome resequencing. See Additional file 7 for primer list. For mutation verification, 200 to 1000 bp regions containing putative mutations were amplified by PCR, using Phusion HotStart polymerase (Finnzymes USA, Woburn, MA) with manufacturer's recommended cycling conditions and a 6:00 minute initial denaturation at 95°C. Cell suspensions were prepared by resuspending colony material in 250 μL sterile water; 1 μL cell suspension was used as template per 50 μL PCR reaction. For mutation searches, the entire locus of interest plus at least 200 bp upstream/downstream was amplified, using Phusion HotStart polymerase for PCR. Agarose gel electrophoresis (50 mL 0.7% agarose gel, tris-acetate-EDTA (TAE) or tris-borate-EDTA (TBE) buffer, 98 V for 45 minutes running time) was used to verify PCR product size and reaction specificity. PCR products were purified with a QIAquick spin column kit (Qiagen), as per manufacturer's protocol. DNA concentration in purified PCR products was quantified with a Thermo Scientific Nanodrop spectrophotometer. Purified PCR products were diluted to 3 ng/μL/kb product size, and were Sanger sequenced with the same primers used for PCR. For amplicons >1200 bp in size, internal primers were designed and also used for sequencing, such that sequencing primers were spaced every 600 bp. Sanger sequencing was completed by the University of Michigan DNA Sequencing Core. Returned sequences were aligned to the reference *E. coli *EcNR1 sequence using BLAST, and chromatograms were manually inspected to verify mutations.

### Allele specific PCR

Primers were designed such that one primer in a pair was complementary to a mutation of interest at it's 3' end [79]. Under stringent PCR conditions, non-proofreading DNA polymerases are unable to extend from 3' mismatches, allowing genotype discrimination based on the presence or absence of a PCR product [79]. Allele specific primers for SNPs were designed using BatchPrimer3 v1.0, and allele specific primers for indels were designed manually [79]. See Additional file 7 for primer list. Platinum *Taq *polymerase (Invitrogen, Carlsbad, CA, USA) was used for PCR, with 0.2 μM of each primer and other reagent concentrations as per manufacturer's recommendations. Cell suspensions were used as template as described for Sanger sequencing above. Cycling conditions used were initial denaturation at 94°C for 6:00 minutes, followed by 28 cycles of 94°C denaturation for 0:30 minutes, optimal annealing temperature for 0:30 minutes, and 72°C elongation for 1:00 minute, followed by a final 72°C extension for 5:00 minutes. Optimal annealing temperature for each PCR reaction was determined via annealing temperature gradient with WT *E. coli *EcNR1 and appropriate mutants as controls. Agarose gel electrophoresis (50 mL 1% agarose gel, TBE buffer, 110 V for 30 minutes running time) was used to examine PCR products.

Multiplex allele specific PCR was done with a Qiagen Multiplex PCR kit, using manufacturer's recommended reagent concentrations and cell suspensions for template. Cycling conditions used were initial denaturation at 94°C for 15:00 minutes, followed by 28 cycles of 94°C denaturation for 0:30 minutes, optimal annealing temperature for 1:30 minutes, and 72°C elongation for 1:30 minutes, followed by a final 72°C extension for 10:00 minutes. Optimal annealing temperature for each PCR reaction was determined via annealing temperature gradient with *E. coli *EcNR1 and appropriate mutants as controls. Agarose gel electrophoresis (50 mL 3% agarose gel, TBE buffer, 110 V for 1 h 25 minutes running time) was used to examine PCR products.

### High efficiency ssDNA mediated homologous recombination

High efficiency ssDNA mediated mutagenesis in *E. coli *EcHW24 was used to engineer chromosomal SNPs and short indels, as per previously described procedures [37]. 90-mer oligonucleotides containing mutations of interest flanked by homologous genomic sequences were designed such that they targeted the lagging strand of the replication fork during DNA replication and had ΔG > -12.5 kcal/mol for secondary structures (evaluated with mfold v3.2) [80]. Oligos were synthesized by IDT (Integrated DNA Technologies, Coralville, IA) with four 5' phosphorothioated bases to enhance *in vivo *stability. See Additional file 7 for oligo list.

Homologous recombination was done by heat shocking *E. coli *EcHW24 to induce expression of λ-Red genes, preparing electrocompetent cells from the induced cultures, and electroporating the competent cells with oligonucleotide at 5 μM concentration. Overnight cultures of *E. coli *EcHW24 were inoculated 1:70 into fresh LB medium and incubated with shaking at 30°C until reaching OD_600 _= 0.7. Cultures were then heat shocked at 42°C for 15 minutes in a water bath with 200 rpm shaking. Immediately after heat shocking, the cultures were chilled on ice for 10 minutes. All subsequent manipulations were done at 4°C, which is vital for maximum recombination efficiency. For each electroporation, 1 mL of induced cells was centrifuged at 16,000 g × 1 minute. Cells were resuspended in 1 mL chilled ultrapure dH2O. The centrifugation and washing process was repeated twice. A final centrifugation was performed and the cell pellet was resuspended in 50 μL ultrapure dH2O. Oligonucleotide solution was mixed with the cell suspension such that the final DNA concentration was 5 μM. Immediately after adding oligonucleotide, cell/DNA mixture was transferred to a 0.1 cm gap electroporation cuvette and electroporated at 1.8 kV, using an Eppendorf Electroporator 2510. Cell mix was immediately resuspended in 1 mL room temperature LB medium, added directly to the electroporation cuvette. The resuspended cell mix was transferred to a Falcon tube, 1-5 mL LB medium was further added, and cells were allowed to recover at 30°C with shaking.

After 3-5 hours of incubation, 100 μL of 1:10^4 ^and 1:10^5 ^diluted recovery mix were plated on LB agar and incubated at 30°C overnight. The described recombination approach has efficiencies ranging from 1-40%, so selection is not required; mutants were recovered by direct genetic screens of colonies. For each recombination, 20 to 300 colonies were screened for desired mutations using Sanger sequencing or allele specific PCR (described above).

Multiplex recursive ssDNA mediated mutagenesis was used to generate a mutant set containing all combinations of *miaA-hfq*, *rph, mdh*, and *groL *mutations identified in the G3 lineage. The above homologous recombination procedure was carried out, using 5 μM oligonucleotide for each mutation (*miaA-hfq*, *rph, mdh*, and *groL*, 20 μM total) in electroporation mixes. *E. coli *EcHW24 was electroporated without oligonucleotides (oligo-) as a control. Recovery mixes were allowed to grow to OD_600 _= 0.7, and the homologous recombination procedure was repeated. This recursive homologous recombination process was repeated for a total of six cycles. 1:10^2^, 1:10^3^, and 1:10^4 ^dilutions of the final recovery mix were prepared and 100 μL aliquots were spread on NG50 plates supplemented with isobutanol at 0.7% (w/v). Plates were wrapped in parafilm to prevent isobutanol evaporation and incubated at 30°C for 48 hours. Clones showing improved isobutanol tolerance, judged by colony size and comparison with the oligo-control plate, were screened using allele specific PCR for *rph *and *groL *and Sanger sequencing for *miaA-hfq *and *mdh*. Putative *rph *and *groL *mutations were verified by Sanger sequencing.

### Construction of gene knockouts with P1 transduction

*E. coli *EcNR1 gene knockouts were constructed via P1*vir *transduction using Keio single gene knockout strains as donors, as described previously [81,82]. P1*vir *lysates were prepared for each Keio mutant (Δ*acrA::kan*, Δ*acrB::kan*, and Δ*mdh::kan*), and used to transduce gene knockouts to *E. coli *EcNR1 using LB agar with 100 μg/mL kanamycin as selective medium. Transductants were purified from residual P1*vir *phage by isolation streaking on LB agar supplemented with 0.8 mM sodium citrate and 100 μg/mL kanamycin, and verified by PCR as per published procedures [82].

### Construction of Δ*marC::kan *knockouts with homologous recombination

We were unable to generate Δ*marC::kan *knockouts using P1 transduction; dsDNA homologous recombination was used as an alternative procedure. A dsDNA cassette containing *kan *flanked by 50-100 bp of *marC *homologous sequence was produced via PCR (as per PCR procedure described for Sanger sequencing), using the Δ*marC::kan *Keio mutant as template (see Additional file 7 for primers). Homologous recombination was carried out using *E. coli *EcHW24 or progeny host strains. Procedures for heat shocking, preparation of electrocompetent cells, and electroporation were identical to those described for high efficiency ssDNA mediated recombination. 50 ng purified Δ*marC::kan *PCR product was used for each electroporation. Electroporation recovery mixes were incubated at 30°C for 2 hours, then centrifuged at 12,000 rpm × 1 minute. After discarding supernatant, cell pellets were spread on LB agar plates supplemented with 100 μg/mL kanamycin and incubated overnight at 30°C. Colonies were PCR screened to verify Δ*marC::kan *genotype.

### Microarray sample processing and data generation

#### Probe and microarray design

Microarray probes targeting coding sequences (CDSs) from the *Escherichia coli str. K12 substr. MG1655 *genome (Genbank NC_000913) were designed using the OligoArray software [83]. In a first pass using the following parameters (Probe length, 45-47 nucleotides; GC content 41-57% and Tm 83-91°C; Up to 3 probes per gene), we obtained 12037 probes targeting 4253 out of the 4292 CDSs annotated for this strain (99% of all CDSs). In a second pass, the GC content and Tm parameters were relaxed to 30-70% and 78-97°C, respectively. This led to the design of an additional 41 probes targeting 27 genes missed from the first pass for a total of 12078 probes targeting 4280 CDSs. Twelve genes (*thrL, ylbI, trpL, pheM, yojO, ypaB, ypdJ, pheL, yhaL, yrhD, yifL *and *pyrL*) failed the second pass and were excluded from this study. Each probe was replicated 6 times on the array, each replicate being randomly distributed across the whole array area for a total of 72,468 spots. Oligonucleotide probes were synthesized by MYcroarray (Ann Arbor, MI, USA) on an 80 K array format, one array per slide.

#### Sample preparation

Total RNA was extracted from mid log phase cultures using an RNeasy kit (Qiagen). 100 μL RNAase free water was used for final elution; a 90 μL aliquot was immediately ethanol precipitated and stored at -80°C. mRNA was enriched from 10 μg of total RNA using a MICROB*Express*™ Bacterial mRNA Purification Kit (#1905, Ambion, Austin, TX, USA) by the removal of 16S and 23S ribosomal RNAs. The enriched mRNA (200 ng) was converted to cRNA containing aminoallyl-UTP with a Message Amp II - Bacteria Prokaryotic RNA amplification kit (#1790, Ambion) following the manufacturer's instructions. Amino-allyl modified cRNA (45 μg) was coupled with amine reactive fluorescent dye (Alexa Fluor-555, #32756, Invitrogen) in a 10 μL reaction following the manufacturer's instructions. After fluorescent dye coupling, unincorporated dye was removed with RNEasy mini columns (Qiagen) following the manufacturer's instructions. Labeled cRNA was eluted from RNeasy mini columns with RNAse/DNAse free water. The extent of dye incorporation was determined by the Microarray function on a NanoDrop 1000 spectrophotometer (Thermo Scientific). The dye incorporation calculations were performed as described by Invitrogen/Molecular Probes for Alexa Fluor dye products. All samples had dye incorporation between 35 to 43 bases per dye. Fluorescent dye coupled cRNA (20 μg) was fragmented by exposure to zinc sulphate (5 mM final concentration in a 60 μL reaction) at 75°C for 10 minutes and the reaction was stopped by the addition of 500 mM EDTA to a final concentration of 20 mM. The extent of fragmentation was visualized with a 2100 Bioanalyzer (Agilent Technologies, Santa Clara, CA, USA) using a RNA Nano Chip (Agilent Technologies, #5067-1511). Samples with a mean fragment size of 100-200 nucleotides were qualified for hybridization.

#### Hybridization

Each labeled and fragmented cRNA sample was hybridized individually to one custom 80 K microarray by dynamic hybridization as follows: 20 μg of sample was added to hybridization solution (600 μL final volume) and incubated at 65°C for 5 minutes and then placed on ice. Hybridization solution contained 6X SSPE (1M NaCl, 6.7 mM EDTA, 40 mM NaH_2_PO_4 _and 27.3 mM Na_2_HPO_4_), 0.01 μg/μl acetylated BSA (#R3961, Promega, Madison, WI, USA), 0.01% Tween-20 (#P9416, Sigma, Saint Louis, MO, USA), and 10% deionized formamide (#P9037, Sigma). A large volume of hybridization solution master mix was prepared from which aliquots were removed to prepare each sample. Hybridizations were performed using a hybridization gasket slide (#G2534-60003, Agilent Technologies) and a Microarray Hybridization Chamber assembly (#G2534A, Agilent Technologies) following the manufacturer's instructions except that 585 μL of hybridization solution was used per gasket slide and both the gasket slide and microarray slide were preheated to 65°C prior to Hybridization Chamber assembly. The final Hybridization Chamber assembly was incubated at 50°C for 20 hours while rotating at ~5 rpm (to assure free movement of the mixing bubble) in a hybridization oven (#G2545A, Agilent Technologies).

#### Washing and scanning

Following hybridization, unbound material was removed as follows: The hybridization chamber assembly was quickly removed from the hybridization oven and the microarray/gasket slide sandwich was immediately submerged in 1× SSPE at room temperature. The slide was quickly transferred to fresh wash solution (1× SSPE) at room temperature and incubated for 3 minutes with gentle agitation. This wash was repeated twice, however, the first repeat was performed at 50°C. Finally, the slide was rinsed for 30 seconds in 0.25× SSPE at room temperature and immediately spun dry in a Microarray Minifuge (ArrayIt, Sunnyvale, CA, USA). Slides were immediately scanned using an Axon 4000B scanner (Molecular Devices) at 5 micron resolution and 100% laser power. The PMT gain in the 532 nm channel was adjusted to appreciate the full dynamic range (0-65,000) of signal intensity such that only a few pixels were saturated in a few spots.

#### Data Extraction

A signal intensity value for each probe on the array was extracted from the scanned image using Axon GenePixPro 6.1 software (version 6.1.0.4, Molecular Devices). Fixed diameter (35 microns) circular feature indicators were placed over the centre of each spot (probe) and median pixel intensity was calculated for each feature.

### Microarray data analysis

#### Pre-processing

First, background fluorescence intensities of individual spots were subtracted directly from foreground intensities for background adjustment. Then variance-stabilizing normalization (vsn) and quantile methods without weight were applied sequentially for normalization with software R (2.11.0, "vsn" and "aroma.light" packages [84-88]). For each strain/isobutanol condition, spots with acceptable signals in at least two out of three biological replicates were chosen for further analysis, which resulted in 4-6 replicates for each probe. Two or three probes for each gene were included on the array. For simplicity, we chose the probe with the smallest overall variation across replicates (measured by the sum of standard deviations across 12-18 technical/biological replicates over all the four strain/isobutanol conditions) to represent the gene in further analysis. For each chosen probe, the median of the technical replicates on each array was calculated to represent the expression level of the corresponding gene for each sample. The above pre-processing procedure provided normalized expression data for 4235 genes (out of 4280 included on the array). The complete dataset has been deposited in the GEO database (http://www.ncbi.nlm.nih.gov/geo) under accession number GSE23526.

#### Identification of differentially expressed/regulated genes

We first applied two filters to select genes that showed notable expression changes, namely by choosing genes with expression levels higher than 100 units in at least 25% of the 12 samples (i.e. 3 samples) and interquartile ranges (IQR) larger than 0.5 [84,89]. This led to a set of 2026 genes for further analysis and two-sample student's t-test was carried out to identify genes that showed differential expression. In particular, three t-tests were conducted: a t-test between WT/0% isobutanol and WT/0.5% isobutanol; a t-test between G3.2/0% isobutanol and G3.2/0.5% isobutanol; and a t-test between changes in G3.2 upon isobutanol treatment and changes in WT upon isobutanol treatment. The first two t-tests identified differentially expressed genes in each strain and the last t-test identified genes that responded to isobutanol differently between G3.2 and WT. Empirical Bayes statistics were applied to remove chip effects with software R (2.11.0, "limma" package [90], parameters: assumed proportion of differentially expressed genes equal to 0.01, assumed lower and upper limits for the standard deviation of log2 fold changes for differentially expressed genes equal to 0.1 and 4 respectively [91,92]). In addition, the p-values of the t-test were adjusted by controlling false discovery rate (FDR, BH procedure) [93]. Genes with adjusted p-values less than 0.001 were considered as differentially expressed in the first two t-tests or responding to isobutanol differently in the last t-test.

#### Regulatory network analysis

Network Component Analysis (NCA) was carried out with NCA toolbox (v2.3 for Matlab, released Feb. 19, 2007) [94,95] to predict transcription factor activities using microarray data. Based on previous study of the isobutanol response network in *E. coli *[7] and preliminary examination of our microarray data, we selected 16 transcription factors that are potentially involved in isobutanol tolerance (ArcA, PdhR, Fnr, Fur, FlhDC, OmpR, CRP, GadE, MarA, Nac, LexA, PurR, Fis, IHF, PhoB and PhoP) for this analysis. The corresponding transcriptional network was constructed based on connectivity data in RegulonDB (v6.7, MAR.2, 2010) [96]. A total of 998 genes from the above pre-processed expression dataset were regulated by this set of TFs, with each gene regulated by 1 to 5 TFs (1.5 on average). Due to limited data (i.e. four strain/isobutanol conditions), we used a subset of four TFs in each NCA analysis and repeated the analysis for different combinations of the TFs. Only TFs that were consistently predicted to change significantly across different combinations of TFs and different replicates were kept for further rounds of analysis; at the end, four TFs that showed the most significant activity changes were obtained (FlhDC, GadE, MarA and PhoP). In the study described in [7], it was concluded that activities of ArcA, PhoB, and Fur were significantly perturbed by isobutanol due to quinone/quinol malfunction. We performed NCA for various combinations of ArcA, PhoB, and Fur with FlhDC, GadE, MarA, and PhoP (using up to six TFs in each NCA) to determine whether these results were recapitulated in our study. For each strain/condition, TF activities were calculated for all the biological replicates and we report their average value and 95% confidence interval in Figure 6D.

### qRT-PCR validation of gene expression changes

Total RNA extraction and mRNA enrichment were carried out as described for microarray sample processing, with three biological replicates per strain/condition. Biological replicates were pooled and 100 ng enriched mRNA was reverse transcribed using a QuantiTect Reverse Transcription kit (Qiagen) as per manufacturer's protocol. Quantitative PCR assays were performed in 25 μL samples on an MJ Research (BioRad, Hercules, CA, USA) Chromo4 thermal cycler with a QuantiTect SYBR Green RT-PCR kit (Qiagen), using primer pairs designed as per manufacturer's recommendations for genes *gadA, fimI, fabA, rfaJ*, and *rpoD *(Additional file 7). 5 ng reverse transcribed cDNA was used as template for each reaction, with primer and other reagent concentrations as per manufacturer's protocol. Cycling conditions used were initial denaturation at 95°C for 15:00 minutes, followed by 45 cycles of 94°C denaturation for 0:15 minutes, 60°C annealing for 0:30 minutes, 72°C elongation for 0:30 minutes, and fluorescence measurement at 72°C. After the final cycle, reaction specificity was verified by determining melting profiles over a temperature range of 65°C to 95°C in 0.2°C increments. All assays were run in triplicate. qRT-PCR data was analyzed by fitting parameters of the MAK2 model (initial target concentration D_0 _and characteristic PCR constant *k*), using a custom Mathematica script [31]. Initial target concentrations were normalized to *rpoD.*

### Fatty acid analysis

Duplicate 100 mL cultures of *E. coli *EcNR1 and 50 mL cultures of G3.2 were grown to mid log phase in NG50 medium supplemented with 0.5% (w/v) isobutanol. Cultures were harvested by centrifugation at 3,200 g × 10 minutes. Cell pellets were washed with Phosphate Buffered Saline (PBS) and dried at 70°C for 24 hours. The dry cell pellets were subjected to ethanolysis at 90°C for 2 hours in 5% HCl in anhydrous ethanol. The resulting Fatty Acid Ethyl Esters (FAEEs) were quantified with an Agilent 6890 GC equipped with a 50 m × 0.2 mm × 0.33 mm HPx5 capillary column, flame ionization detector (FID), and autoinjector. 2 μL FAEE samples were analyzed after split injection (1:10). Helium was used as a carrier gas with a constant flow rate of 1.9 mL/min. The temperatures of the injector and detector were 325 and 350°C, respectively. The following temperature program was applied: 50°C for 3 minutes, increase 10°C/min to 300°C, then 300°C for 10 minutes.

### Cell envelope protein analysis

50 mL cultures of *E. coli *EcNR1 and G3.2 were grown to mid log phase in NG50 medium supplemented with 0.5% (w/v) isobutanol. 5 × 10^9 ^cells (estimated by OD_600_) were harvested by centrifugation at 5,000 g × 10 minutes. Cell pellets were washed with PBS and resuspended in 3 mL 10 mM NaH_2_PO_4_-NaOH (pH 7.2) buffer. Cell suspensions were placed on ice and lysed by sonication (15 W continuous output; 30 seconds sonication followed by 30 seconds cooling; repeated six times total). Unbroken cells were removed by centrifugation at 1,500 g × 20 minutes. Supernatant was ultracentrifuged at 100,000 g × 1 hour at 4°C to pellet cell envelopes. Cell envelope pellets were washed with 10 mM NaH_2_PO_4_-NaOH (pH 7.2), resuspended in 20 μL dH_2_O plus 20 μL 2× Laemmli buffer (BioRad), and then incubated at 95°C for 5 minutes. Cell envelope samples were then analyzed with SDS-PAGE (12.5% gel; 25 μL loading volume; 200 V for 55 minutes). After electrophoresis, gel was incubated in Coomassie staining solution (50% v/v methanol, 10% v/v acetic acid, and 0.25% w/v Coomassie brilliant blue R-250 in dH_2_O) for 40 minutes with gentle shaking and then destained by boiling in 1 L dH_2_O for 20 minutes. Protein band intensity was quantified by densitometry analysis (using ImageJ software) and normalized to the sum of intensities of the major protein bands (ie the 95 kDa, 72 kDa, 55 kDa, 44 kDa, OmpC/OmpF, OmpA, 29 kDa, and 26 kDa bands). Experiment was repeated to verify results.

### RpoS Western blot

Total cellular protein was extracted from early log phase cultures using B-PER lysis buffer (Thermo Fisher Scientific, Waltham, MA, USA) as per manufacturer's protocol. Protein concentration was quantified using Bradford assay [97]. Samples were diluted with PBS to 4 μg/μL protein and mixed 1:1 with 2× Laemmli buffer (BioRad) and then incubated at 95°C for 5 minutes. Protein samples were separated with SDS-PAGE (12.5% gel; 10 μL loading volume, corresponding to 20 μg protein; 100 V for 55 minutes) and then electroblotted onto a 0.22 μm nitrocellulose membrane (BioRad). Membrane was blocked by incubating for one hour with gentle shaking in 15 mL Tris-Buffered Saline Tween-20 (TBST) buffer (BioRad) supplemented with 5% (w/v) nonfat dry milk (BioRad). After blocking, membrane was incubated for 2 hours with gentle shaking in 15 mL blocking buffer (TBST with 5% w/v milk) supplemented 1:1000 with primary mouse anti-RpoS antibody (NeoClone, Madison, WI, USA). Membrane was then washed 3 times with TBST and incubated for one hour with gentle shaking in blocking buffer supplemented 1:2000 with secondary goat anti-mouse IgG horseradish peroxidase conjugated antibody (Jackson ImmunoResearch, West Grove, PA, USA). After incubation with secondary antibody, membrane was washed 3 times with TBST and treated with Immun-Star horseradish peroxidase substrate (BioRad) for detection, as per manufacturer's protocol. Experiment was repeated to verify results. Purified *E. coli *RpoS (NeoClone) was used as a positive control.

### Ethidium bromide accumulation assay

Ethidium bromide accumulation assays were carried out as described previously, with minor modifications [38]. For each strain tested, LB medium was inoculated 1:70 with saturated overnight culture and incubated at 30°C with shaking. When cultures reached OD_600 _= 0.6, 600 μL aliquots were withdrawn and centrifuged at 13,000 rpm × 3 minutes. Supernatant was discarded and cell pellets were resuspended in 1000 μL sterile phosphate buffer saline (PBS). The centrifugation and washing process was repeated once more. After a final centrifugation, cell pellets were resuspended in PBS such that OD_600 _= 0.3 in the final cell suspension. Costar black/clear bottom 96-well microplates (Thermo Fisher Scientific) were filled with 2 μL 0.1 mg/mL ethidium bromide per well and 198 μL OD_600 _= 0.3 cell suspension for 200 μL total volume. Fluorescence (518 nm excitation/605 nm emission) was measured every minute for 60 minutes using a Molecular Devices Spectramax M5 plate reader, with 25°C incubation temperature and agitation between reads. Specific fluorescence was calculated by dividing measure fluorescence by OD_600_.

### Malate dehydrogenase assay

Malate dehydrogenase assays were carried out as described previously, with modifications [98]. For each strain tested, LB medium was inoculated 1:70 with saturated overnight culture and incubated at 30°C with shaking. When cultures reached OD_600 _= 0.6, 5 mL aliquots were withdrawn from each culture and centrifuged at 5000 g × 10 minutes. Supernatant was discarded. Cell pellets were suspended in 50 μL B-PER (Thermo Fisher Scientific) lysis buffer and incubated for 15 minutes at room temperature. After incubation, cell lysates were centrifuged at 15,000 g × 5 minutes at 4°C. Lysis supernatant was reserved and kept on ice for the remainder of the procedure. Standard 96-well microplates were filled with 185.6 μL PBS, 4.45 μL 10 mM NADH, and 10 μL 1:10 diluted lysate supernatant per well. Absorbance at 340 nm was measured every 30 seconds for 10 minutes at 30°C with shaking to determined background NADH oxidation rate. 22.2 μL 20 mM oxaloacetate was then added to each well. Absorbance at 340 nm was measured every 30 seconds for 15 minutes at 30°C with shaking to determine rate of NADH oxidation. Malate dehydrogenase activity was calculated as Units/g-wet-cells =  where Δ*A*_340_/min = rate of change in absorbance at 340 nm wavelength (AU/min), ε_340 _= NADH molar extinction coefficient at 340 nm wavelength (6.22 mM^-1^cm^-1^), *l *= 1 cm, and *c *= 0.0045 g-wet cells per reaction volume.

### Iodine staining assay

Iodine staining assay was adapted from a previously described procedure [40]. Overnight cultures of selected strains were streaked on NG50 agar spiked with 0.35% (w/v) isobutanol. The plate was tightly wrapped in parafilm, incubated at 30°C for 48 hours, and then flooded with 5 mL USP tincture of iodine. After 2 minutes of incubation at room temperature, excess iodine was poured off the plate, and iodine was allowed to evaporate until media was translucent.

## Abbreviations

NCA: Network Component Analysis; G1: glucose lineage #1; G2: glucose lineage #2; G3: glucose lineage #3; X1: xylose lineage #1; X2: xylose lineage #2; X3: xylose lineage #3; WT: *E. coli *EcNR1 parent strain; G3.2: clonal isolate #2 from G3 population; X3.5: clonal isolate #5 from X3 population; G3.266.7: clonal isolate #7 from generation 266 of the G3 lineage; MIC: minimum growth inhibiting concentration; MB: million base pairs; SNP: single nucleotide polymorphisms; indel: insertion/deletion mutation; SV: structural variation; MMR: methyl-directed mismatch repair system; BiNGO: Biological Network Gene Ontology tool; GO: gene ontology; qRT-PCR: real time quantitative reverse transcription polymerase chain reaction; LPS: lipopolysaccharide; NCA: Network Component Analysis; TF: transcription factor; ssDNA: single stranded DNA; CDSs: coding sequences; GC-FID: gas chromatography-flame ionization detector; SDS-PAGE: sodium dodecyl sulfate polyacrylamide gel electrophoresis; EtBr: ethidium bromide; QSS: quorum sensing signal; sRNA: small RNA; GlcN-6-P: glucosamine-6-phosphate; pEtN: phosphoethanolamine; KDO: 3-deoxy-d-*manno*-octulosonic acid; ACP: acyl carrier protein; ppGpp: guanosine tetraphosphate; OD_600_: optical density at 600 nm; KEGG: Kyoto Encyclopedia of Genes and Genomes; BLAST: Basic Local Alignment Search Tool; TAE: tris acetate EDTA; TBE: tris borate EDTA; IQR: interquartile range; FDR: false discovery rate; PBS: phosphate buffered saline; FAEE: Fatty Acid Ethyl Ester; TBST: Tris-Buffered Saline Tween-20; RBS: ribosome binding site.

## Competing interests

EG and JMR are the founders of MYcroarray, the company which manufactured the custom DNA microarrays used in this study. All other authors declare that they have no competing interests.

## Authors' contributions

This study was designed by JJM and XNL. JJM and AAL performed adaptive evolution; JJM, AAL, and FL performed phenotyping experiments. Genome resequencing was carried out by BX, CAM, and YG (G3.2, X3.5, and G3.266.7) and the University of Michigan DNA sequencing core (G3.6). JJM analyzed the genome sequencing data. JJM, AAL, and FL performed mutation verification and parallel evolution investigation experiments. JJM genotyped intermediate generations of the evolved lineages. JJM, JMR, EG, and XNL planned the gene expression study. JMR designed the microarray probes; JJM, RJW, and DRS performed the microarray experimental work. JJM performed the qRT-PCR validation of microarray data. ABV analyzed a preliminary set of microarray data, while JJM and YC analyzed the full data set. JJM, AAL, and TAZ performed single/multiple mutation reconstruction, functional effect studies, and the iodine staining assay; JJM and FL carried out gene knockout experiments. JJM performed RpoS Western blots and cell envelope protein profiling; JJM and FL performed fatty acid composition analysis. The manuscript was written by JJM, with assistance from YC, DRS, and JMR, and final revision by XNL. All authors read and approved the final manuscript.

## Supplementary Material

Additional file 1 **Full mutation list**. Full lists of SNP, indel, and SV mutations discovered in G3.2, G3.6, G3.266.7, and X3.5 with Illumina sequencing. Mutation positions are genomic coordinates in the *E. coli *EcNR1 reference sequence, gene descriptions are from the KEGG database, mutation frequency is defined as mutant reads divided by the total number of mapped reads at a position, and consensus quality was computed by MAQ [26]. For G3.2, G3.266.7, and X3.5, SNPs with consensus quality <150 or indels with frequency <0.4 were discarded; we have found empirically that lower quality/frequency mutations tend to be false positives. G3.6 may have been contaminated with another clonal isolate and thus may be mixed genotype; to reduce false negatives, quality cutoff thresholds were lowered to consensus quality <100 for SNPs and frequency <0.15 for indels. Mutations that were later discovered to be heterogeneities in the parent *E. coli *EcNR1 strain were discarded. Entries with red text were verified by Sanger sequencing. Click here for file

Additional file 2 ***E. coli *EcNR1 genome reference sequence**. *E. coli *EcNR1 is a derivative of *E. coli *K12 MG1655 containing a modified λ prophage integrated at the *bioA*/*bioB *locus. We created a reference genome sequence for *E. coli *EcNR1 by adding the above genetic modification to the *E. coli *K12 MG1655 reference sequence (NC_000913) obtained from the National Center for Biotechnology Information Reference Sequence Collection (NCBI RefSeq). We provide three formats: Lasergene DNA (.seq), FASTA (.fas), and GenBank (.gbk). Click here for file

Additional file 3 **Tracing mutations found in endpoint populations through intermediate generations**. We investigated the dynamics of genotypic adaptation in the G3 and X3 lineages by genotyping population samples from intermediate generations for selected mutations identified in the end point populations. Genotyping was conducted by screening whole-population cryopreserved samples for mutations with Sanger sequencing of PCR amplified mutated regions (for G3 *mdh *and *miaA-hfq *mutations), inferred from PCR product size for large insertion mutations (*marC *transposon insertions, X3 *mdtj::IS5::tqsA*, and G3 *glnE::IS186*) or using allele specific PCR (all other genotyped mutations). WT designates wild-type allele (directly detected in Sanger sequencing or inferred from lack of allele specific PCR product), Mut designates mutant allele (directly detected in Sanger sequencing or inferred from of amplification of allele specific PCR product), NT designates not tested. Strength (strong, weak, etc) indicates band intensity on agarose gel electrophoresis of PCR product, and is roughly correlated with allele frequency (allele specific PCR and large insertions). Sanger sequencing and genotyping via PCR product sizes allow discrimination of mixed genotypes, which are reported where applicable. Click here for file

Additional file 4 **Microarray Data and Analyses**. Microarray data for gene expression study of G3.2 and the parent *E. coli *EcNR1 (WT) in 0% and 0.5% (w/v) isobutanol glucose minimal medium. Genes that responded to isobutanol most differently between G3.2 and WT are tabulated with p-values and transcription factors controlling them. Differentially expressed genes, p-values, and related transcription factors are also tabulated for WT/0% isobutanol and WT/0.5% isobutanol, G3.2/0% isobutanol and G3.2/0.5% isobutanol, and lastly WT/0.5% isobutanol and G3.2/0.5% isobutanol. BiNGO was used to assess any overrepresented GO terms amongst genes that responded to isobutanol most differently between G3.2 and WT, and for genes differentially expressed between WT/0.5% isobutanol and G3.2/0.5% isobutanol. A summary of NCA results is given as well. Click here for file

Additional file 5 **qRT-PCR validation of *gadA, fimI, fabA*, and *rfaJ *gene expression changes**. qRT-PCR was used to validate gene expression changes measured by DNA microarray. Target concentrations were determined by fitting the MAK2 PCR model to qRT-PCR data [31]. Expression levels were normalized to house keeping gene *rpoD *(sigma factor 70). **(A) ***rpoD *normalized expression levels determined by qRT-PCR **(B) **Expression levels from DNA microarray study. Click here for file

Additional file 6 ***acrAB *and *mdh *functional assays**. AcrAB-TolC efflux pump activity was measured via ethidium bromide (EtBr) accumulation in reconstructed single mutants and clonal isolates harbouring *acrAB *mutations from evolution end populations. Mid log phase cells were incubated with ethidium bromide and intracellular ethidium bromide was monitored via relative fluorescence (518 nm excitation/605 nm emission). Mdh (NADH dependent malate dehydrogenase) activity was assayed by incubating cell extracts with oxaloacetate and NADH; disappearance of NADH (due to reduction of oxaloacetate to malate) was monitored by measuring absorbance at 340 nm. **(A) **EtBr accumulation assay for the parent *E. coli *EcNR1 (WT), clonal isolates from evolution end populations harbouring *acrA *mutations (G1.1, X2.1, X3.5), a reconstructed *acrA *single mutant (containing mutation found in X3.5), and Δ*acrA::kan *control. **(B) **EtBr accumulation assay for the parent *E. coli *EcNR1 (WT), clonal isolates from evolution end populations harbouring *acrB *mutations (X1.1, G3.2), a reconstructed *acrB *single mutant (containing mutation found in G3.2), and Δ*acrB::kan *control. **(C) **Mdh assay for the parent *E. coli *EcNR1 (WT), clonal isolates from evolution end populations harbouring *mdh *mutations (G3.2, X3.5), reconstructed *mdh *single mutants (containing mutations found in G3.2 or X3.5), and Δ*mdh::kan *control. Click here for file

Additional file 7 **Primers and oligos used in this study**. Sequences of forward and reverse primers used for Sanger sequencing, allele specific PCR, qRT-PCR, and all other PCR reactions described in this study are listed. Sequences of oligonucleotides used for ssDNA mediated homologous recombination are also given. Click here for file

## References

[B1] Stephanopoulos G Challenges in engineering microbes for biofuels production Science 2007 315 801 804 10.1126/science.1139612 17289987

[B2] Atsumi S Higashide W Liao JC Direct photosynthetic recycling of carbon dioxide to isobutyraldehyde Nat Biotechnol 2009 27 1177 1180 10.1038/nbt.1586 19915552

[B3] Atsumi S Hanai T Liao JC Non-fermentative pathways for synthesis of branched-chain higher alcohols as biofuels Nature 2008 451 86 89 10.1038/nature06450 18172501

[B4] Connor MR Liao JC Microbial production of advanced transportation fuels in non-natural hosts Curr Opin Biotechnol 2009 20 307 315 10.1016/j.copbio.2009.04.002 19473829

[B5] Zhang Y Zhu Y Zhu Y Li Y The importance of engineering physiological functionality into microbes Trends in Biotechnology 2009 27 664 672 10.1016/j.tibtech.2009.08.006 19793618

[B6] Nicolaou SA Gaida SM Papoutsakis ET A comparative view of metabolite and substrate stress and tolerance in microbial bioprocessing: From biofuels and chemicals, to biocatalysis and bioremediation Metab Eng 2010 12 4 307 31 10.1016/j.ymben.2010.03.004 20346409

[B7] Brynildsen MP Liao JC An integrated network approach identifies the isobutanol response network of Escherichia coli Mol Syst Biol 2009 5 277 10.1038/msb.2009.34 19536200 PMC2710865

[B8] Rutherford BJ Dahl RH Price RE Szmidt HL Benke PI Mukhopadhyay A Keasling JD Functional genomic study of exogenous n-butanol stress in Escherichia coli Appl Environ Microbiol 2010 76 1935 1945 10.1128/AEM.02323-09 20118358 PMC2838030

[B9] Warner JR Patnaik R Gill RT Genomics enabled approaches in strain engineering Current Opinion in Microbiology 2009 12 223 230 10.1016/j.mib.2009.04.005 19467921

[B10] Goodarzi H Bennett BD Amini S Reaves ML Hottes AK Rabinowitz JD Tavazoie S Regulatory and metabolic rewiring during laboratory evolution of ethanol tolerance in E. coli Mol Syst Biol 2010 6 378 10.1038/msb.2010.33 20531407 PMC2913397

[B11] Larossa RA Smulski DR IMPROVED STRAIN FOR BUTANOL PRODUCTION 2009 United States: BUTAMAX™ ADVANCED BIOFUELS LLC (Organization WIP ed., vol. (WO/2009/086075)

[B12] Borden JR Papoutsakis ET Dynamics of genomic-library enrichment and identification of solvent tolerance genes for Clostridium acetobutylicum Appl Environ Microbiol 2007 73 3061 3068 10.1128/AEM.02296-06 17337545 PMC1892849

[B13] Klein-Marcuschamer D Santos CN Yu H Stephanopoulos G Mutagenesis of the bacterial RNA polymerase alpha subunit for improvement of complex phenotypes Appl Environ Microbiol 2009 75 2705 2711 10.1128/AEM.01888-08 19251886 PMC2681691

[B14] Alper H Stephanopoulos G Global transcription machinery engineering: a new approach for improving cellular phenotype Metab Eng 2007 9 258 267 10.1016/j.ymben.2006.12.002 17292651

[B15] Shendure J Ji H Next-generation DNA sequencing Nat Biotechnol 2008 26 1135 1145 10.1038/nbt1486 18846087

[B16] Herring CD Raghunathan A Honisch C Patel T Applebee MK Joyce AR Albert TJ Blattner FR van den Boom D Cantor CR Palsson BO Comparative genome sequencing of Escherichia coli allows observation of bacterial evolution on a laboratory timescale Nat Genet 2006 38 1406 1412 10.1038/ng1906 17086184

[B17] Conrad TM Joyce AR Applebee MK Barrett CL Xie B Gao Y Palsson BO Whole-genome resequencing of Escherichia coli K-12 MG1655 undergoing short-term laboratory evolution in lactate minimal media reveals flexible selection of adaptive mutations Genome Biol 2009 10 R118 10.1186/gb-2009-10-10-r118 19849850 PMC2784333

[B18] Lee DH Palsson BO Adaptive evolution of Escherichia coli K-12 MG1655 during growth on a Nonnative carbon source, L-1,2-propanediol Appl Environ Microbiol 2010 76 4158 4168 10.1128/AEM.00373-10 20435762 PMC2897412

[B19] Harris DR Pollock SV Wood EA Goiffon RJ Klingele AJ Cabot EL Schackwitz W Martin J Eggington J Durfee TJ Directed evolution of ionizing radiation resistance in Escherichia coli J Bacteriol 2009 191 5240 5252 10.1128/JB.00502-09 19502398 PMC2725583

[B20] Barrick JE Yu DS Yoon SH Jeong H Oh TK Schneider D Lenski RE Kim JF Genome evolution and adaptation in a long-term experiment with Escherichia coli Nature 2009 461 1243 1247 10.1038/nature08480 19838166

[B21] Atsumi S Wu TY Machado IMP Huang WC Chen PY Pellegrini M Liao JC Evolution, genomic analysis, and reconstruction of isobutanol tolerance in Escherichia coli Mol Syst Biol 2010 6 449 10.1038/msb.2010.98 21179021 PMC3018172

[B22] Elena SF Lenski RE Evolution experiments with microorganisms: the dynamics and genetic bases of adaptation Nat Rev Genet 2003 4 457 469 10.1038/nrg1088 12776215

[B23] Kim Y Ingram LO Shanmugam KT Construction of an Escherichia coli K-12 mutant for homoethanologenic fermentation of glucose or xylose without foreign genes Appl Environ Microbiol 2007 73 1766 1771 10.1128/AEM.02456-06 17259366 PMC1828829

[B24] Griepernau B Leis S Schneider MF Sikor M Steppich D Bockmann RA 1-Alkanols and membranes: a story of attraction Biochim Biophys Acta 2007 1768 2899 2913 10.1016/j.bbamem.2007.08.002 17916322

[B25] Technologies N Novoalign 2009 Kuala Lumpur 2.04.02 edition.

[B26] Li H Ruan J Durbin R Mapping short DNA sequencing reads and calling variants using mapping quality scores Genome Res 2008 18 1851 1858 10.1101/gr.078212.108 18714091 PMC2577856

[B27] Zerbino DR Birney E Velvet: algorithms for de novo short read assembly using de Bruijn graphs Genome Res 2008 18 821 829 10.1101/gr.074492.107 18349386 PMC2336801

[B28] Maere S Heymans K Kuiper M BiNGO: a Cytoscape plugin to assess overrepresentation of gene ontology categories in biological networks Bioinformatics 2005 21 3448 3449 10.1093/bioinformatics/bti551 15972284

[B29] Krishnamoorthy G Tikhonova EB Zgurskaya HI Fitting periplasmic membrane fusion proteins to inner membrane transporters: mutations that enable Escherichia coli AcrA to function with Pseudomonas aeruginosa MexB J Bacteriol 2008 190 691 698 10.1128/JB.01276-07 18024521 PMC2223704

[B30] Link TM Valentin-Hansen P Brennan RG Structure of Escherichia coli Hfq bound to polyriboadenylate RNA Proc Natl Acad Sci USA 2009 106 19292 19297 10.1073/pnas.0908744106 19889981 PMC2773200

[B31] Boggy GJ Woolf PJ A mechanistic model of PCR for accurate quantification of quantitative PCR data PLoS One 2010 5 e12355 10.1371/journal.pone.0012355 20814578 PMC2930010

[B32] Savli H Karadenizli A Kolayli F Gundes S Ozbek U Vahaboglu H Expression stability of six housekeeping genes: A proposal for resistance gene quantification studies of Pseudomonas aeruginosa by real-time quantitative RT-PCR J Med Microbiol 2003 52 403 408 10.1099/jmm.0.05132-0 12721316

[B33] Guisbert E Rhodius VA Ahuja N Witkin E Gross CA Hfq modulates the sigmaE-mediated envelope stress response and the sigma32-mediated cytoplasmic stress response in Escherichia coli J Bacteriol 2007 189 1963 1973 10.1128/JB.01243-06 17158661 PMC1855744

[B34] Dong T Schellhorn HE Control of RpoS in global gene expression of Escherichia coli in minimal media Mol Genet Genomics 2009 281 19 33 10.1007/s00438-008-0389-3 18843507

[B35] Miyashiro T Goulian M Stimulus-dependent differential regulation in the Escherichia coli PhoQ PhoP system Proc Natl Acad Sci USA 2007 104 16305 16310 10.1073/pnas.0700025104 17909183 PMC2042202

[B36] Moon K Gottesman S A PhoQ/P-regulated small RNA regulates sensitivity of Escherichia coli to antimicrobial peptides Mol Microbiol 2009 74 1314 1330 10.1111/j.1365-2958.2009.06944.x 19889087 PMC2841474

[B37] Wang HH Isaacs FJ Carr PA Sun ZZ Xu G Forest CR Church GM Programming cells by multiplex genome engineering and accelerated evolution Nature 2009 460 894 898 10.1038/nature08187 19633652 PMC4590770

[B38] Viveiros M Martins A Paixao L Rodrigues L Martins M Couto I Fahnrich E Kern WV Amaral L Demonstration of intrinsic efflux activity of Escherichia coli K-12 AG100 by an automated ethidium bromide method Int J Antimicrob Agents 2008 31 458 462 10.1016/j.ijantimicag.2007.12.015 18343640

[B39] Yang S Lopez CR Zechiedrich EL Quorum sensing and multidrug transporters in Escherichia coli Proc Natl Acad Sci USA 2006 103 2386 2391 10.1073/pnas.0502890102 16467145 PMC1413681

[B40] Notley-McRobb L King T Ferenci T rpoS mutations and loss of general stress resistance in Escherichia coli populations as a consequence of conflict between competing stress responses J Bacteriol 2002 184 806 811 10.1128/JB.184.3.806-811.2002 11790751 PMC139526

[B41] Martin G Elena SF Lenormand T Distributions of epistasis in microbes fit predictions from a fitness landscape model Nat Genet 2007 39 555 560 10.1038/ng1998 17369829

[B42] Whitlock MC Phillips PC Moore FB Tonsor SJ Multiple Fitness Peaks and Epistasis Annual Review of Ecology and Systematics 2003 26 601 629 10.1146/annurev.es.26.110195.003125

[B43] Driessen AJ Nouwen N Protein translocation across the bacterial cytoplasmic membrane Annu Rev Biochem 2008 77 643 667 10.1146/annurev.biochem.77.061606.160747 18078384

[B44] Gelis I Bonvin AM Keramisanou D Koukaki M Gouridis G Karamanou S Economou A Kalodimos CG Structural basis for signal-sequence recognition by the translocase motor SecA as determined by NMR Cell 2007 131 756 769 10.1016/j.cell.2007.09.039 18022369 PMC2170882

[B45] Keseler IM Bonavides-Martinez C Collado-Vides J Gama-Castro S Gunsalus RP Johnson DA Krummenacker M Nolan LM Paley S Paulsen IT EcoCyc: a comprehensive view of Escherichia coli biology Nucleic Acids Res 2009 37 D464 470 10.1093/nar/gkn751 18974181 PMC2686493

[B46] Charollais J Dreyfus M Iost I CsdA, a cold-shock RNA helicase from Escherichia coli, is involved in the biogenesis of 50S ribosomal subunit Nucleic Acids Res 2004 32 2751 2759 10.1093/nar/gkh603 15148362 PMC419605

[B47] Borden JR Jones SW Indurthi D Chen Y Papoutsakis ET A genomic-library based discovery of a novel, possibly synthetic, acid-tolerance mechanism in Clostridium acetobutylicum involving non-coding RNAs and ribosomal RNA processing Metab Eng 2010 12 268 281 10.1016/j.ymben.2009.12.004 20060060 PMC2857598

[B48] Ochi K From Microbial Differentiation to Ribosome Engineering Bioscience, Biotechnology, and Biochemistry 2007 71 1373 1386 10.1271/bbb.70007 17587668

[B49] He X Qian W Wang Z Li Y Zhang J Prevalent positive epistasis in Escherichia coli and Saccharomyces cerevisiae metabolic networks Nat Genet 2010 42 272 276 10.1038/ng.524 20101242 PMC2837480

[B50] Yamada J Yamasaki S Hirakawa H Hayashi-Nishino M Yamaguchi A Nishino K Impact of the RNA chaperone Hfq on multidrug resistance in Escherichia coli J Antimicrob Chemother 2010 65 853 858 10.1093/jac/dkq067 20211861

[B51] Salis HM Mirsky EA Voigt CA Automated design of synthetic ribosome binding sites to control protein expression Nat Biotechnol 2009 27 946 950 10.1038/nbt.1568 19801975 PMC2782888

[B52] Crombach A Hogeweg P Evolution of evolvability in gene regulatory networks PLoS Comput Biol 2008 4 e1000112 10.1371/journal.pcbi.1000112 18617989 PMC2432032

[B53] Philippe N Crozat E Lenski RE Schneider D Evolution of global regulatory networks during a long-term experiment with Escherichia coli Bioessays 2007 29 846 860 10.1002/bies.20629 17691099

[B54] Jovelin R Phillips PC Evolutionary rates and centrality in the yeast gene regulatory network Genome Biol 2009 10 R35 10.1186/gb-2009-10-4-r35 19358738 PMC2688926

[B55] Santos CN Stephanopoulos G Combinatorial engineering of microbes for optimizing cellular phenotype Curr Opin Chem Biol 2008 12 168 176 10.1016/j.cbpa.2008.01.017 18275860

[B56] Bleichert F Baserga SJ The long unwinding road of RNA helicases Mol Cell 2007 27 339 352 10.1016/j.molcel.2007.07.014 17679086

[B57] Waters LS Storz G Regulatory RNAs in bacteria Cell 2009 136 615 628 10.1016/j.cell.2009.01.043 19239884 PMC3132550

[B58] Maharjan R Zhou Z Ren Y Li Y Gaffe J Schneider D McKenzie C Reeves PR Ferenci T Wang L Genomic identification of a novel mutation in hfq that provides multiple benefits in evolving glucose-limited populations of Escherichia coli J Bacteriol 2010 192 17 4517 21 Epub 2010 Jun 11. 10.1128/JB.00368-10 20543067 PMC2937378

[B59] Wang L Spira B Zhou Z Feng L Maharjan RP Li X Li F McKenzie C Reeves PR Ferenci T Divergence involving global regulatory gene mutations in an E. coli population evolving under phosphate limitation Genome Biology and Evolution 2010 evq035 10.1093/gbe/evq035 PMC2997555 20639316

[B60] Fraser HB Hirsh AE Steinmetz LM Scharfe C Feldman MW Evolutionary rate in the protein interaction network Science 2002 296 750 752 10.1126/science.1068696 11976460

[B61] Lu C Zhang Z Leach L Kearsey MJ Luo ZW Impacts of yeast metabolic network structure on enzyme evolution Genome Biol 2007 8 407 10.1186/gb-2007-8-8-407 17692134 PMC2374984

[B62] Guillier M Gottesman S Storz G Modulating the outer membrane with small RNAs Genes Dev 2006 20 2338 2348 10.1101/gad.1457506 16951250

[B63] Tsui HC Leung HC Winkler ME Characterization of broadly pleiotropic phenotypes caused by an hfq insertion mutation in Escherichia coli K-12 Mol Microbiol 1994 13 35 49 10.1111/j.1365-2958.1994.tb00400.x 7984093

[B64] Hobbs EC Astarita JL Storz G Small RNAs and small proteins involved in resistance to cell envelope stress and acid shock in Escherichia coli: analysis of a bar-coded mutant collection J Bacteriol 2010 192 59 67 10.1128/JB.00873-09 19734312 PMC2798238

[B65] Baars L Ytterberg AJ Drew D Wagner S Thilo C van Wijk KJ de Gier JW Defining the role of the Escherichia coli chaperone SecB using comparative proteomics J Biol Chem 2006 281 10024 10034 10.1074/jbc.M509929200 16352602

[B66] Hasona A Zuobi-Hasona K Crowley PJ Abranches J Ruelf MA Bleiweis AS Brady LJ Membrane composition changes and physiological adaptation by Streptococcus mutans signal recognition particle pathway mutants J Bacteriol 2007 189 1219 1230 10.1128/JB.01146-06 17085548 PMC1797365

[B67] Gonzalez R Tao H Purvis JE York SW Shanmugam KT Ingram LO Gene array-based identification of changes that contribute to ethanol tolerance in ethanologenic Escherichia coli: comparison of KO11 (parent) to LY01 (resistant mutant) Biotechnol Prog 2003 19 612 623 10.1021/bp025658q 12675606

[B68] Koo JT Choe J Moseley SL HrpA, a DEAH-box RNA helicase, is involved in mRNA processing of a fimbrial operon in Escherichia coli Mol Microbiol 2004 52 1813 1826 10.1111/j.1365-2958.2004.04099.x 15186427

[B69] Kazuta Y Adachi J Matsuura T Ono N Mori H Yomo T Comprehensive analysis of the effects of Escherichia coli ORFs on protein translation reaction Mol Cell Proteomics 2008 7 1530 1540 10.1074/mcp.M800051-MCP200 18453339 PMC2500233

[B70] Aono R Kobayashi H Cell surface properties of organic solvent-tolerant mutants of Escherichia coli K-12 Appl Environ Microbiol 1997 63 3637 3642 9293016 10.1128/aem.63.9.3637-3642.1997 PMC168671

[B71] Yoshimura M Oshima T Ogasawara N Involvement of the YneS/YgiH and PlsX proteins in phospholipid biosynthesis in both Bacillus subtilis and Escherichia coli BMC Microbiol 2007 7 69 10.1186/1471-2180-7-69 17645809 PMC1950310

[B72] Cao Y Yang J Xian M Xu X Liu W Increasing unsaturated fatty acid contents in Escherichia coli by coexpression of three different genes Appl Microbiol Biotechnol 2010 87 271 280 10.1007/s00253-009-2377-x 20135119

[B73] Ezeji T Milne C Price ND Blaschek HP Achievements and perspectives to overcome the poor solvent resistance in acetone and butanol-producing microorganisms Appl Microbiol Biotechnol 2010 85 1697 1712 10.1007/s00253-009-2390-0 20033401

[B74] Potrykus K Cashel M (p)ppGpp: still magical? Annu Rev Microbiol 2008 62 35 51 10.1146/annurev.micro.62.081307.162903 18454629

[B75] Aoki KF Kanehisa M Using the KEGG database resource Curr Protoc Bioinformatics 2005 Chapter 1 Unit 1 12 18428742 10.1002/0471250953.bi0112s11

[B76] Johnson M Zaretskaya I Raytselis Y Merezhuk Y McGinnis S Madden TL NCBI BLAST: a better web interface Nucleic Acids Res 2008 36 W5 9 10.1093/nar/gkn201 18440982 PMC2447716

[B77] Chen K Wallis JW McLellan MD Larson DE Kalicki JM Pohl CS McGrath SD Wendl MC Zhang Q Locke DP BreakDancer: an algorithm for high-resolution mapping of genomic structural variation Nat Methods 2009 6 677 681 10.1038/nmeth.1363 19668202 PMC3661775

[B78] Koressaar T Remm M Enhancements and modifications of primer design program Primer3 Bioinformatics 2007 23 1289 1291 10.1093/bioinformatics/btm091 17379693

[B79] You FM Huo N Gu YQ Luo MC Ma Y Hane D Lazo GR Dvorak J Anderson OD BatchPrimer3: a high throughput web application for PCR and sequencing primer design BMC Bioinformatics 2008 9 253 10.1186/1471-2105-9-253 18510760 PMC2438325

[B80] Zuker M Mfold web server for nucleic acid folding and hybridization prediction Nucleic Acids Res 2003 31 3406 3415 10.1093/nar/gkg595 12824337 PMC169194

[B81] Miller JH A short course in bacterial genetics: a laboratory manual and handbook for Escherichia coli and related bacteria 1992 Plainview, N.Y.: Cold Spring Harbor Laboratory Press

[B82] Baba T Ara T Hasegawa M Takai Y Okumura Y Baba M Datsenko KA Tomita M Wanner BL Mori H Construction of Escherichia coli K-12 in-frame, single-gene knockout mutants: the Keio collection Mol Syst Biol 2006 2 2006 0008 10.1038/msb4100050 PMC1681482 16738554

[B83] Rouillard JM Zuker M Gulari E OligoArray 2.0: design of oligonucleotide probes for DNA microarrays using a thermodynamic approach Nucleic Acids Res 2003 31 3057 3062 10.1093/nar/gkg426 12799432 PMC162330

[B84] The_R_Development_Core_Team R: A language and environment for statistical computing 2010 R Foundation for Statistical Computing, Vienna, Austria

[B85] Huber W von Heydebreck A Sultmann H Poustka A Vingron M Variance stabilization applied to microarray data calibration and to the quantification of differential expression Bioinformatics 2002 18 Suppl 1 S96 104 12169536 10.1093/bioinformatics/18.suppl_1.s96

[B86] Bengtsson H Jonsson G Vallon-Christersson J Calibration and assessment of channel-specific biases in microarray data with extended dynamical range BMC Bioinformatics 2004 5 177 10.1186/1471-2105-5-177 15541170 PMC539274

[B87] Bengtsson H Hossjer O Methodological study of affine transformations of gene expression data with proposed robust non-parametric multi-dimensional normalization method BMC Bioinformatics 2006 7 100 10.1186/1471-2105-7-100 16509971 PMC1534066

[B88] Bengtsson H Irizarry R Carvalho B Speed TP Estimation and assessment of raw copy numbers at the single locus level Bioinformatics 2008 24 759 767 10.1093/bioinformatics/btn016 18204055

[B89] Tukey JW Exploratory data analysis 1977 Reading: Addison-Wesley Pub. Co

[B90] Smyth GK Gentleman R, Carey V, Dudoit S, Irizarry R, Huber W Limma: linear models for microarray data Bioinformatics and Computational Biology Solutions using R and Bioconductor 2005 New York: Springer 397 420 full_text

[B91] Smyth GK Linear models and empirical bayes methods for assessing differential expression in microarray experiments Stat Appl Genet Mol Biol 2004 3 Article3 16646809 10.2202/1544-6115.1027

[B92] Loennstedt L Speed TP Replicated microarray data Statistica Sinica 2002 12

[B93] Benjamini Y Hochberg Y Controlling the false discovery rate: a practical and powerful approach to multiple testing Journal of the Royal Statistical Society Series B 1995 57 289 300

[B94] Liao JC Boscolo R Yang YL Tran LM Sabatti C Roychowdhury VP Network component analysis: reconstruction of regulatory signals in biological systems Proc Natl Acad Sci USA 2003 100 15522 15527 10.1073/pnas.2136632100 14673099 PMC307600

[B95] Galbraith SJ Tran LM Liao JC Transcriptome network component analysis with limited microarray data Bioinformatics 2006 22 1886 1894 10.1093/bioinformatics/btl279 16766556

[B96] Gama-Castro S Jimenez-Jacinto V Peralta-Gil M Santos-Zavaleta A Penaloza-Spinola MI Contreras-Moreira B Segura-Salazar J Muniz-Rascado L Martinez-Flores I Salgado H RegulonDB (version 6.0): gene regulation model of Escherichia coli K-12 beyond transcription, active (experimental) annotated promoters and Textpresso navigation Nucleic Acids Res 2008 36 D120 124 10.1093/nar/gkm994 18158297 PMC2238961

[B97] Ausubel FM Current protocols in molecular biology 1987 Brooklyn, N.Y. Media, Pa.: Greene Publishing Associates; J. Wiley, order fulfillment

[B98] Oh TJ Kim IG Park SY Kim KC Shim HW NAD-dependent malate dehydrogenase protects against oxidative damage in Escherichia coli K-12 through the action of oxaloacetate Environmental Toxicology and Pharmacology 2002 11 9 14 10.1016/S1382-6689(01)00093-X 21782581

