# Supplementary material for: Evolution combined with genomic study elucidates genetic bases of isobutanol tolerance in Escherichia coli
Source: Microb Cell Fact. 2011 Mar 25;10:18. doi: 10.1186/1475-2859-10-18 (PMC3071312; doi:10.1186/1475-2859-10-18)
Supplement: Additional file 3 — Tracing mutations found in endpoint populations through intermediate generations. We investigated the dynamics of genotypic adaptation in the G3 and X3 lineages by genotyping population samples from intermediate generations for selected mutations identified in the end point populations. Genotyping was conducted by screening whole-population cryopreserved samples for mutations with Sanger sequencing of PCR amplified mutated regions (for G3 mdh and miaA-hfq mutations), inferred from PCR product size for large insertion mutations (marC transposon insertions, X3 mdtj::IS5::tqsA, and G3 glnE::IS186) or using allele specific PCR (all other genotyped mutations). WT designates wild-type allele (directly detected in Sanger sequencing or inferred from lack of allele specific PCR product), Mut designates mutant allele (directly detected in Sanger sequencing or inferred from of amplification of allele specific PCR product), NT designates not tested. Strength (strong, weak, etc) indicates band intensity on agarose gel electrophoresis of PCR product, and is roughly correlated with allele frequency (allele specific PCR and large insertions). Sanger sequencing and genotyping via PCR product sizes allow discrimination of mixed genotypes, which are reported where applicable. [file 1475-2859-10-18-S3.DOCX]

| Population | Passage | ~Generation | **Genotypes** | | | | | | | |
| --- | --- | --- | --- | --- | --- | --- | --- | --- | --- | --- |
|  |  |  | **acrB** | **marC** | **mdh** | **hfq** | **rph** | **groL** | **gatZ** | **glnE** |
| Glucose #3 | 0 | 0 | WT | WT | WT | WT | WT | WT | WT | WT |
| Glucose #3 | 5 | 37.5 | WT | Mixed WT/Mut | WT | WT | WT | WT | WT | WT |
| Glucose #3 | 10 | 75 | WT | Mut | WT | WT/mut mixed | WT | WT | WT | WT |
| Glucose #3 | 16 | 120 | WT | Mut | WT | WT/mut mixed; mut dominant | Mut (very faint) | WT | WT | WT |
| Glucose #3 | 20 | 150 | WT | Mut | Mut | Mut | Mut (strong) | WT | WT | WT |
| Glucose #3 | 25 | 187.5 | WT | Mut | Mut | Mut | Mut (Strong) | Mut (strong) | WT | WT |
| Glucose #3 | 30 | 224 | NT | Mut | NT | NT | NT | NT | Mut (very weak) | Mixed WT/Mut |
| Glucose #3 | 35 | 262.5 | WT | Mut | Mut | Mut | Mut (Strong) | Mut (strong) | Mut (Extremely faint) | Mixed WT/Mut |
| Glucose #3 | 65 | 487.5 | Mut (strong) | Mut | Mut | Mut | Mut (Strong) | Mut (Strong) | Mut (strong) | Mixed WT/Mut |

| Population | Passage | ~Generation | **Genotypes** | | | | | | | | | | |
| --- | --- | --- | --- | --- | --- | --- | --- | --- | --- | --- | --- | --- | --- |
|  |  |  | **hrpA** | **yfgO** | **marC** | **mdh** | **gatC** | **plsX** | **acrA** | **rph** | **deaD** | **rpsB** | **mdtj-tqsA** |
| Xylose #3 | 0 | 0 | WT | WT | WT | WT | WT | WT | WT | WT | WT | WT | WT |
| Xylose #3 | 5 | 37 | Mut | WT | Mixed WT/Mut | WT | Mut (Extremely faint) | WT | WT | WT | WT | WT | WT |
| Xylose #3 | 10 | 75 | Mut | Mut? Extremely faint | Mixed WT/Mut | WT | Mut (faint) | WT | WT | WT | WT | WT | WT |
| Xylose #3 | 15 | 112 | Mut | Mut | Mut | WT | Mut (faint) | WT | WT | WT | WT | Mut (very faint) | WT |
| Xylose #3 | 20 | 149 | Mut | Mut | Mut | WT | Mut | WT | WT | WT | WT | Mut | WT |
| Xylose #3 | 25 | 186 | Mut | Mut | Mut | WT | Mut | WT | WT |  | WT | Mut | Mixed WT/Mut (WT dominant) |
| Xylose #3 | 35 | 261 | Mut | Mut | Mut | Mut (strong) | Mut | Mut (Faint) | WT | WT | Mut (Strong) | Mut (strong) | Mixed WT/Mut |
| Xylose #3 | 57 | 425 | Mut | Mut | Mut | Mut (strong) | Mut | Mut | Mut (Strong) | Mut (Strong) | Mut (strong) | Mut (strong) | Mixed WT/Mut |
