# Supplementary figures and images for: Evolution combined with genomic study elucidates genetic bases of isobutanol tolerance in Escherichia coli
Source: Microb Cell Fact. 2011 Mar 25;10:18. doi: 10.1186/1475-2859-10-18 (PMC3071312; doi:10.1186/1475-2859-10-18)

**A****rpoD Normalized qRT-PCR Expression**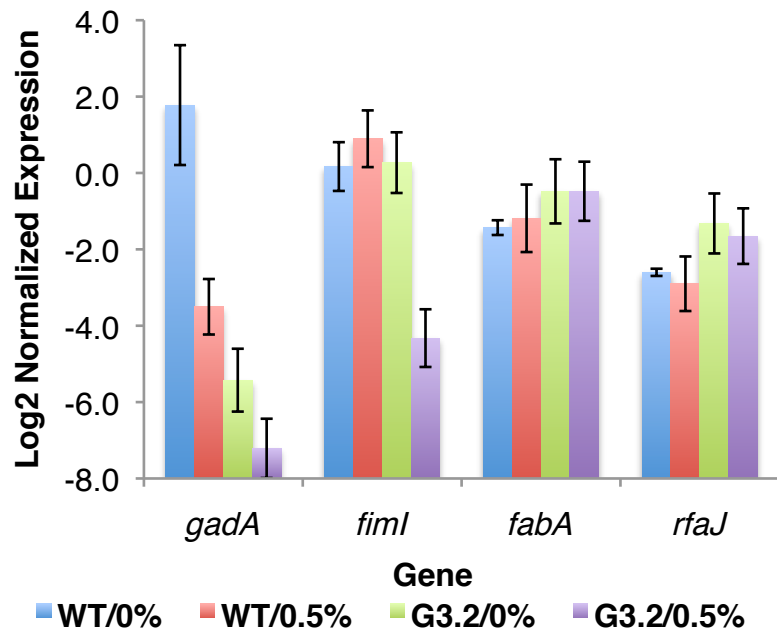**B****Microarray Expression**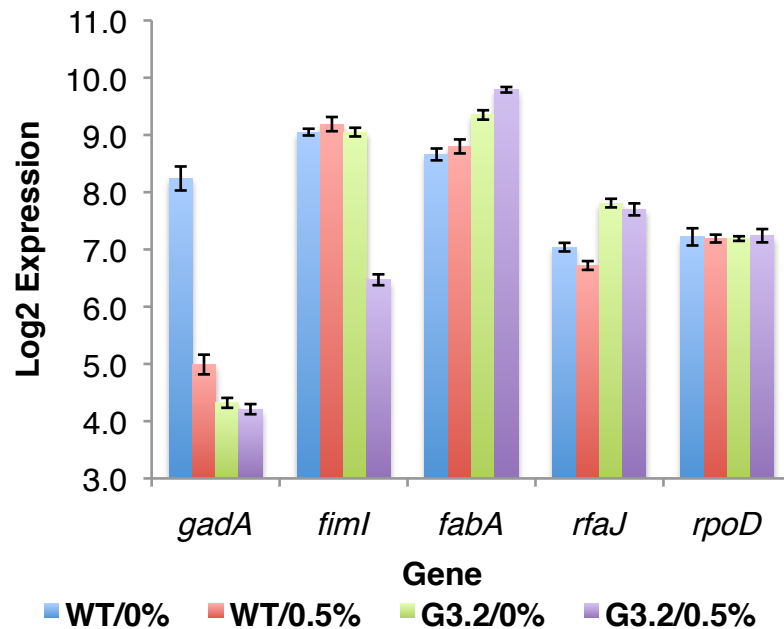

Supplement: Additional file 5 — qRT-PCR validation of gadA, fimI, fabA, and rfaJ gene expression changes. qRT-PCR was used to validate gene expression changes measured by DNA microarray. Target concentrations were determined by fitting the MAK2 PCR model to qRT-PCR data [31]. Expression levels were normalized to house keeping gene rpoD (sigma factor 70). (A) rpoD normalized expression levels determined by qRT-PCR (B) Expression levels from DNA microarray study. [file 1475-2859-10-18-S5.PDF]

A

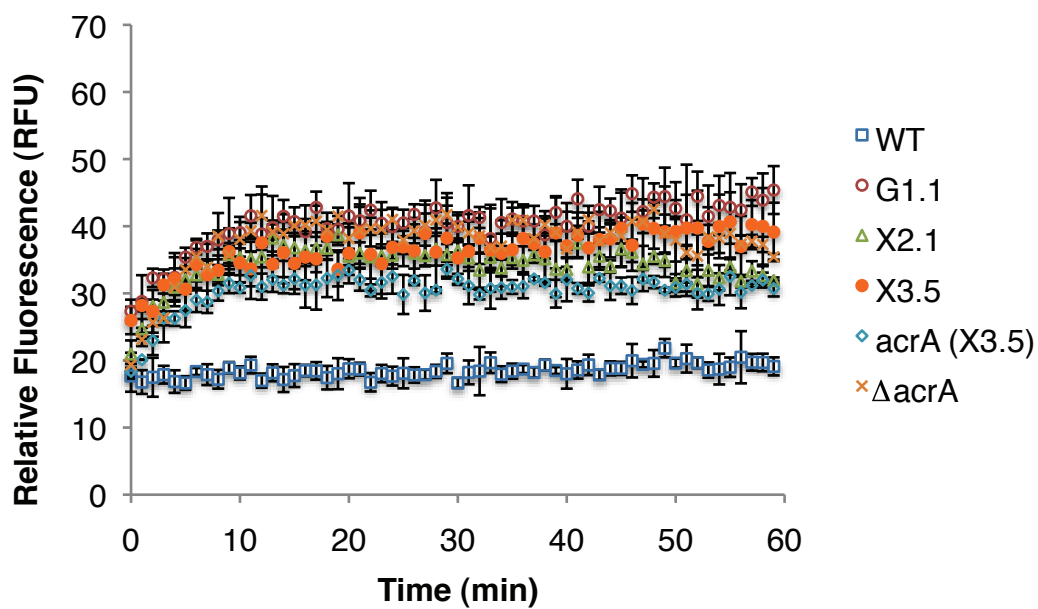

B

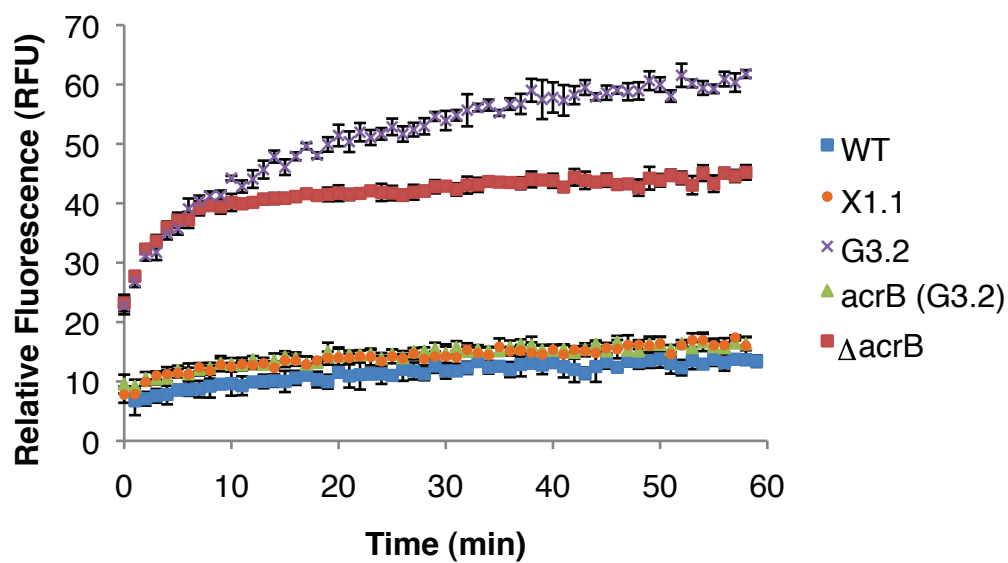

C

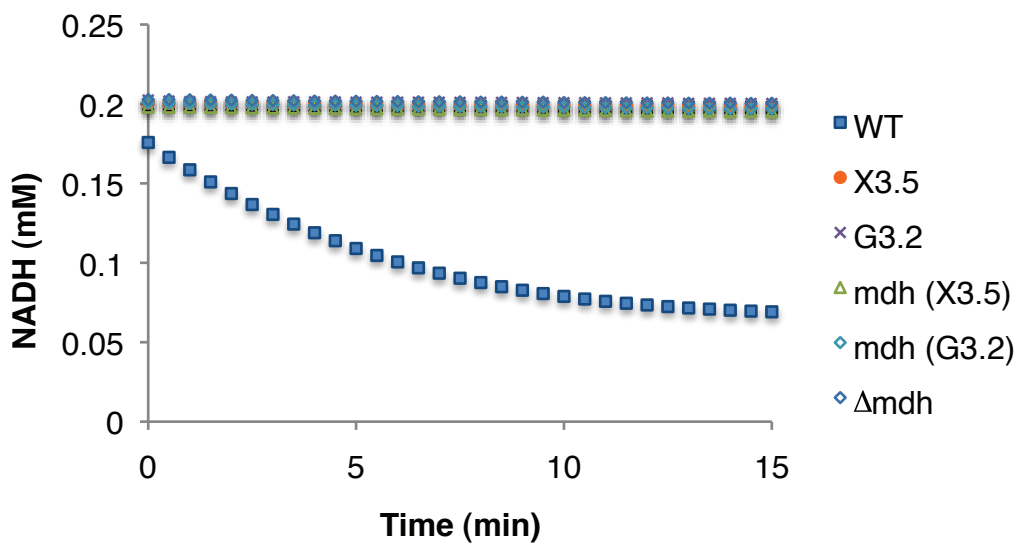

Supplement: Additional file 6 — acrAB and mdh functional assays. AcrAB-TolC efflux pump activity was measured via ethidium bromide (EtBr) accumulation in reconstructed single mutants and clonal isolates harbouring acrAB mutations from evolution end populations. Mid log phase cells were incubated with ethidium bromide and intracellular ethidium bromide was monitored via relative fluorescence (518 nm excitation/605 nm emission). Mdh (NADH dependent malate dehydrogenase) activity was assayed by incubating cell extracts with oxaloacetate and NADH; disappearance of NADH (due to reduction of oxaloacetate to malate) was monitored by measuring absorbance at 340 nm. (A) EtBr accumulation assay for the parent E. coli EcNR1 (WT), clonal isolates from evolution end populations harbouring acrA mutations (G1.1, X2.1, X3.5), a reconstructed acrA single mutant (containing mutation found in X3.5), and ΔacrA::kan control. (B) EtBr accumulation assay for the parent E. coli EcNR1 (WT), clonal isolates from evolution end populations harbouring acrB mutations (X1.1, G3.2), a reconstructed acrB single mutant (containing mutation found in G3.2), and ΔacrB::kan control. (C) Mdh assay for the parent E. coli EcNR1 (WT), clonal isolates from evolution end populations harbouring mdh mutations (G3.2, X3.5), reconstructed mdh single mutants (containing mutations found in G3.2 or X3.5), and Δmdh::kan control. [file 1475-2859-10-18-S6.PDF]
